# Current Progress on Methods and Technologies for Catalytic Methane Activation at Low Temperatures

**DOI:** 10.1002/advs.202204566

**Published:** 2022-12-11

**Authors:** François Nkinahamira, Ruijie Yang, Rongshu Zhu, Jingwen Zhang, Zhaoyong Ren, Senlin Sun, Haifeng Xiong, Zhiyuan Zeng

**Affiliations:** ^1^ State Key Laboratory of Urban Water Resource and Environment Shenzhen Key Laboratory of Organic Pollution Prevention and Control School of Civil and Environmental Engineering Harbin Institute of Technology Shenzhen Shenzhen 518055 P. R. China; ^2^ Department of Materials Science and Engineering City University of Hong Kong 83 Tat Chee Avenue Kowloon Hong Kong 999077 P. R. China; ^3^ State Key Laboratory of Physical Chemistry of Solid Surfaces College of Chemistry and Chemical Engineering Xiamen University Xiamen 361005 P. R. China

**Keywords:** C—H bond activation, low‐temperature, methane activation, noble metal‐based catalysts, transition metal‐based catalysts

## Abstract

Methane (CH_4_) is an attractive energy source and important greenhouse gas. Therefore, from the economic and environmental point of view, scientists are working hard to activate and convert CH_4_ into various products or less harmful gas at low‐temperature. Although the inert nature of C—H bonds requires high dissociation energy at high temperatures, the efforts of researchers have demonstrated the feasibility of catalysts to activate CH_4_ at low temperatures. In this review, the efficient catalysts designed to reduce the CH_4_ oxidation temperature and improve conversion efficiencies are described. First, noble metals and transition metal‐based catalysts are summarized for activating CH_4_ in temperatures ranging from 50 to 500 °C. After that, the partial oxidation of CH_4_ at relatively low temperatures, including thermocatalysis in the liquid phase, photocatalysis, electrocatalysis, and nonthermal plasma technologies, is briefly discussed. Finally, the challenges and perspectives are presented to provide a systematic guideline for designing and synthesizing the highly efficient catalysts in the complete/partial oxidation of CH_4_ at low temperatures.

## Graphical abstract

This review summarizes the current methods and technologies for the catalytic oxidation of CH_4_ at low temperatures. It focuses on the noble metals and transition metal‐based catalysts that activate CH_4_ in temperatures ranging from 50 to 500 °C. Then, it briefly discusses the partial oxidation of CH_4_ at relatively low temperatures through thermocatalysis, photocatalysis, electrocatalysis, and nonthermal plasma technologies.

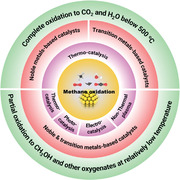

## 1 Introduction

Methane (CH_4_) is the principal constituent of natural gas and significant greenhouse gas.^[^ ^1^, ^2^ ^]^ At the same time, CH_4_ is also an excellent carbon‐based fuel due to the lesser CO_2_ released per unit of energy, high thermal efficiency, and ability to adapt to standard engines.^[^ ^3^ ^]^ Different efforts have been made to activate CH_4_. However, the results are not satisfactory due to the inert property of the C—H bonds, which requires high dissociation and ionization energies, nonpolar tetrahedral geometry, which makes it difficult to be adsorbed on catalysts, and the absence of secondary/tertiary C—H bonds.^[^ ^2^, ^4^ ^]^ Thus, the C—H bonds are commonly activated through oxidation, requiring high energy at a high temperature.^[^ ^5^ ^]^

Direct and indirect technologies, like steam/dry reforming, partial oxidation, and oxidative/nonoxidative coupling, have been developed to activate and transform CH_4_ into intermediate organic compounds (e.g., CH_3_OH and CH_3_COOH), syngas as well as complete oxidation to form CO_2_ and H_2_O.^[^ ^6^, ^7^ ^]^ However, these technologies present considerable limitations such as high price, heavy consumption of energy, and the issues related to catalysts’ thermal stability, reusability, reproducibility, lifetime reduction, and generation of pollutants such as NO*_x_*.^[^ ^8^ ^]^ Hence, research on the catalytic oxidation of CH_4_ at low‐temperature represents excellent opportunities to overcome these limits. In addition, low‐temperature oxidation of CH_4_ can provide economical and environmentally friendly ways to produce valuable chemicals with low energy consumption and minimize the pollutants emitted into the atmosphere. Furthermore, low‐temperature oxidation can prevent coke formation and the rapid deactivation of the catalysts. Here, low‐temperature CH_4_ oxidation refers to the activation of CH_4_ using an oxidant at temperatures below 500 °C.

Generally, CH_4_ can be activated by using noble metals or transition metal oxides‐based catalysts where the active sites of a catalyst are deposited onto a high surface area support for increased metal dispersion and decreased metal loading.^[^ ^9^ ^]^ On these solid surfaces, the activation of CH_4_ can occur via direct dissociative or precursor‐mediated mechanisms.^[^ ^10^ ^]^ As for the direct dissociative mechanism, the C—H bond split occurs when CH_4_ collides with the surface of catalysts. The CH_4_ adsorbed on the surface undergoes C—H bond dissociation for the precursor‐mediated pathway. Therefore, a kinetic competition between the desorption of CH_4_ and the cleavage of the C—H bond is dominated by the state of the catalyst's surface as well as the temperature/pressure of the reaction. Therefore, the precursor‐mediated mechanism is more likely to happen at low temperatures. However, the low‐temperature catalytic activation of CH_4_ is a significant issue because the active surface of the catalyst could be easily poisoned via the oxidation of the active catalyst by O_2_, H_2_O, or SO_2_, which are predominant oxidants or residues in the system.^[^ ^9^, ^11^, ^12^, ^13^, ^14^, ^15^ ^]^

Over the last five years, significant efforts have been initiated to create catalysts with active site protection.^[^ ^16^ ^]^ Hence, a balance between catalyst activity and its tolerance to toxic chemicals is critical for the low‐temperature catalyst for CH_4_ activation. These efforts have developed numerous new catalysts for the low‐temperature oxidation of CH_4_. Therefore, it is time to review and discuss the recent progress on methods and technologies for catalytic oxidation of CH_4_ at low temperatures, pointing out the reaction mechanisms, knowledge acquired in developing new catalysts, and future perspectives with brief promising solutions. Notably, there is an ever‐increasing effort in partial oxidation and catalytic transformation of CH_4_ into valuable products, as recently reviewed.^[^ ^7^, ^17^, ^18^, ^19^ ^]^ Hence, this contribution addresses homogeneous and heterogeneous catalysts, focusing on heterogeneous catalysts with complete CH_4_ oxidation. In addition, this contribution does not cover biocatalysts due to the limited space, and excellent reviews on this topic have been recently published.^[^ ^20^, ^21^, ^22^ ^]^ Moreover, little attention was paid to photocatalysis, electrocatalysis, and nonthermal plasma, as recent review articles in these fields are available in the literature.^[^ ^23^, ^24^, ^25^, ^26^, ^27^, ^28^, ^29^ ^]^ Furthermore, the readers interested in the theoretical perspective of CH_4_ activation are referred to a relevant review on this topic.^[^ ^2^ ^]^

**Figure 1:**
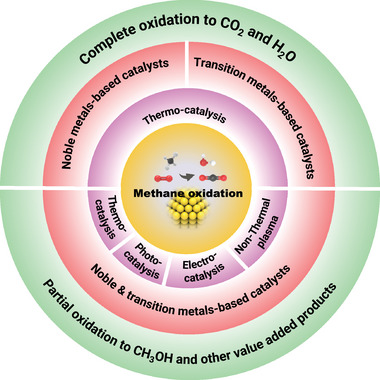
A summary of methods and technologies for catalytic CH_4_ oxidation at low temperatures.

The catalysts were divided into two categories based on their CH_4_ activation temperatures, as summarized in **Figure** **1**. The first category focused on the catalysts activating CH_4_ from 50 to 500 °C, while the second category emphasized on CH_4_ activation at relatively low temperatures. In addition, catalytic activities were expressed using the temperatures necessary to achieve 10 (T_10_), 50 (T_50_), and 90% (T_90_) CH_4_ conversions. For the sake of readability, catalysts in the first category were reviewed based on active sites and support materials. This review started with noble metals and transition metal‐based catalysts, activating CH_4_ from 50 to 500 °C. After that, it briefly discussed the activation of CH_4_ at relatively low temperatures based on thermocatalysts that partially oxidize CH_4_ in the liquid‐phase, photocatalysis, electrocatalysis, and nonthermal plasma technologies. Finally, the challenges and future perspectives were presented at the end of this review. Such information can offer systematic direction for rational invention and synthesis of highly efficient catalysts to win this battle against the 21st‐century holy grail of chemistry and catalysis.

## 2 Thermocatalytic Activation of Methane

### 2.1 Methane Activation over Noble Metal‐Based Catalysts

Because of their outstanding activity, noble metals like Pd, Pt, Rh, and Au have been intensively researched as catalysts for CH_4_ activation. The Pd and Pt‐based catalysts are the most documented, where Pd is significantly more efficient than Pt under lean conditions.^[^ ^9^ ^]^ Identifying the catalytic sites at an atomic level is critical for basic knowledge about the reaction mechanisms. Thus, different characterization studies suggested that Pt could activate the nonpolar C—H bonds of CH_4_ via a homolytic pathway. By contrast, Pd‐supported catalysts usually follow the classical Mars–van Krevelen (MvK) pathway that CH_4_ is accumulated on the surface of Pd species, dissociated, and dehydrogenated to make CH_3_* radicals; and then CH_3_* radicals combine with O in PdO to produce CO_2_ and H_2_O as the final products.^[^ ^2^, ^30^ ^]^ Therefore, Pd‐based catalysts have attracted the most attention for the CH_4_ activation at relatively low temperatures.^[^ ^31^ ^]^ However, there are still issues related to the agglomeration of small nanoparticles, sintering, and high surface energies.^[^ ^32^ ^]^ Hence, many attempts have been tried to maintain the active sites in small particle sizes and avoid their sintering and mobility during the reaction.^[^ ^33^ ^]^

#### 2.1.1 Pd‐Based Catalysts

**Figure 2:**
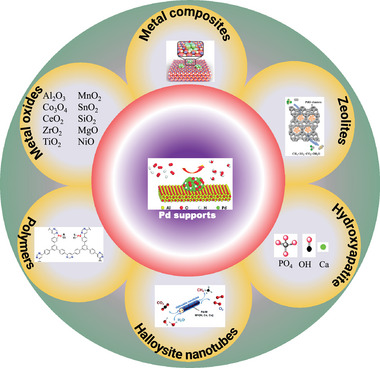
Supports for Pd‐based catalysts for CH_4_ oxidation at low temperatures.

Pd is the most effective catalyst for CH_4_ oxidation at lower temperatures.^[^ ^34^ ^]^ It is well known that CH_4_ activation is greatly influenced by Pd particle size, chemical state, and support properties.^[^ ^35^ ^]^ In addition, PdO was revealed to be the more active phase at lower temperatures as it forms between 300 and 400 °C.^[^ ^3^ ^]^ However, there are still contradictory data for Pd and PdO activities for CH_4_ activation.^[^ ^36^ ^]^ More studies are still needed to understand that contradiction. In addition, the Pd‐based catalysts carried on different materials like metal oxides and composites have been reported for CH_4_ combustion, as shown in **Figure** **2**.^[^ ^37^, ^38^, ^39^ ^]^

However, Pd catalysts supported on metal oxides also have a reputation for being unstable at low‐temperature due to water/hydroxyl inhibition that can hinder the active sites and deactivate the catalyst.^[^ ^40^, ^41^ ^]^ Except the water poison, SO_2_ impurities can permanently disable the Pd‐based catalysts by forming inactive PdSO_3_ or PdSO_4_, preventing Pd from reoxidizing to PdO, and avoiding the creation of the low‐temperature active phase, PdO*_x_*.^[^ ^42^, ^43^ ^]^ Therefore, to limit the buildup of hydroxyl groups on the surface and to prevent the production of Pd(OH)_2_ and PdSO_4_, supports with high oxygen mobility and less effective for sulfate formation at low temperatures are needed.^[^ ^34^, ^44^, ^45^, ^46^ ^]^

##### Pd Catalyst Supported on Single Metal Oxide: Pd Catalysts Supported on Al_2_O_3_

**Figure 3:**
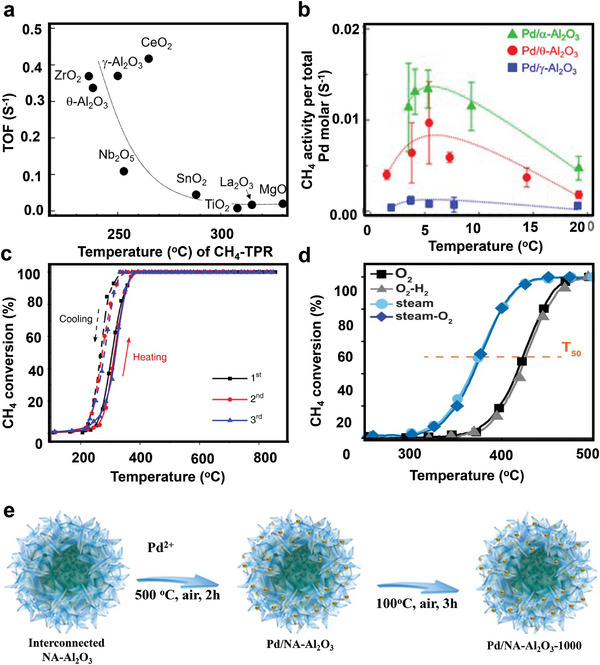
a) TOFs versus CH_4_‐TPR peak temperature for 7 nm Pd supported on different metal oxides. Reproduced with permission.^[^ ^49^ ^]^ Copyright 2020, American Chemistry Society. b) CH_4_ oxidation performance per total Pd molar at 350 °C. Reproduced with permission.^[^ ^50^ ^]^ Copyright 2020, American Chemistry Society. Temperature‐dependent of CH_4_ combustion over various catalysts. c) Pd/NA‐Al_2_O_3_. Reproduced under the terms of the Creative Commons Attribution 4.0 International License.^[^ ^33^ ^]^ Copyright 2022, The Authors, published by Springer Nature. d) Pd/Al_2_O_3_ after steam (600 °C), O_2_–H_2_, and CO–O_2_–H_2_ pretreatments. Reproduced with permission.^[^ ^57^ ^]^ Copyright 2021, AAAS. e) Cross‐linked assembly Pd active phases stabilized by NA‐Al_2_O_3_ at 1000 °C in the air. Reproduced under the terms of the Creative Commons Attribution 4.0 International License.^[^ ^33^ ^]^ Copyright 2022, The Authors, published by Springer Nature.

Al_2_O_3_ is commonly used as single support due to its surface with Lewis's acid sites that significantly affect the distribution, state of the active components, and catalytic performance in different reactions. In addition, it has been clarified that moderately acidic oxides support materials enhance the electron deficiency of noble metals more than strongly acidic supports.^[^ ^47^ ^]^ As a result, Al_2_O_3_ support has been demonstrated to affect the temperature shift of the Pd–PdO phase transformation and its temperature hysteresis for nanosized Pd.^[^ ^48^ ^]^ For instance, using Pd of 7 nm particle size carried on different oxides such as *θ*‐Al_2_O_3_, TiO_2_, and MgO, the temperature dependence of the CH_4_ conversion showed the light‐off temperature of *θ*‐Al_2_O_3_ to be 100 °C lower compared to Pd/TiO_2_ and Pd/MgO. In addition, Pd/*γ*‐Al_2_O_3_, Pd/*θ*‐Al_2_O_3_, Pd/ZrO_2_, and Pd/CeO_2_ demonstrated volcanic trend turnover frequencies (TOFs) as Pd particle size increased, with the highest TOF value at 7 nm. However, compared to the other supported Pd catalysts, Pd/TiO_2_ and Pd/MgO were less effective for all particle sizes.^[^ ^49^ ^]^ As a result, the activity of CH_4_ combustion is considerably affected by the characteristics of the support and the Pd particle size, as illustrated in **Figure** **3**a,b, and different efforts to lower the activation temperature of CH_4_ using various catalytic supports have been made. It is important to note that the correlation between activity and reducibility implies that the support does not directly affect CH_4_ combustion but rather adjusts the redox characteristics of Pd.^[^ ^49^ ^]^

Murata et al. investigated how the Al_2_O_3_ crystalline phase and Pd particle size affected the CH_4_ oxidation in wet conditions at 300 and 600 °C. Under wet conditions, CH_4_ oxidation over 5.4 nm Pd/*θ*‐Al_2_O_3_ and 9.3 nm Pd/*α*‐Al_2_O_3_ sparked at ≈300 °C and achieved ≈100% at 450 °C. The performance of 9.3 nm Pd/*α*‐Al_2_O_3_ for CH_4_ combustion was nearly equal to that of 5.4 nm Pd/*θ*‐Al_2_O_3_ despite the larger Pd particle size. On the contrary, at 600 °C, the CH_4_ performance over the 3.7 nm Pd/*γ*‐Al_2_O_3_ catalyst was ≈90%. Hence, the hydrophobic *α*‐Al_2_O_3_ support considerably increased the CH_4_ oxidation activity under wet conditions.^[^ ^50^ ^]^ The same results were reported when the 0.2–2 wt% Pd was impregnated on Al_2_O_3_ with different morphology and the treatment at 800, 850, or 900 °C in the presence of air for 10 h led to the generation of Pd/Al_2_O_3_ catalysts with varying Pd particles sizes.^[^ ^49^, ^51^ ^]^ Due to the changes in the strength of metal–support interaction, the crystalline phase of alumina influences the shape of the Pd particle. By controlling the size of the Pd particles, weak interaction between Pd and *θ*‐Al_2_O_3_ or *α*‐Al_2_O_3_ provides spherical Pd particles with a high proportion of step sites.^[^ ^51^ ^]^ Meanwhile, Yang et al. recently dispersed ultrafine Pd active phases on a nanosheet‐assembled *γ*‐Al_2_O_3_ (NA‐Al_2_O_3_) architecture via compartmentalization to synthesize a robust nanocatalyst (Pd/NA‐Al_2_O_3_) as shown in Figure 3e. Surprisingly, the catalytic performance of Pd/NA‐Al_2_O_3_ was slightly better after thermal annealing and showed complete oxidation of CH_4_ around 300 °C^[^ ^33^ ^]^ (Figure 3c), which is 300 °C lower than the result reported by Murata et al. using *γ*‐Al_2_O_3_.^[^ ^50^ ^]^ Notably, Pd/NA‐Al_2_O_3_ still performed better than Pd/*θ*‐Al_2_O_3_ and *α*‐Al_2_O_3_ in wet conditions at 350 °C (**Table** **1**). The NA‐Al_2_O_3_ structures, with distinct flower‐like shapes, displayed notable structural benefits for rapid mass transport and simple accessibility to active sites. The obvious increase and/or encapsulation of PdO at high temperatures led to a significant loss of activity for the Al_2_O_3_ nanosheet supported catalyst (Pd/N‐Al_2_O_3_) after 1000 °C annealing.^[^ ^33^ ^]^ Yang et al.’s results challenge the conclusion of Murata et al. concerning the role of *α*‐Al_2_O_3_ and Pd/*θ*‐Al_2_O_3_ hydrophobicity in increasing CH_4_ oxidation activity under wet conditions.^[^ ^33^, ^50^ ^]^

**Table 1:** Summary of Pd‐based catalysts supported on single metal oxide for CH_4_ activation

Catalyst	Metal loading [wt%]	Preparation method	Calc. temp. [°C]	Dispersion [%]	Particle size [nm]	CH_4_ conc. [%]	Catalyst amount [mg]	GHSV [mL g h^−1^]	T_10_ [°C]	T_50_ [°C]	T_90_ [°C]	Refs.
Pd/NAAl_2_O_3_	5	Compartmentalization	500	20.1		1.0	200	15 000	235	260	300	[33]
Pd/*θ*‐Al_2_O_3_	0.34	Colloidal method	700	16	7	0.4	20	300 000	250	330	400	[49]
Pd/*α*‐Al_2_O_3_	2	Impregnation	800	12	9.3	0.4	200	300 000	300	375	450	[50]
Pd/ADP‐OMA	0.5	Ultrasound‐assisted sol–gel	900	39.5	2.8	1.0	100	50 000	330	385	430	[108]
Pd/*γ*‐Al_2_O_3_	3.3	Impregnation‐vortexing	250	32.9	3–10	1.0	100	90 000	230	252	275	[52]
Pd/Al_2_O_3_	0.5	One pot	800	80.4	1–2	1.0	100	50 000	315	360	420	[53]
Pd/o–CeO_2_–S2	1.68	Reductive deposition	400	–	–	1.0	20	60 000	250	305	348	[74]
Pd/CeO_2_(HHA)	0.77	Reduction– deposition	450	30	11.8	1.0	200	15 000	270	302	336	[75]
Pd/CeO_2_ (IMP)	0.79	Incipient wetness impregnation	450	15	11.7	1.0	200	15 000	295	368	440	
Pd/CeO_2_‐MS	0.5	Impregnation	450	62.33	0.3	1.0	150	110 000	245	318	397	[73]
Pd/CeO_2_	2	Deposition–precipitation	400	1.46	–	1.0	500	50 000	224	257	300	[109]
Pd/Co_3_O_4_	5	Incipient wetness impregnation	350		6.3	2.0	200	40 000	215	245	248	[84]
Pd/Co_3_O_4_ (LT)	3.21	Light treatment	350	4	11.6	10	50	30 000	217	277	314	[86]
Pd/Co_3_O_4_‐P	2.14	Incipient wetness impregnation	350	11.2	10.1		200	51 600	219	284	337	[85]
Pd‐Co_3_O_4_	0.98	In situ redox process	500	46.2	2.2	1.0	100	30 000	247	291	337	[88]
Pd/ZrO_2_900‐DC	1.5	Coprecipitation	900	12.0	9.3	0.1	–	–	285	335	390	[91]
PdO/MnO_2_	1.4	Deposition–precipitation	400		13	–	100	180 000		286	330	[40]
Pd/SiO_2_‐SEA	3.7	Strong electrostatic adsorption	600	73	1.8	0.5	100	50 000	287	341	409	[110]
Pd/SnO_2_(1200)	0.15	Impregnation	600	15.5	7.0	1.0	200	–	265	320	360	[103]
PT‐1000	0.5	Incipient wetness impregnation	500	–	3.4	1.0	100	30 000	290	330	360	[3]

To examine the impact of calcination temperature on Pd/*γ*‐alumina catalysts, various methodologies were used to assess particle sizes, surface species, and activity in CH_4_ oxidation. Fertal et al. recently synthesized 3.3 wt% Pd/Al_2_O_3_ catalysts via a new impregnation‐vortexing method with calcination at 150, 250, and 500 °C. 3.3 wt% Pd/Al_2_O_3_ showed the light‐off temperature varying between 250 and 255 °C for all catalysts. Surprisingly, under lean CH_4_ conditions, 100% CH_4_ oxidation was completed at 275 °C in 20 min, with the sample calcinated at 250 °C. Additionally, XPS revealed PdO*_x_* and PdO on the catalyst's surface, with some PdO*_x_* being converted to PdO and Pd^0^ after the reaction. As revealed by different characterizations, the catalyst calcined at 250 °C (3Pd/Al_2_O_3_ 250 °C) had the maximum dispersion, which was reduced following the reaction because of particle growth. The results also confirmed that PdO*_x_* was the active phase of Pd/*γ*‐Al_2_O_3_ in the CH_4_ oxidation at temperatures below 300 °C.^[^ ^52^ ^]^ As shown in Table 1, Pd/*γ*‐Al_2_O_3_ reported by Fertal et al. performed better than all Pd supported on Al_2_O_3_ with T_10_, T_50_, and T_90_ of 230, 252, and 275 °C, respectively. Even if its performance was assigned to the high dispersion (32.9%) and the absence of dormant monodentate carbonate species, which may prevent water desorption, its behaviors in the presence of water and other poisons are still unknown. In addition, Pd/Al_2_O_3_ prepared via the one‐pot method showed the highest dispersion of 80.4%;^[^ ^53^ ^]^ however, its catalytic performance is low compared to Pd/*γ*‐Al_2_O_3_ synthesized by the impregnation‐vortexing method.^[^ ^52^ ^]^ It is worth mentioning that catalytic performance is affected by different factors, which are not standardized across various studies; hence, making in‐depth comparisons for the catalysts reported is still challenging. Therefore, it is suggested to perform profound studies correlating factors with homogenous measurements through machine learning and artificial intelligence.

Mesoporous alumina (MA) has a consistent pore architecture that provides a high specific surface area and adjustable pore size, which is beneficial to the dispersion of active sites.^[^ ^54^, ^55^ ^]^ Thus, Chen et al. applied the acoustic cavitation effect of the sonochemical method to produce MA. The obtained MA showed a regular and thermally stable spherical structure, which was used as carriers for Pd catalysts. Results confirmed that the pore size distribution of MA has an obvious effect on the activity of the catalyst, with 90% CH_4_ conversion at 490 °C for the sample with a small pore size (5.1 nm, Pd/MA‐5.1) and large specific surface area (284 m^2^ g^−1^). By contrast, the T_90_ of the other two catalysts with a pore size of 7.1 and 8.5 nm are 525 and 565 °C, respectively. Notably, the catalyst's calcination temperature has no discernible effect on Pd/MA‐5.1, which is attributed to the “confinement effect” of smaller pore size, limiting the Pd particle movement and sintering when the catalyst undergoes high‐temperature calcination. On the other hand, a high specific surface area helps to disperse the active components and supplies numerous active sites for the reaction, which improves catalytic performance. Nevertheless, the treatment temperature of the supports significantly affected the efficiency of the Pd/MA‐5.1 catalyst. The Pd/MA‐5.1 catalyst calcined at 1000 °C shows the T_90_ of 470 °C, which is 20 °C lesser than the catalyst calcined at 800 °C.^[^ ^56^ ^]^ Meanwhile, defects can exhibit high reactivity due to the specific atomic structure that differs from crystalline surfaces. The high‐temperature steam pretreatment of Pd catalysts improved the mass‐specific reaction rate for C—H activation in CH_4_ oxidation by a factor of 12 over conventional pretreatments, as shown in Figure 3d.^[^ ^57^ ^]^

Other different catalysts prepared by different pretreatments were reported to decrease the activation temperature of CH_4_ and inhibit the effect of water. For instance, the catalyst prereduction significantly increased the catalytic performance during the light‐off, regardless of the gas composition used in the exhaust of lean‐burn gas engines.^[^ ^58^ ^]^ Before the activity test, the catalysts were prereduced with H_2_ for 30 min at 400 °C, which significantly shifted the light‐off toward lower temperatures. Although the light‐off curve commencement for Pd/Al_2_O_3_ overlaps in the oxidized and prereduced states, pretreatment moves T_50_ in a dry reaction gas mixture by 20 °C.^[^ ^58^ ^]^ In addition, the wet reaction mixture demonstrates a robust water inhibitory effect for Pd/Al_2_O_3_ with T_50_ moved by >110 °C toward higher temperatures. Interestingly, the light‐off/light‐out cycles of various reducing agents such as H_2_, CH_4_, and CO did not exhibit any noticeable differences in activity, which was attributed to the high temperature (400 °C) used for reduction.^[^ ^59^ ^]^ After the reductive pretreatment, the operando X‐ray absorption spectroscopy demonstrated a completely reduced catalyst state that reoxidized with raising the temperature in a lean reaction mixture, yielding bulk PdO at 350 °C.^[^ ^58^ ^]^ Over a Pd/Al_2_O_3_ catalyst, the influence of oxygen concentration on CH_4_ oxidation was also investigated. It was discovered that raising the amount of oxygen in a dry atmosphere led to higher CH_4_ oxidation activity at a lower temperature, which is linked to PdO formation.^[^ ^60^ ^]^ Mihai et al. reported a 50% CH_4_ conversion with 1200, 1000, and 800 ppm oxygen concentrations for 289, 321, and 335 °C, respectively. Interestingly, the inhibitory result of water on the Pd‐catalyst was significantly greater at high oxygen concentrations in the gas mixture (500 ppm CH_4_, 8% O_2_, 5% H_2_O) compared to that at lower oxygen levels (800–1200 ppm), leading to the hypothesis that a high oxygen excess facilitates the production of the hydroxyl species, which inhibits the active sites.^[^ ^60^ ^]^ Meanwhile, results showed that the presence of inorganic gases such as CO_2_ and CO on the Pd/Al_2_O_3_ catalyst causes a negligible reduction in the catalytical activity.^[^ ^61^, ^62^ ^]^ In the absence of sulfur‐containing compounds, nitrogen‐containing compounds augment CH_4_ conversion, but ammonia has the reverse effect when sulfur compounds are present.^[^ ^63^ ^]^ Recently, it was revealed that NO*_x_* could shield against sulfur and hydrothermal deactivation, which can harm CH_4_ oxidation catalysts. NO, and NO_2_ hinder active sites on PdO due to strong adsorption, which immediately degrades on a non‐SO_2_ poisoned catalyst. For nonpoisoned catalysts with CO*_x_*, the light‐off temperature (T_50_) was 406 °C, while it was 428 °C for nonpoisoned catalysts with CO*_x_* and NO. On a poisoned catalyst, NO_2_ forms HNO_2_, which efficiently eliminates hydrogen from hydroxyl groups on the catalyst surface.^[^ ^64^ ^]^

Pd/Al_2_O_3_ catalysts are also deactivated with CH_4_ gas streams.^[^ ^40^, ^41^, ^65^ ^]^ Therefore, some reports focused on enhancing the activity of Pd/Al_2_O_3_ in those conditions at low‐temperature.^[^ ^66^ ^]^ Coney et al. investigated the paths of reaction inhibition and their interactions with the heat and mass transport phenomena of a 3%Pd/Al_2_O_3_ wash‐coated monolith. At temperatures above 400 °C, the water inhibition process is partially reversible, with minor levels of deactivation noticed when exposed to water vapor. The production of Pd(OH)*_x_* species under humid conditions was also attributed to the reversible inhibitory effect of water.^[^ ^67^ ^]^ Another study found that the activity of Pd/Al_2_O_3_ can be increased through repeated short reducing pulses (SRP) in wet lean CH_4_ oxidation leading to much lower apparent activation energy than static operation. The use of kinetic oxidation models to fit time‐resolved operando XAS data indicated that only moderately active PdO exists under static operation. By contrast, during SRP operation, metallic Pd forms and maintains highly active PdO*_x_* species. Finally, to maintain high CH_4_ conversion, a practical range of Pd oxidation degree (25–65% Pd content) was proposed.^[^ ^66^ ^]^ It is known that preparation methods, pretreatment of catalysts, and reaction conditions significantly contribute to lowered CH_4_ activation temperature by designing catalysts with different morphology that affect particle size and its distribution on Al_2_O_3_ support and increase the oxygen storage capacity of the catalyst. Therefore, other single metal oxide support materials known to reduce the negative effect of water have also been studied to reduce the activation temperature of CH_4_.^[^ ^68^ ^]^

##### Pd Catalysts Supported on CeO_2_

Ceria (CeO_2_) is considered essential catalyst support due to its high adsorption capacity and specific redox performance to store and release oxygen. Additionally, it is known to boost supported Pd's catalytic performance and lessen water's harmful effects, most likely due to the potent interaction between CeO_2_ and PdO.^[^ ^34^ ^]^ Performance of the Pd catalyst supported on CeO_2_ (Pd/CeO_2_) correlated with the structure and morphology of CeO_2_ and the preparation method of the catalyst.^[^ ^69^, ^70^ ^]^ As seen in Figure 3a–f, various CeO_2_ morphologies disclose crystal faces and atomic structures, which alter the interaction between the support and the loaded metal.^[^ ^71^ ^]^ Lei et al. investigated the interaction between the CeO_2_ crystal faces and the Pd species loaded on various morphological CeO_2_ crystals. The results showed that the bare CeO_2_ support exhibited poor catalytic activities of 14.5%, 8%, and 3.8% for rod‐CeO_2_(r‐CeO_2_), cube‐CeO_2_(c‐CeO_2_), and octahedral‐CeO_2_(o‐CeO_2_), respectively, at 450 °C. Even though the activity of o‐CeO_2_ was the worst, Pd loaded on o‐CeO_2_ displayed the highest activity with 90% conversion at 348 °C.^[^ ^72^ ^]^ In the study by Chen et al., the Pd/CeO_2_‐oct and Pd/CeO_2_‐cube catalysts exhibit a positive resistance to water with an activity loss of ≈8% after a 24 h test.^[^ ^71^ ^]^ Furthermore, the Ce^3+^/Ce^4+^ ion pairs were crucial in producing low‐concentration CH_4_ by adsorbing or releasing oxygen. Thus, the amount of Ce^3+^ on the surface of o‐CeO_2_ support was significantly improved due to the presence of Pd species.^[^ ^72^ ^]^ Similarly, Guo et al. confirmed the morphology effect of CeO_2_ support where their results showed that the Pd/CeO_2_‐microsphere (Pd/CeO_2_‐MS) catalyst performed better than Pd/CeO_2_‐nanotube (Pd/CeO_2_‐NT). For Pd/CeO_2_‐MS catalyst, the T_90%_ is 397 °C. The reaction temperatures were reduced by 47 °C for Pd/CeO_2_‐NT catalysts.^[^ ^73^ ^]^ In addition, CeO_2_‐MS support was more advantageous to Pd particle dispersion. It was confirmed that specific crystal faces played an important role in lowering the activation temperature of CH_4_ with Pd/CeO_2_ catalysts with different morphologies.

Furthermore, Wu et al. examined the activities of Pd‐based catalysts with various Pd phases (Pd or PdO) produced by changing the calcination condition while excluding the effects of crystal structure, morphology, and particle size. The X‐ray absorption characterization combined with light‐off curves affirmed that suitable catalyst activity was highly related to the proportion of the metallic Pd and PdO species. The T_90_ CH_4_ conversion for static air calcinated samples (Pd/CeO_2_S‐*x*, where *x* stands for calcination time) were 415, 370, 349, 368, and 390 °C for Pd/CeO_2_S‐0.5, Pd/CeO_2_S‐1, Pd/CeO_2_S‐2, Pd/CeO_2_S‐3, and Pd/CeO_2_S‐5, respectively.^[^ ^74^ ^]^ Thus, within limits, increasing Pd^0^/PdO ratio can decrease the activation temperature of CH_4_. Jian et al. reported similar results, using the reduction–deposition and impregnation method (IMP) to make 3 Pd/CeO_2_ catalysts with various chemical and electronic states of Pd species, using hydrazine hydrate (HHA) and formaldehyde (FA) as reductants, respectively. Because of the highest PdO and adsorbed oxygen concentrations on the surface, Pd/CeO_2_ (HHA) has the maximum activity for CH_4_ combustion with 336 °C as T_90_, which is about 104 and 226 °C lower compared to Pd/CeO_2_ (IMP) and Pd/CeO_2_ (FA), respectively.^[^ ^75^ ^]^ Even if hydrazine hydrate as a reductant provided promising results, it is worth mentioning that it is a toxic substance, and selecting it as a reagent requires careful consideration of its properties.^[^ ^76^ ^]^ From an environmental point of view, catalysts should be prepared with less hazardous substances, making their application easy on an industrial scale. The effect of the synthesis technique, the pore size of the supports, and Pd loading on the activity for CH_4_ total oxidation were examined by impregnating porous glasses with cerium nitrate and palladium nitrate solution, followed by further calcination. At 350 °C, the catalyst synthesized with a glass with a pore diameter of 151 nm and a Pd loading of 0.63 wt% completely converted CH_4_, whereas a traditionally prepared Pd/CeO_2_ reference required 415 °C. Surprisingly, the CH_4_ conversion for a sample with 3.07 and 5.96 wt% Pd loading was similar.^[^ ^77^ ^]^

The nature of the active site and the role of doping Pd in the ceria surface in generating highly active sites toward the C—H bond activation in CH_4_ were also studied using density functional theory (DFT). The findings suggested that Pd^2+^ ions are active centers in the catalytic oxidation of CH_4_^[^ ^71^ ^]^ and that two Pd^2+^ ions can replace one Ce^4+^ ion, causing a highly stable structure capable of adsorbing CH_4_ and dissociating the first C—H bond with a low energy shield.^[^ ^72^, ^78^ ^]^ Furthermore, in situ FTIRS data show that the Mars–van Krevelen mechanism governs the CH_4_ oxidation reaction on PdO*_x_*/CeO_2_.^[^ ^71^, ^74^ ^]^ Moreover, Song et al.’s theoretical investigation revealed that the tetrahedral structure of CH_4_ was destroyed on the surface, resulting in the production of a —CH_3_ species attached to the Pd atom, which assists in the orbital hybridization between the C and Pd atoms.^[^ ^79^ ^]^ Moreover, the high CH_4_ conversion activity of PdO*_x_*/CeO_2_ was suggested to arise from emergent mixed oxide chemistry at the cluster/support interface. Mixing Pd into the CeO_2_ lattice provides a coordination environment for Pd^*δ*+^ species that enable facile alternation between Pd^2+^ and Pd^4+^ oxidation states, thereby creating highly reducible sites for CH_4_ activation.^[^ ^80^ ^]^ Among all methods for supporting Pd on CeO_2_, deposition–precipitation and reduction–deposition performed better than incipient wetness impregnation, as shown in Table 1. For instance, the T_90_ of Pd/CeO_2_ prepared by deposition–precipitation (Pd‐DP) was 300 °C, while it was 440 °C for the catalyst prepared by incipient wetness impregnation.^[^ ^75^ ^]^ Therefore, further investigations are required to understand the mechanism behind catalytic performance and water resistance of the Pd/CeO_2_ catalyst synthesized through the deposition–precipitation method.

Moreover, CeO_2_ was proposed as sulfating support that enables CH_4_ total oxidation at a much lower temperature and deactivates more slowly than commercial Pd/Al_2_O_3_ catalysts.^[^ ^16^, ^81^ ^]^ For instance, extremely thin Pd–CeO*_x_* nanowire (2.4 nm) catalysts facilitated complete CH_4_ oxidation at 350 °C, significantly less than a commercial Pd/Al_2_O_3_ catalyst (425 °C). In addition, CH_4_ conversion of Pd‐CeNW@SiO_2_ catalyst stayed at 100% throughout the 10 h test even after supplying the feed gas with SO_2_ and water vapor at 450 °C. The protecting effect of the SiO_2_ capsule hindered the sintering of active phases and the strong Pd–CeO_2_ interaction.^[^ ^16^ ^]^ To sum up, it is evident that the combination of Pd and ceria increases the stability and promotes the reoxidation of metallic Pd due to the strong synergetic effects and an enhancement in the dispersion of the active site leading to higher CH_4_ conversion and lowering of the reaction temperature.

##### Pd Catalysts Supported on Co_3_O_4_

**Figure 4:**
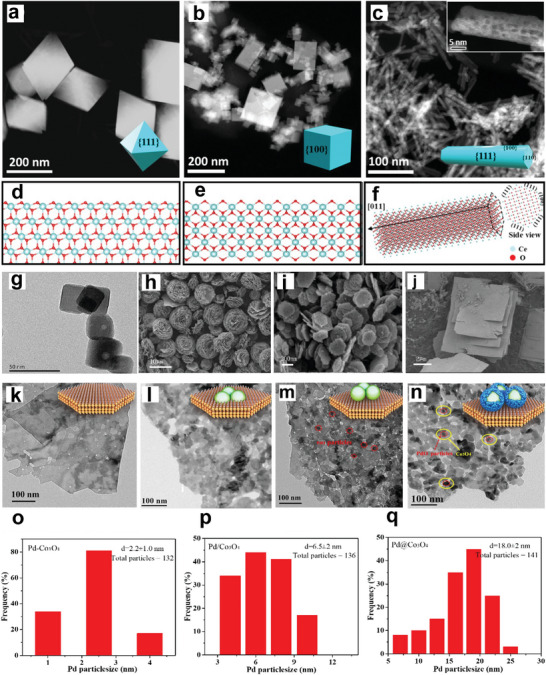
Structures and morphologies of the CeO_2_ and Co_3_O_4_ supports. a–c) Low‐magnification HADDF–STEM images of CeO_2_‐oct (111), CeO_2_‐cube (100), and CeO_2_‐rod. d–f) Atomic structure of CeO_2_‐oct (111), CeO_2_‐cube (100), and CeO_2_‐rod. Reproduced with permission.^[^ ^71^ ^]^ Copyright 2021, American Chemistry Society. g) TEM of Co_3_O_4_‐C, SEM of h) Co_3_O_4_‐F, i) Co_3_O_4_‐P, and j) Co_3_O_4_‐R. Reproduced with permission.^[^ ^85^ ^]^ Copyright 2017, Elsevier. TEM images of k) Co_3_O_4_, l) Pd–Co_3_O_4_, m) Pd/Co_3_O_4_, and n) Pd@Co_3_O_4_ catalysts. PdO particle size distributions of o) Pd–Co_3_O_4_, p) Pd/Co_3_O_4,_ and q) Pd@Co_3_O_4_ catalysts. Reproduced with permission.^[^ ^88^ ^]^ Copyright 2021, Elsevier.

Recently, significant efforts have been made to explore the fast redox property of Co_3_O_4_, increasing oxygen mobility and promoting active oxygen species’ formation by combining Co_3_O_4_ with Pd to favor the catalytic activation of CH_4_ at low temperatures.^[^ ^82^ ^]^ In contrast to most other reported promoters, cobalt oxide is an excellent catalyst for CH_4_ combustion, and its activity is shape‐dependent.^[^ ^83^ ^]^ Hence, Co_3_O_4_ is suggested to be the promising support for noble metals, promoting the catalytic activity of CH_4_. For instance, Pd catalysts supported on different Co_3_O_4_ have been prepared via the impregnation method, as shown in **Figure** **4**g–j.^[^ ^84^, ^85^ ^]^ Pd/Co_3_O_4_ nanosheets showed more promising results than Pd/Co_3_O_4_ nanobelts and nanocubes with the same Pd loading.^[^ ^86^ ^]^ For instance, the CH_4_ conversion at 250 °C for 5% Pd/Co_3_O_4_ nanosheets, nanobelts, and nanocubes are 95.4, 18.9, and 9.5%, respectively; while the T_90_ in the presence of 5% Pd/Co_3_O_4_ nanosheets was 248 °C, indicating that 5% Pd/Co_3_O_4_ nanosheets were the most effective catalyst despite its smallest surface area. Its exceptional activity is due to the strong surface Pd^2+^–cobalt–oxygen group interaction between the Co_3_O_4_ unit cell and the PdO unit cell on the {112} plane, as shown by CH_4_‐TPR tests and high‐resolution TEM (HRTEM). For 5% Pd/Co_3_O_4_ nanosheets, the as‐formed CO_2_ peaks centered at 212 and 369 °C shows a reduction of 17 and 21 °C to 5% Pd/Co_3_O_4_ nanobelts and by 32 and 41 °C compared to 5% Pd/Co_3_O_4_ nanocubes. CO_2_ produced over 5% Pd supported on Co_3_O_4_ nanosheets is higher than nanobelts and nanocubes. This indicates that the reduction of the support material by CH_4_ is increased when Pd is added and that Pd supported on Co_3_O_4_ nanosheets has a stronger reducing ability than Co_3_O_4_ nanobelts and Co_3_O_4_ nanocubes.^[^ ^84^ ^]^ The effect of Co_3_O_4_ morphology for lowering CH_4_ activation temperature was also reported by Chen et al. through the spinel cobalt oxide with various controllable morphologies (cubical ((Co_3_O_4_‐C), flower‐like (Co_3_O_4_‐F), plate‐like (Co_3_O_4_‐P), and rectangular‐like (Co_3_O_4_‐R) synthesized by facile hydrothermal method.^[^ ^85^ ^]^ The spinel cobalt oxides were loaded with Pd via incipient wetness impregnation. It was also demonstrated that the surface O_ads_/O_lat_ molar ratio of the catalyst and exposed crystal plane are necessary for CH_4_ combustion.^[^ ^87^ ^]^

According to Xiong et al., who prepared three different types of nanoplatelet‐like Co_3_O_4_‐supported Pd catalysts, embedded Pd species (Pd–Co_3_O_4_), loaded Pd species (Pd/Co_3_O_4_), and encapsulated Pd species (Pd@Co_3_O_4_), Pd species also showed to effect CH_4_ activation. The aim was to examine the effect of the existing states of Pd species on the catalytic oxidation of CH_4._ Moreover, the catalysts' crystal structure and morphology were explored using TEM and HRTEM. The obtained results suggested that Co_3_O_4_ support with 2D nanoplatelets structure (Figure 4k) slightly changed after the encapsulation of Pd species into Co_3_O_4_, as confirmed by Figure 4l–n. In addition, the PdO*_x_* species with an average particle diameter of 2.2 nm was reported for Pd–Co_3_O_4_ (Figure 4o). Furthermore, the catalytic activity was in the order of Pd–Co_3_O_4_ > Pd/Co_3_O_4_ > Pd@Co_3_O_4_ > Co_3_O_4_ with the T_90_ and T_99_ for Pd–Co_3_O_4_ at 337 and 345 °C, respectively. On the other hand, the T_90_ for Pd/Co_3_O_4_ catalysts (365 °C) was lower than that of Pd@Co_3_O_4_ catalysts (T_90_ = 372 °C).^[^ ^88^ ^]^ The low activation temperature of CH_4_ by Pd–Co_3_O_4_ catalyst was attributed to the strongest interaction between encapsulated PdO and Co_3_O_4_ support, best reducibility, more active adsorbed oxygen species, and easier diffusion and mobility of lattice oxygen; all these factors played a role in the activation of the C—H bond in CH_4_. The lower catalytic activity of the Pd@Co_3_O_4_ catalysts could be due to the presence of Co_3_O_4_, which partially hides the active PdO, lowering the exposed site.^[^ ^88^ ^]^

##### Pd Catalysts Supported on ZrO_2_

**Figure 5:**
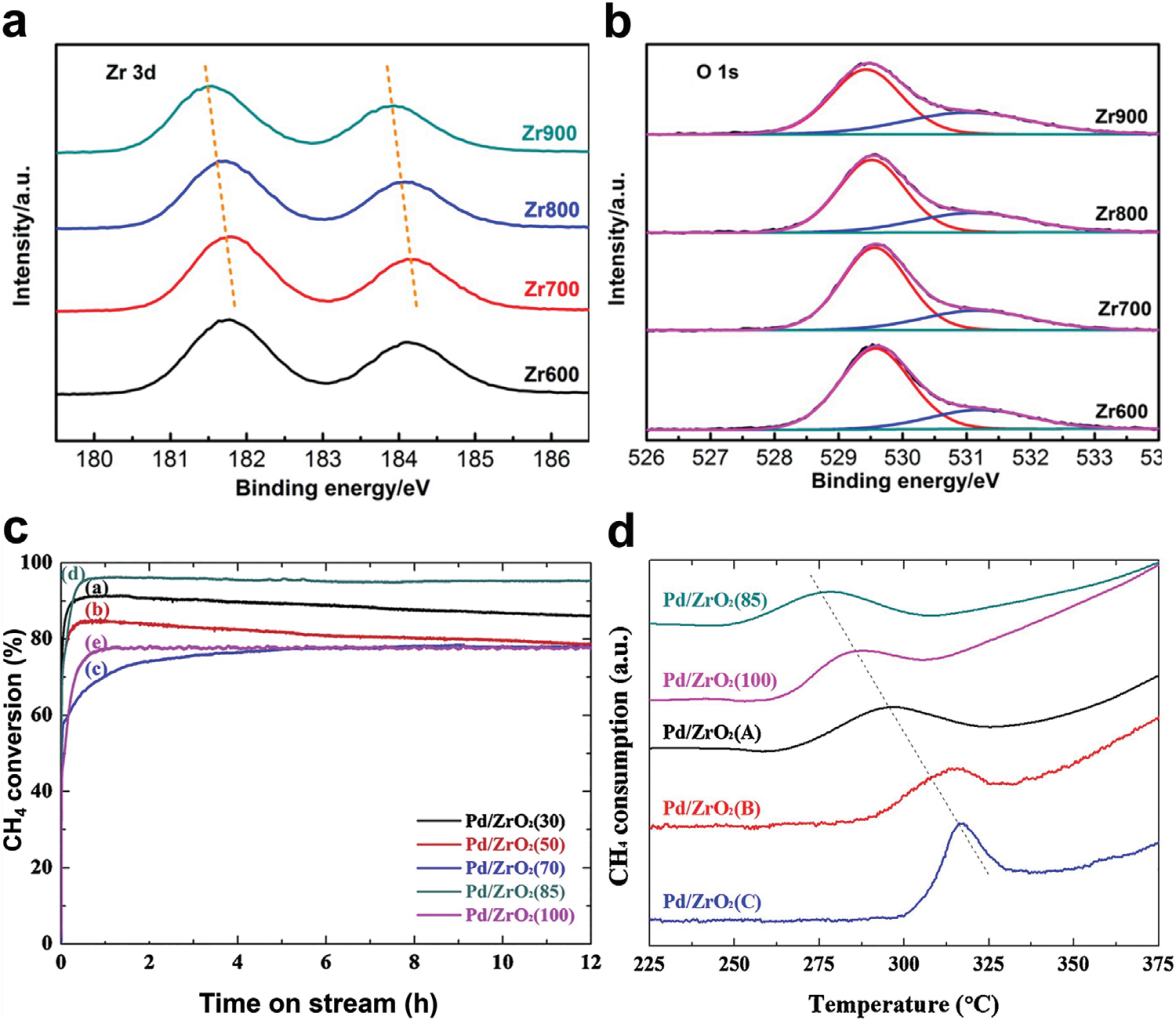
XPS a) Zr 3d spectra and b) O 1s spectra of the different. Reproduced with permission.^[^ ^91^ ^]^ Copyright 2019, Elsevier. c) Change in the catalytic activity of the Pd/ZrO_2_(*x*) in the CH_4_ oxidation reaction with time on stream in 10 vol% water vapors at 500. d) Reduction characteristics of the Pd/ZrO_2_ catalysts as exhibited by CH_4_‐TPR. Reproduced with permission.^[^ ^93^ ^]^ Copyright 2018, Elsevier.

The performance and stability of the Pd/ZrO_2_ catalysts for CH_4_ oxidation were reported to vary enormously with the support's calcination temperature.^[^ ^89^ ^]^ Furthermore, ZrO_2_’s surface characteristics and interactions with supporting metal oxides are directly connected to its phase structure.^[^ ^90^ ^]^ Thus, ZrO_2_ catalysts were synthesized by calcinating ZrO_2_·*x*H_2_O at 600 to 900 °C (*x* stands for calcination temperature). In addition, the oxide materials were used to make Pd/ZrO_2_ catalysts coated on the monolith.^[^ ^91^ ^]^ The results confirmed that the activity of the supported Pd catalysts is gradually increased by raising the calcination temperature of ZrO_2_ under dry conditions. To point out, the temperatures for 90% CH_4_ conversion of Pd/Zr900 compared with Pd/Zr600 decreased from 447 to 390 °C. However, water vapor has a significant inhibitory effect as 10 vol% H_2_O reduced T_90_ by 29–71 °C. The concentration of surface oxygen vacancies increased as the calcination temperature increased.^[^ ^92^ ^]^ The water poisoning considerably diminished the stimulating effect of increasing oxygen vacancies (**Figure** **5**a,b) on CH_4_ oxidation activity, resulting in slight variations in the activity of the catalysts.^[^ ^91^ ^]^ Furthermore, Hong et al. studied the impact of the crystalline structure and textural features of ZrO_2_ support on the CH_4_ oxidation reaction. The results revealed that as the digesting temperature was raised, the crystalline structure of ZrO_2_ changed from mixed‐phase to pure tetragonal, and the tetragonal ZrO_2_‐supported Pd catalysts demonstrated good hydrothermal stability (Figure 5c). Indeed, the CH_4_‐TPR profiles of the Pd/ZrO_2_ (Figure 5d) indicated that the reduction temperatures decreased as the particle size of the Pd species increased. Therefore, it is most likely owing to bigger PdO particles developing on the ZrO_2_(85) support, resulting in a PdO species that is highly reducible.^[^ ^93^ ^]^

##### Pd Catalysts Supported on Other Single Metal Oxides

Mn_2_O_3_ was proposed to support PdO because of its numerous stable oxidation states and exceptional oxygen storage capability. In addition, the various crystalline phases of manganese oxide with various metal coordination geometries also add value to catalytic activity.^[^ ^94^ ^]^ Thus, Dai et al. synthesized PdO supported by MnO_2_ nanowire through a deposition–precipitation approach.^[^ ^40^ ^]^ The T_50_ for produced PdO/MnO_2_ catalysts was 38 °C lower than PdO/Al_2_O_3_ catalysts when compared to Al_2_O_3_‐supported PdO catalysts (IWI). Pure MnO_2_ nanowires showed some catalytic activity with T_50_ at 418 °C. The increased activity was attributed to the MnO_2_ nanowire skeleton's high reducibility and exposure of active PdO facets. However, the catalysts demonstrated poor resistance to sulfur compounds in the flowing gas; initially, the catalysts appeared to be stable due to excess active sites, but the adverse effect of SO_2_ on the catalysts was severe.

Pd supported on silica (Pd/SiO_2_) has been widely used as a catalyst in environmental applications, and its performance often showed a pronounced dependence on the Pd size.^[^ ^95^ ^]^ Thus, controlling the Pd nanoparticle's size and distribution is of great importance for CH_4_ activation. While many methods were developed to prepare highly dispersed and well size‐distributed Pd/SiO_2_ catalysts, strong electrostatic adsorption was identified as the simplest and lowest‐cost method.^[^ ^96^ ^]^ The order of catalytic performance for CH_4_ oxidation agrees with Pd dispersion or Pd particle size, confirming that the activation temperature of CH_4_ decreases with an augmenting in the Pd dispersion or a reducing the Pd particle size.^[^ ^97^ ^]^ For instance, during CH_4_ combustion at 325 °C, the conversion levels for four catalysts were 4% for PdO/MCM‐41, 13% for PdO/MgO, 16% for PdO/TiO_2_, and 21% for PdO/Al_2_O_3_ after 20 min into CH_4_ combustion reaction. The lower performance for PdO/MCM‐41 was attributed to PdO smaller crystallite size of the MCM‐41. The smaller PdO particles have a higher interaction with the support material, limiting PdO reducibility by CH_4_.^[^ ^50^, ^98^ ^]^ The PdO/Al_2_O_3_ and PdO/MgO displayed unique trends of temporary deactivation, but the PdO/TiO_2_ and PdO/MCM‐41 did not deactivate in the 20 min reaction period; however, deactivation took place over a long period of time. Furthermore, the hydroxyl buildup per unit surface area of the PdO/MCM‐41 catalyst might contribute to the relatively slow rate of hydroxyl desorption from PdO/MCM‐41 compared to the other catalysts, as the hydroxyl deposition per unit surface area of the PdO/MCM‐41 is subsequently less than the other catalysts.^[^ ^98^, ^99^ ^]^ Thus, the ability to withstand water inhibition is more remarkable on the catalyst with high oxygen surface mobility to enhance oxygen exchange with the oxide support. Nevertheless, the correlation between augmenting oxygen mobility and reducing catalytic deactivation rate is complicated. For instance, MCM‐41 has lesser oxygen mobility than MgO and Al_2_O_3_, but PdO/MCM‐41 did not demonstrate any deactivation.^[^ ^68^ ^]^ XPS confirmed that water converts surface PdO to surface hydroxyls that postpone the generation of the active PdO phase in bulk.^[^ ^100^ ^]^

As the supports with better dehydroxylation capacity than Pd(OH)_2_ can remove toxic hydroxyls from Pd/PdO particles and enhance their performance in wet CH_4_ combustion;^[^ ^101^ ^]^ thus, SnO_2_ was proposed as a sink for hydroxyls extracted from Pd(OH)_2_.^[^ ^44^ ^]^ It was shown that Pd/SnO_2_ could surpass even Pd–Pt/Al_2_O_3_ in its activity in water at low temperatures.^[^ ^44^, ^102^ ^]^ In the wet lean CH_4_ combustion, Nassiri et al. tested Sn‐containing Pd catalysts with Pd/Al_2_O_3_ and PdPt/Al_2_O_3_. The goal was to determine whether SnO_2_ could potentially replace limited and expensive Pt, as well as which catalyst preparation method would provide the most significant advantage. The utilization of SnO_2_ as a support for Pd increased the catalyst's water resistance, as evidenced by the catalyst's unaltered behavior in 5% and 10% water feed conditions. When Sn was included in the catalytic material during colloidal synthesis of Pd nanoparticles, the Sn impact on CH_4_ conversion was not as significant as it would have been if SnO_2_ had been used as support. However, Pd activity enhanced to the point where Pt almost promoted it. Strong metal–support interactions and high oxygen mobility in SnO_2_ with dual Sn^4+^/Sn^2+^ valency explain the increased activity and stability compared to the conventional alumina support. SnO_2_ can be used as a support or promoter for Pd catalysts in lean wet CH_4_ combustion, allowing them to replace the costly and limited Pt.^[^ ^102^ ^]^ In addition, a kinetic study showed a more negative enthalpy of H_2_O adsorption on Pd/Al_2_O_3_, confirming the high‐water tolerance of Pd/SnO_2_ at low temperatures.^[^ ^101^ ^]^ Because of predicted lattice oxygen overflow, SnO_2_ reducibility was also essential in Pd/SnO_2_ activity, even without additional water.^[^ ^103^ ^]^ Recently, the catalyst synthesized from SnO_2_ calcined at 1200 °C (Pd/SnO_2_(1200)) showed outstanding activity and stability with complete combustion at 390 °C. Meanwhile, SnO_2_ calcined at 600 °C ((Pd/SnO_2_(600)) showed the same activity at 440 °C. Also, the reaction rate of CH_4_ on the Pd/SnO_2_(1200) was 35 times faster than that of Pd/SnO_2_(600). This is due to the lattice matching of PdO and SnO_2_, which allows oxygen to diffuse from SnO_2_ to vacant oxygen sites on the PdO/Pd surface, promoting CH_4_ catalytic combustion.^[^ ^103^ ^]^

TiO_2_ can improve metal dispersion due to its large specific surface area and potent affinity for noble metals.^[^ ^104^ ^]^ Moreover, as sulfating support, Ti‐containing catalysts display higher tolerance toward SO_2_ and H_2_O.^[^ ^105^ ^]^ Through an ultrasonic‐assisted sol–gel method, Ti was introduced into an alumina carrier. Results showed that the inclusion of Ti adjusts the charge distribution, resulting in the production of stable PdO particles and the improvement of PdO–support interactions. Additionally, the TiO_2_’s oxygen storing capability reduced the hysteresis between Pd reoxidation and PdO decomposition, stabilizing the PdO phase and speeding up the activation of CH_4_ with a 90% conversion at 410 °C.^[^ ^106^ ^]^ However, the poor stability of Pd/TiO_2_ catalysts continues to be a major issue.^[^ ^107^ ^]^ Recently, Xiao et al. reported that a Pd catalyst supported on a reducible carrier TiO_2_ via the sol–gel method and calcined 1000 °C could produce abundant Pd^2+^ species, Ti^3+^, and oxygen vacancies on the surface, assisting the reoxidation of Pd^0^ back to PdO and inducing a high activity toward combustion of CH_4_ to CO_2_ with a T_99_ at 370 °C. Nevertheless, some Pd^2+^ is oxidized to Pd^4+^ and then enters the TiO_2_ lattice, thereby diminishing oxygen exchange between bulk TiO_2_ and surface Pd species. As a result, the reoxidation capacity deteriorated, accompanied by a decrease in active PdO species, leading to reduced activity during on‐stream tests.^[^ ^3^ ^]^ Among all Pd‐based catalysts supported on single metal oxide, as summarized in Table 1, Pd supported on Co_3_O_4_ showed the lowest activation temperature for CH_4_. For instance, T_90_ of Pd/Co_3_O_4_ nanosheets,^[^ ^84^ ^]^ Pd/Co_3_O_4_ nanosheets (Pd/Co_3_O_4_ (LT)),^[^ ^86^ ^]^ Pd/Co_3_O_4_‐P,^[^ ^85^ ^]^ and Pd–Co_3_O_4_ (embedded Pd species)^[^ ^88^ ^]^ were 248, 314, 337, and 337 °C, respectively. However, those reports did not mention the impact of water and SO_2_. On the other hand, Pd/NAAl_2_O_3_ (catalyst prepared by compartmentalization),^[^ ^33^ ^]^ Pd/SnO_2_,^[^ ^102^ ^]^ and Pd/*α*‐Al_2_O_3_^[^ ^50^ ^]^ demonstrated T_90_ of 350, 420, and 450 °C in the water, respectively.

##### Pd Catalysts Supported on Metal Oxide Composite

To enhance the Pd dispersion and Pd^2+^/Pd ratio on the catalyst surface, some researchers supported Pd on the mixed oxides; the composite structure resulted in higher oxygen storage capacity and mobility than the pure solid solution phase and showed complete CH_4_ conversion at low temperature.^[^ ^32^, ^45^, ^111^, ^112^, ^113^ ^]^ Various promoters, such as CeO_2_, ZrO_2_, NiO, MgO, and Co_3_O_4_, have been investigated to prepare Pd‐based catalysts for CH_4_ oxidation.^[^ ^114^, ^115^ ^]^ CeO_2_ is known to supply oxygen for catalytic oxidation at low‐temperature by transmitting oxygen to Pd and enhancing the stability of the active PdO phase.^[^ ^116^ ^]^ On the other hand, pure ceria has weak temperature stability, and oxygen transport requires direct contact between the ceria and Pd.^[^ ^117^, ^118^ ^]^ To increase the Pd‐ceria interfacial contact, some scientists have created hierarchical catalysts in which Pd nanoparticles are initially produced and then engulfed by a very tiny porous ceria shell in close metal–support contact. These catalysts have demonstrated remarkable CH_4_ oxidation activity in dry environments.^[^ ^119^ ^]^ At temperatures below 400 °C and high space velocities, complete CH_4_ conversion was obtained on a Pd@CeO_2_/Si‐Al_2_O_3_ catalyst.^[^ ^34^ ^]^ Furthermore, adding CeO_2_ to a PdO/Al_2_O_3_ catalyst decreased the impact of water and SO_2_ poisons.^[^ ^120^, ^121^, ^122^ ^]^

However, the effect of CeO_2_ varies in different studies based on the catalyst preparation methods, which affect the particle size and Pd chemical state.^[^ ^123^ ^]^ Recently, Chen et al. found that small PdO crystallites were more prone to produce a large amount of Pd^0^ active sites during the reaction process, resulting in better catalytic activity in CH_4_ at low‐temperature. The calcination from 550 to 950 °C led to Pd particles ranging from 1.9 to 8.5 nm. The optimal catalytic properties were obtained on samples calcined at 550 °C with T_50_ of 420 °C, corresponding to the generation of small metallic Pd particles, which might be due to the stronger Pd–Ce interaction developed by 1.9 nm Pd particles. On the other hand, the catalyst calcined at 950 °C (8.5 nm) showed the highest T_50_ of 495 °C. The high calcination temperature deteriorated the Pd–Ce interface resulting in larger Pd particles accompanied by higher activation barriers.^[^ ^124^ ^]^

**Figure 6:**
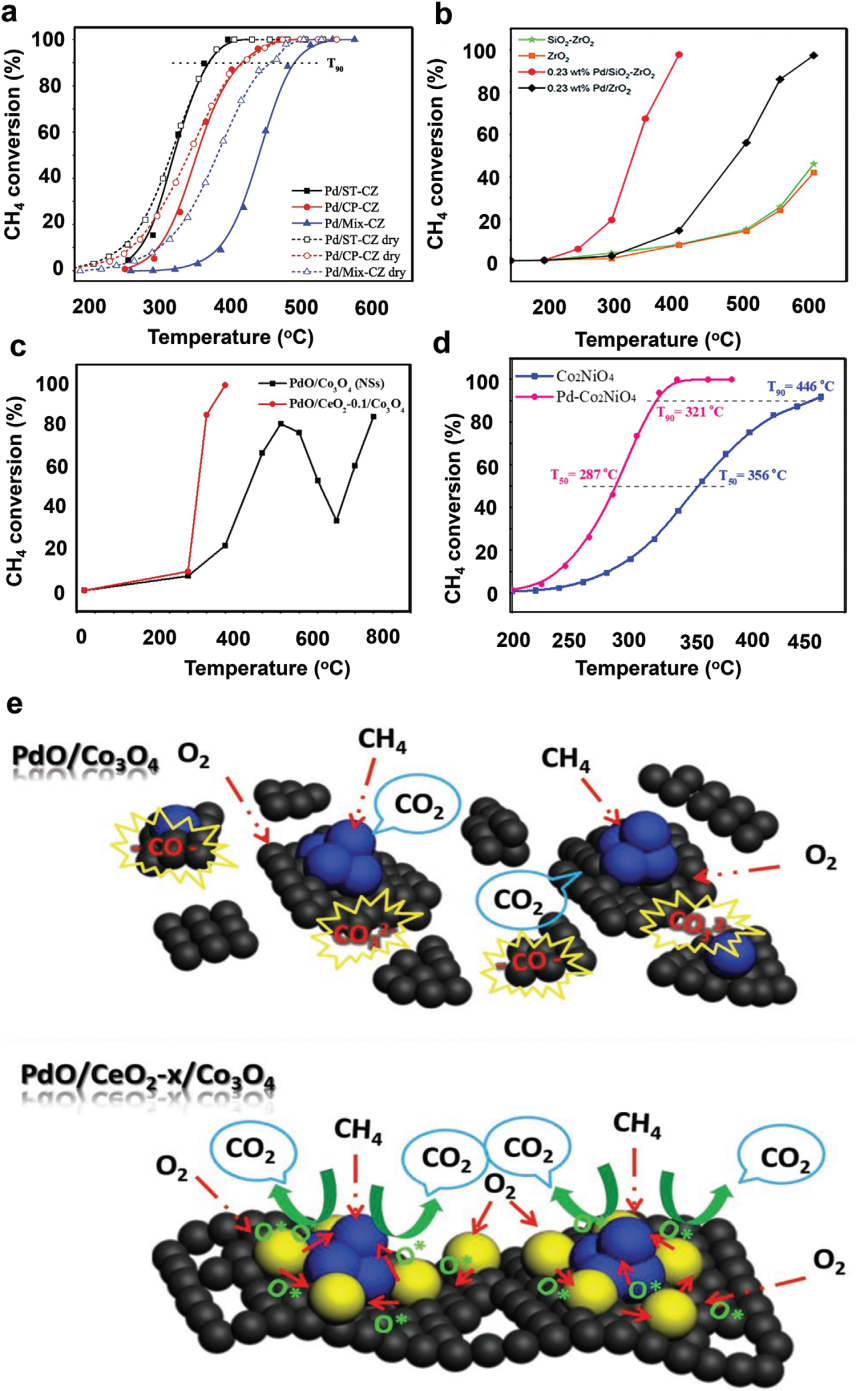
CH_4_ conversion as a function of temperature over a) Pd/CZ catalysts. Reproduced with permission.^[^ ^45^ ^]^ Copyright 2021, Elsevier. b) 0.23 wt% Pd/SiO_2_–ZrO_2_ catalyst and the 0.23 wt% Pd/ZrO_2_ catalyst. Reproduced with permission.^[^ ^111^ ^]^ Copyright 2017, Royal Society of Chemistry. c) PdO/Co_3_O_4_ before and after coating CeO_2_. Reproduced with permission.^[^ ^132^ ^]^ Copyright 2019, Wiley‐VCH. d) CH_4_ conversion over Co_2_NiO_4_ and Pd–Co_2_NiO_4_ catalysts. Reproduced with permission.^[^ ^134^ ^]^ Copyright 2021, Elsevier. e) Reaction mechanism of PdO/Co_3_O_4_ and PdO/CeO_2_‐*x*/Co_3_O_4_ toward catalytic CH_4_ combustion. Reproduced with permission.^[^ ^132^ ^]^ Copyright 2019, Wiley‐VCH.

It was reported that the high oxygen storage capacity of ceria–zirconia (CZ) mixture oxides could speed up the redox process of noble metal oxides deposited on the CZ surface to enhance the oxidation reaction during the combustion of CH_4_.^[^ ^45^, ^114^ ^]^ Ding et al. synthesized CZ mixed oxides with various morphologies using hydrothermal (ST‐CZ), coprecipitation (CP‐CZ), and physical mixing (Mix‐CZ) techniques. In addition, ST‐CZ revealed cubic and tetragonal phases structure of CZ solid solutions, while CP‐CZ demonstrated pure tetragonal‐phase solid solution and Mix‐CZ showed a mixture of CeO_2_ and ZrO_2_. It was realized that the unique structure of the ST‐CZ support increased the amount of Ce^3+^, oxygen species on the catalyst surface, and oxygen storage capacity. In addition, after the loading of Pd, the Pd/ST‐CZ catalysts showed smaller PdO*_x_* particle sizes and a high ratio of Pd^2+^/Pd on the catalyst surface, which is critical catalytic activity toward CH_4_ oxidation. Thus, the Pd/ST‐CZ catalyst demonstrates high catalytic performance for CH_4_ combustion of T_90_ at 352 °C while T_90_ for Pd/CP‐CZ and Pd/Mix‐CZ were 398 and 460 °C, respectively, as shown in **Figure** **6**a.^[^ ^45^ ^]^ Lin et al. successively reduced the activation temperature of CH_4_ through the calcination of CeO_2_–ZrO_2_–Al_2_O_3_ support at a high temperature which triggered the phase transition of alumina (from *γ*‐to *α*‐) and developed a solid solution of CeO_2_–ZrO_2_ (CZ). Because of the weak interaction between *α*‐Al_2_O_3_ and PdO, the reducibility of CeO_2_–ZrO_2_–Al_2_O_3_ composite oxides support (Pd/CZA) was improved. In addition, the increased oxygen mobility caused by the well‐crystallized CZ phase assisted in the reoxidation of Pd to PdO, resulting in plenty of surface‐active Pd^2+^ species. The surface property of the catalyst synthesized with CZA support with calcination at 1300 °C manifested excellent performance at low‐temperature a (T_90_ at 430 °C), excellent stability, and significantly improved water tolerance (T_90_ at 390 °C) toward CH_4_ combustion when combined with the hydrophobicity of *α*‐Al_2_O_3_.^[^ ^125^ ^]^

Due to their ability to modify the catalyst structure, different metals were introduced into the CZ mixed oxides as promoters. Recently, Zheng's team reported multicomponent Pd/5CZA‐yM catalysts using Mg‐promoter. Compared with Pd/Al_2_O_3_ (T_99_ = 485 °C), the results showed a higher initial catalytic activity for Pd/5CZA‐0 M (T_99_ = 455 °C), displaying the beneficial effect of the CZ component. Furthermore, the Mg‐doped samples had higher catalytic activity than the unmodified ones, with CH_4_ oxidation activity that varied with Mg amount. Pd/5CZA‐5 M (5 wt% Mg) exhibited superior catalytic performance and durability for CH_4_ oxidation with a T_99_ of 400 °C, in which 55 °C relatively decreased to Pd/5CZA‐0 M. Excessive doping, on the other hand, resulted in significantly decreased catalytic activity for Pd/5CZA‐7 M. The incorporation of Mg forms a MgAl_2_O_4_ spinel phase with alumina and causes Ce^4+^ to convert into Ce^3+^, giving rise to a lot of oxygen vacancies and more oxygen mobility as well as improved reducibility, which had a significant effect on redox properties, particle size, and chemical state, of active Pd species.^[^ ^65^ ^]^ The effect of Ca on the catalytic property of Pd/Ce–Zr/Al_2_O_3_ catalyst was also examined. According to the catalytic activity test, adding Ca to Pd/Ce–Zr/Al_2_O_3_ significantly improved its low‐temperature activity and thermal stability in CH_4_ combustion, generating well‐dispersed PdO particles on support as well as improving the reduction/reoxidation properties. In addition, Huang et al. proved that in situ addition of CaO can capture water produced during CH_4_ combustion and enhance Pd/CeO_2_ catalyst by maintaining the active PdO phase.^[^ ^126^ ^]^

In addition, SiO_2_, as a relatively inert oxide, could lead to a significant reaction temperature reduction by hindering water/hydroxyl chemical adsorption over active centers via interfacial effects.^[^ ^127^, ^128^, ^129^ ^]^ Furthermore, core–shell structures were proposed to maintain Pd nanoparticles' size and enhance metal–support interactions.^[^ ^130^ ^]^ Thus, Li et al. proposed a Pd catalyst based on SiO_2_‐modified cobalt–nickel mixed oxide; the results showed that this modification method improved the synergistic interaction of the deposited Pd phase with the reactive cobalt–nickel oxide, resulting in a significant improvement in CH_4_ combustion performance. At 323 °C, 90% CH_4_ conversion was achieved over 0.5Pd/M‐Co_1_Ni_4_, 62 °C lower than the untreated counterpart 0.5Pd/Co_1_Ni_4_. The addition of SiO_2_ to the Co_1_Ni_4_ support and Pd entities effectively modified their electronic structures, improved their low‐temperature redox capacity, and increased the supply of oxygen species throughout the reaction process. However, the results suggested that the amorphous SiO_2_ could be poor in hydrothermal stability.^[^ ^131^ ^]^ Compared to Pd supported on CeO_2_ and ZrO_2_, several oxide supports (Zr:Ce:Si) at varied ratios were combined; the results revealed that Pd/n‐SiO_2_ was the most active catalyst.^[^ ^111^ ^]^ Typically, the 0.23 wt% Pd/ZrO_2_ sample reached complete CH_4_ conversion at 600 °C, while 0.23 wt% Pd/SiO_2_–ZrO_2_ reached it at 400 °C, as shown in Figure 6b. The performance of Pd/SiO_2_–ZrO could be associated with the transfer of hydroxyl groups to the silica, which minimized the generation of inactive Pd(OH)*_x_* species and exposed more active sites to boost catalytic activity, as shown by the DRIFTS investigation.^[^ ^37^, ^111^ ^]^ In addition, the highly active Pd catalyst carried on CeO_2_ modified with organosilanes increased surface Pd species dispersion and strengthened active surface‐adsorbed oxygen species.^[^ ^58^, ^99^ ^]^

Through a green water‐phase route, Li et al. synthesized ultrathin Co_3_O_4_ nanosheet‐supported PdO/CeO_2_ (PdO/CeO_2_‐0.1/Co_3_O_4_) that showed outstanding catalytic activity with 90% conversion at 350 °C, as shown in Figure 6c. The catalytic performance studies on CH_4_ revealed that CeO_2_ as a catalytic assistant significantly improved the catalytic efficiency of PdO/Co_3_O_4_ through the strong synergetic effects with Pd species and Co_3_O_4_ components. The reaction mechanism revealed that CeO_2_ is involved by storing many oxygen species (O*) that can further flow over Co_3_O_4_ to PdO adsorbs and oxidizes CH_4_ molecules (Figure 6e). The continual O* supply from CeO_2_ increases the oxidizability of PdO, which in turn promotes the catalytic activity of the PdO/CeO_2_‐0.1/Co_3_O_4_ for CH_4_ oxidation. In addition, the in situ FTIR analysis demonstrated that the intermediate products deposited on CeO_2_ are more readily transformed into CO_2_. Therefore, PdO/CeO_2_‐0.1/Co_3_O_4_ behaved well on CH_4_ complete oxidation, which is impossible with PdO/Co_3_O_4_.^[^ ^132^ ^]^ In addition, the effect of the synthesis procedures of Co‐promoted Pd/alumina was investigated using co‐impregnation and sequential impregnation methods. Adding Co had little effect on CH_4_ conversion at a loading of 0.5 wt%. When the metal loading was between 1 and 3 wt%, the addition of Co by co‐impregnation increased the catalytic activity compared to Pd/Al_2_O_3_. At the same time, the consecutive impregnation of Co reduced the CH_4_ conversion at the metal loading of 1 wt%. A significant promotion impact of Co was observed at lower temperatures.^[^ ^133^ ^]^

**Figure 7:**
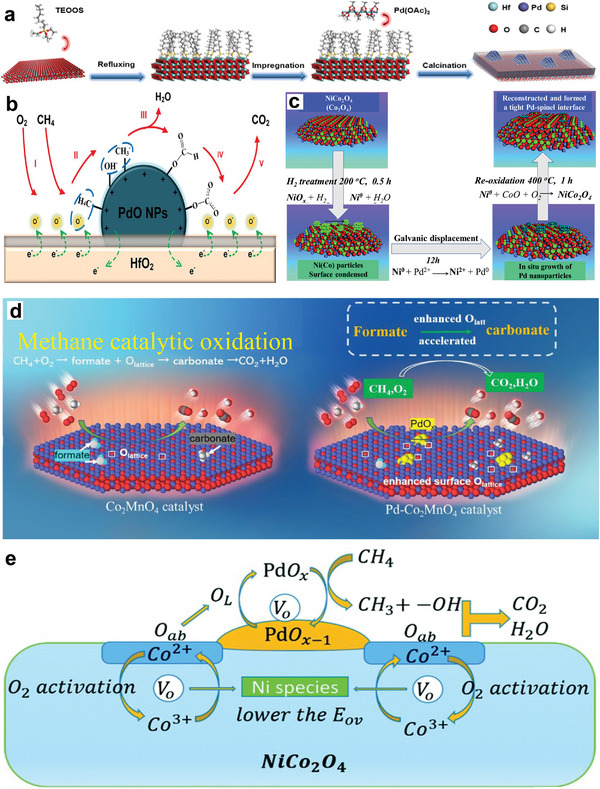
a) Illustration of the 1.0Pd/mod‐HfO_2_ synthesis process, b) proposed mechanism for the oxidation of CH_4_ over 1.0Pd/mod‐HfO_2_. Reproduced with permission.^[^ ^135^ ^]^ Copyright 2020, American Chemistry Society. c) Galvanic deposition procedure. Reproduced with permission.^[^ ^147^ ^]^ Copyright 2020, Royal Society of Chemistry. d) Proposed mechanism for CH_4_ oxidation over Co_2_MnO_4_ and Pd–Co_2_MnO_4_ catalysts. Reproduced with permission.^[^ ^145^ ^]^ Copyright 2020, Elsevier. e) The proposed reaction pathway over the GD‐Pd/ NiCo_2_O_4_ catalyst. Reproduced with permission.^[^ ^147^ ^]^ Copyright 2020, Royal Society of Chemistry.

Furthermore, Pd nanoparticles were coated on surface‐modified metal oxides, as shown in **Figure** **7**a (mod‐MO*_x_*, M = Ce, Hf, Ti, Zr, and Al), and used as lean CH_4_ oxidation catalysts. It was shown that when compared to their Pd/MO*_x_* counterparts, the modification of the surface of support materials enhanced the light‐off performance of Pd/mod‐CeO_2_, Pd/mod‐HfO_2_, and Pd/mod‐ZrO_2_ decreased purifying efficiency of Pd/mod‐TiO_2_ and 1.0Pd/mod‐Al_2_O_3_. 90% of CH_4_ was converted at 317 °C over the top‐performing Pd/mod‐HfO_2_ material, which was 120 °C lesser than the pristine Pd/HfO_2_ sample. Nevertheless, the addition of water vapor decreased the CH_4_ conversion from 61.3% to 20.4% at 300 °C.^[^ ^135^ ^]^ The above results showed that due to their steric confinement, the inserted silicon modifier materials could improve Pd nanoparticle dispersion, lower the activation temperature of CH_4_, and strengthen the formation of surface‐adsorbed oxygen species via electron transfer under dry conditions at low temperatures. The oxidation of CH_4_ over 1.0Pd/HfO_2_ and 1.0Pd/mod‐HfO_2_ followed the Langmuir–Hinshelwood (L–H) mechanism, as shown in Figure 7b. Pd catalysts supported on ZrO_2_–Al_2_O_3_ have been used to enhance CH_4_ abatement performance.^[^ ^136^ ^]^ However, further optimizing the activity of these conventionally prepared catalysts is a challenging issue, owing to problems in adjusting Pd dispersion and reducibility, both of which are critical in the complete oxidation of CH_4_. Thus, a new double‐solvent strategy for supported metal catalyst synthesis has piqued the interest of researchers since it has the potential to increase the metal dispersion of metal nanoparticles.^[^ ^137^ ^]^ Li et al. examined the development of a new Pd catalyst supported on Zr_0.5_Al_0.5_O_1.75_ using a simple double‐solvent procedure. The results indicated that using a double solvent during the synthesis of Zr_0.5_Al_0.5_O_1.75_ helped the dispersion of Pd nanoparticles, increasing the reducibility of the Pd catalyst and forming more active surface oxygen species. Hence, T_90_ (334 °C) for CH_4_ conversion obtained on Pd/ZA‐DS (double solvent) was 45 °C lower than that of Pd/ZA‐IM (impregnation).^[^ ^138^ ^]^

Previous studies have investigated alkaline earth metals as promotors due to their ability to donate electrons, increasing the electron density on the active metal surface.^[^ ^115^, ^139^, ^140^ ^]^ For example, the addition of Ba to Pd/Al_2_O_3_ increased CH_4_ oxidation activity in the wet condition; it was also discovered that the CH_4_ oxidation activity of the Ba promoted samples could be regenerated to a larger extent following water vapor exposure.^[^ ^141^ ^]^ Du et al. prepared Pd/Ba‐Al_2_O_3_ catalysts with various amounts of Ba as the promoter. Regarding CH_4_ conversion, intrinsic activity, and low CH_4_ activation temperature, Pd/Ba‐Al_2_O_3_ catalysts outperformed the reference Ba‐free Pd/Al_2_O_3_ catalyst. The Ba beneficial effect was derived from the location of Pd inside the porous channel as the addition of Ba can decrease the size of active metal Pd, increase the ratio of Pd^0^/(Pd^*δ*+^ + Pd^0^), enhance the reduction property and the activation capability toward O_2_. Notably, Pd/Ba‐Al_2_O_3_ showed alleviated H_2_O covering.^[^ ^142^ ^]^ Yang et al. reported a universal method for preparing irreducible oxide using Mg as a promotor. In oxygen‐rich circumstances, adding Mg reduced the rate of Pd oxidation, preserving the epitaxial structure and a suitable active phase of Pd–PdO*_x_* on MgAl_2_O_4_ and enhancing activity and stability.^[^ ^143^ ^]^

Binary oxides have also been proposed as promoters for the dry/wet synthesis of high‐activity and stable catalysts. For instance, Xu et al. introduced Co–Mn binary oxides to the Pd/Al_2_O_3_ system, leading to a high number of oxygen vacancies, which increased the surface concentrations of active Pd^2+^ and O_ads_ species while also allowing oxygen species to exchange. The resulting catalyst performed well with T_90_ of 100 °C lower than the catalyst without Co–Mn binary oxides. Furthermore, the Mn/Co synergy hastened the elimination of accumulated OH/H_2_O from active sites, restoring PdO and oxygen vacancies.^[^ ^144^ ^]^ Similarly, Xiong et al. utilized ultrathin mesoporous spinel catalysts as support to stabilize the PdO cluster by boosting the metal–support interactions at a scale near the atomic level.^[^ ^145^ ^]^ The as‐synthesized Pd‐embedded catalysts were found to have more active surface lattice oxygen, increased Co^3+^ in the surface layers, and excellent low‐temperature reducibility. After adding PdO species into Co_2_MnO_4_ structures, Pd–Co_2_MnO_4_ exhibits the highest catalytic performance with a T_90_ value of 324 °C. It evoked that the Pd–Co_2_MnO_4_ can endure water vapor and keeps outstanding activity to a certain extent as the CH_4_ conversion of the catalyst remained above 98% for 40 h at 340 °C in the 5 vol% water vapors. Based on the in situ DRIFTS results, the possible reaction pathway of the CH_4_ oxidation process on the catalyst's surface could be CH_4_ → CH_3_* → CH_3_O* → CH_2_O* → OCH_2_O* → OCHO* → OCHOO* →CO_2_ pathway as in Figure 7d. By contrast, when the PdO species were integrated into Co_2_AlO_4_, the reaction activity was equivalent to that of the original Co_2_AlO_4_ support, and Pd–Co_2_AlO_4_ did not increase CH_4_ oxidation activity. Furthermore, Pd–Co_2_MnO_4_ showed the lowest apparent activation energy value, confirming that CH_4_ combustion proceeds easier on the Pd–Co_2_MnO_4_ surface. The Pd–Co_2_MnO_4_ improvement might be associated with the strong interaction among the PdO and Co_2_MnO_4_ support improved oxygen release capacity, thus easing the C—H bond activation in CH_4_.^[^ ^145^ ^]^

Moreover, galvanic deposition was reported as a new method that uses ultralow metal content;^[^ ^146^ ^]^ it was used to prepare the bimetallic palladium catalysts for CH_4_ combustion, as shown in Figure 7c.^[^ ^147^ ^]^ Compared with Pd/Al_2_O_3_ catalyst, the catalyst prepared via the galvanic deposition method exhibits excellent CH_4_ combustion catalytic activity.^[^ ^146^, ^147^ ^]^ For instance, T_90_ of Pd/Ni‐Al_2_O_3_‐GD showed a decrease of 105 °C compared to Pd/Al_2_O_3_, which might be due to the strong interaction with the support as confirmed by XPS, O_2_‐TPD results; and the proposed mechanism was shown in Figure 7e.^[^ ^147^ ^]^ In addition, catalysts prepared by the galvanic method displays outstanding water tolerance below 300 °C.^[^ ^147^ ^]^ The activity of the catalyst prepared via incipient wetness impregnation (Pd‐Ni/Al_2_O_3_‐IW) was slightly lower than that of Pd/Al_2_O_3_‐IW.^[^ ^146^ ^]^ By introducing ultralow Ni content to Pd catalysts (12:1 molar ratio of Ni to Pd), Shen et al. reduced the temperature for complete CH_4_ oxidation in wet conditions (5% water) by 100° compared to monometallic Pd.^[^ ^148^ ^]^ Another study used a simple multispinel interface promotion approach to increase the performance and stability of Pd/Al_2_O_3_ for CH_4_ oxidation by sequentially growing a NiCo_2_O_4_/NiAl_2_O_4_ interface over *γ*‐Al_2_O_3_ support, which then served as a structural blueprint for the growth and dispersion of PdO nanoparticles to produce Pd/NiCo_2_O_4_/NiAl_2_O_4_/Al_2_O_3_. Because of the low CH_4_ activation energy, the reducible feature of NiCo_2_O_4_ and structural matching between Ni‐Co_2_O_4_/NiAl_2_O_4_ and PdO resulted in better efficiency during the catalytic oxidation of CH_4_. The T_90_ of Pd/NCO/NAO (345 °C) was lower than that of Pd/Al_2_O_3_ (365 °C), Pd/NAO (370 °C), and Pd/NCO (415 °C).^[^ ^149^ ^]^ Additionally, as shown in Figure 6d, PdO*_x_* species introduced into Co_2_NiO_4_ lattice modulates are primarily responsible for the increased CH_4_ oxidation activity of Pd–Co_2_NiO_4_ catalysts. This enhances the transfer of an electron between Ni and Co 3d‐O 2p hybrid orbital, e.g., orbital in Co_2_NiO_4_, which leads to the rapid migration and activation of lattice oxygen in Co_2_NiO_4_ support.^[^ ^134^ ^]^

It is worth emphasizing that the CH_4_ activation capacity was attributed to different parameters such as dispersion of palladium nanoparticles on the support,^[^ ^58^, ^135^, ^150^ ^]^ abundant oxygen vacancies and higher oxygen mobility,^[^ ^65^, ^151^ ^]^ excellent reducibility, and exposure of active PdO facets,^[^ ^40^ ^]^ the lattice matching based on the geometry of PdO and metal oxide support. Many reports claim that the Pd supported on the composite structure resulted in complete CH_4_ conversion, higher oxygen storage capacity, and mobility than Pd supported on single metal oxide. However, some reported composite catalysts activated CH_4_ at a higher temperature than catalysts supported on single metal oxide. For example, Pd/Co_3_O_4_ nanosheets, Pd/*γ*‐Al_2_O_3,_ and Pd/SiO_2_‐Acac synthesized through hydrothermal/Incipient wetness impregnation, Impregnation‐vortexing, and dry ball‐milling demonstrated T_90_ of 248, 275, and 288 °C, respectively, in the dry conditions.^[^ ^52^, ^84^, ^152^ ^]^ While Pd/NAAl_2_O_3_,^[^ ^33^ ^]^ Pd/SnO_2_,^[^ ^102^ ^]^ and Pd/ *α*‐Al_2_O_3_ demonstrated T_90_ of 350, 420, and 450 °C in the presence of water, respectively.

As shown in **Table** **2**, the Pd‐supported composite catalysts with the lowest temperature in the dry conditions are GD‐Pd–NiCo_2_O_4_,^[^ ^147^ ^]^ Pd/mod‐HfO_2_,^[^ ^135^ ^]^ Pd–Co_2_NiO_4_^[^ ^134^ ^]^ with T_90_ of 260, 317, and 321 °C, respectively. Surprisingly, few reports study the stability of Pd composite catalysts in water and SO_2_. Among them, Pd–Co_2_MnO_4_ and Pd/1.0Ni‐2Al in the presence of water showed T_90_ of 340 and 351 °C, respectively. It is also expected that Pd catalysts supported on composite metal oxides might reduce the catalyst cost. However, the composite metal oxides‐based catalyst has Pd loading comparable to that of single metal oxide, as shown in Tables 1 and 2. Therefore, cost evaluation studies at the industrial level are highly recommended^[^ ^153^, ^154^ ^]^ as the cost of the CH_4_ activation catalysts was never mentioned in all reviewed articles. In addition, there is still a need to understand the effect of Pd electronic configuration and morphology on CH_4_ activation^[^ ^155^ ^]^ as well as to reduce the amount of Pd loaded in the composite metal oxides‐based catalyst. To achieve this suggestion, reduction–deposition, precipitation–deposition, colloidal synthesis, and vapor deposition methods or single‐atom catalysts would be further explored.^[^ ^49^, ^75^, ^114^, ^156^, ^157^, ^158^ ^]^

**Table 2:** Summary of Pd‐based catalysts supported on composite metal oxide for CH_4_ activation

Catalyst	Metal loading [wt%]	Preparation method	Calc. temp. [°C]	Dispersion [%]	Particle size [nm]	CH_4_ conc. [%]	Catalyst amount [mg]	GHSV [mL g^−1^ h^−1^]	T_10_ [°C]	T_50_ [°C]	T_90_ [°C]	Refs.
Pd/*κ*‐CZ	0.95	Reduction–deposition	500	36.2	5.5	1	100	30 000	275	310	345	[114]
Pd/RAl_2_O_3_–CeO_2_	0.92		500	66.9	7.4	1	20	60 000	270	305	328	[217]
Pd/M‐Co_1_Ni_4_	0.5	Impregnation	500			1	20	60 000	243	292	323	[131]
Pd/CeO_2_NWs@SiO_2_	0.47		500			1	20	60 000	280	305	327	[150]
Pd/mod‐HfO_2_	1		500		17.84	1	20	60 000	236	288	317	[135]
PdO/ CeO_2_‐0.1/Co_3_O_4_		Water‐phase	400			0.5	30		250	300	350	[132]
Pd/ZA‐DS		Double solvent		18	62			50 000		297	334	[138]
Pd–Co_2_MnO_4_	0.99	In situ one‐step deposition/precipitation	500			1	100	30 000	243	291	324	[145]
Pd/1.0Ni–2Al	0.5	Incipient wetness	600			1	100		275	312	350	[218]
Pd–NiCo_2_O_4_	4.4	Galvanic deposition	400	64	1.41	1	100	24 000		230	260	[147]
Pd/NCO/NAO	0.2	Incipient wetness impregnation	500	10.9	6.8	1	200	30 000	240	300	345	[149]
Pd–Co_2_NiO_4_	0.98	In situ redox process	500	48.2	13.6	1	100	60 000	247	287	321	[134]
Pd, Pt, TiO_2_/H‐MOR	5	Wet impregnation	500		3.7	0.5	100	100 000	257	289	314	[167]
Pd@S‐1	0.6	One‐pot hydrothermal	550	66	2.3	1	100	50 000		323	380	[176]
Na‐FAU‐Pd (Geo 12)	5.65		550				200		226	237	250	[181]
Pd/H‐ZSM‐5‐Cs	0.78	Deposition–precipitation	400		3.2	1	200	30 000	235	279	304	[191]
Pd/H‐ZSM‐5‐Na	0.85	Deposition–precipitation	400		3.2	1	200	30 000	259	293	317	
Pd/NaLa‐ZSM‐5‐0.82	2	Incipient wetness impregnation	400		2.8		100	60 000	225	264	296	[196]
Pd–Y‐zeolite	3.04	Deposition by magnetron sputtering	550			10	200		250	285	300	[199]
Pd/3DOM LMAO	0.97	Gas‐bubble‐assistant adsorption	550		3–5	1.5	50	20 000	259	308	343	[219]

##### Pd Catalysts Supported on Zeolites

**Figure 8:**
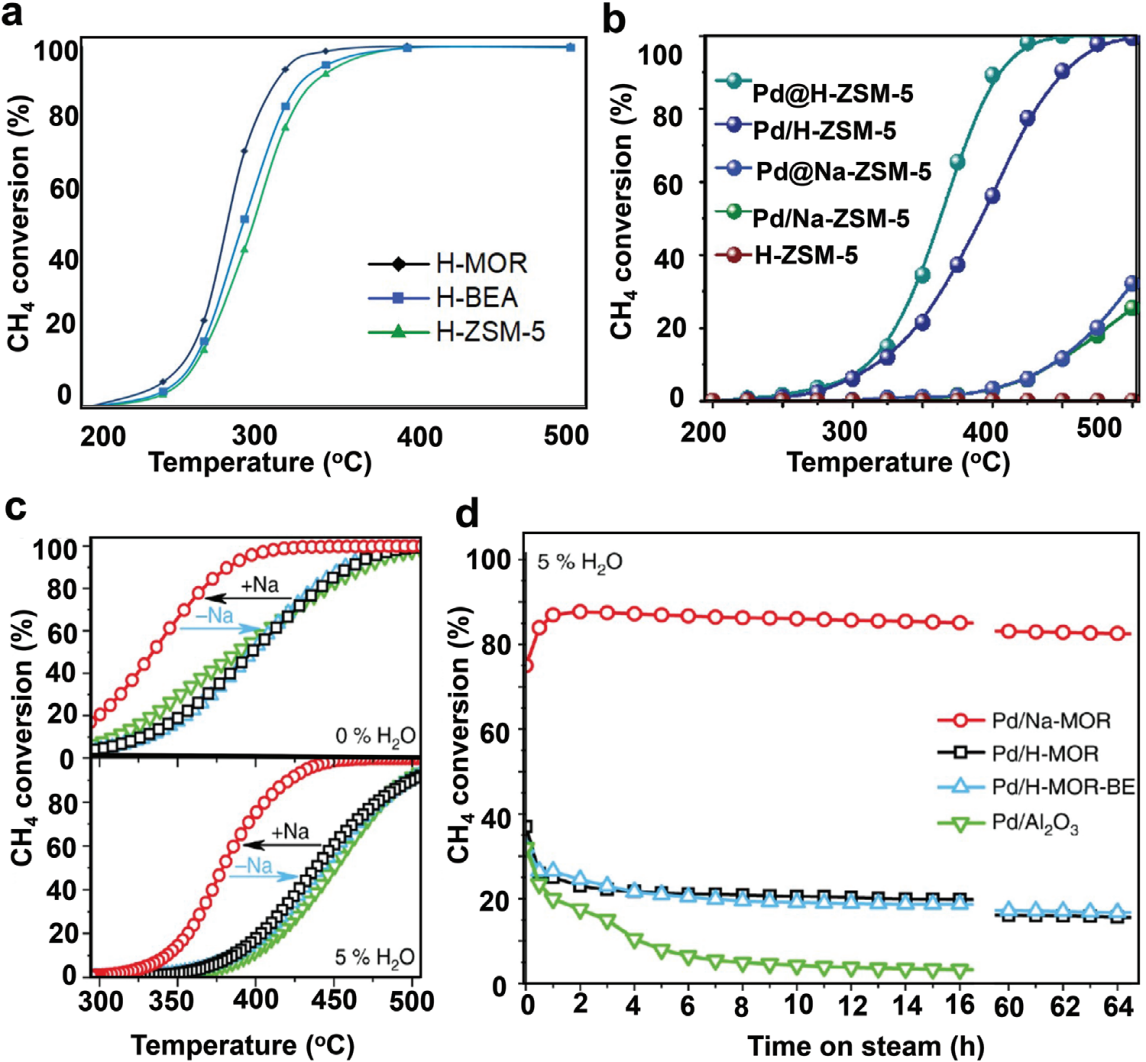
CH_4_ combustion over a) catalyst prepared on different zeolite supports. Reproduced with permission.^[^ ^167^ ^]^ Copyright 2020, Royal Society of Chemistry. b) H‐ZSM‐5 and Pd‐containing MFI zeolites. Reaction conditions: 1% CH_4_, 5% O_2_, He balance, GHSV = 60 000 h^−1^. Reproduced with permission.^[^ ^186^ ^]^ Copyright 2021, Elsevier. c) Pd/Al_2_O_3_ and various Pd/mordenite catalysts, and d) 65 h stability test without regeneration. Reproduced under the terms of the Creative Commons Attribution 4.0 International License.^[^ ^163^ ^]^ Copyright 2018, The Authors, published by Springer Nature.

Zeolites are an essential class of shape‐selective material with regular crystalline microporous aluminosilicates, controllable acidities, and high thermal and hydrothermal stabilities. Zeolites are also effective catalysts, although they cannot carry out necessary reactions independently. For this reason, zeolites are usually used as metal nanoparticles' support to add functionality.^[^ ^159^, ^160^ ^]^ Various techniques, like impregnation, deposition–precipitation, and colloidal synthesis, are generally used to incorporate metal‐based nanoparticles into the zeolitic structure. However, sophisticated methods are also used to prepare composite zeolites with a core–shell structure.^[^ ^161^ ^]^ In particular, the design and development of zeolite‐supported metallic catalysts, such as noble metal, with nanoparticles contained within the zeolite, have improved the application of zeolites for CH_4_ activation due to their advantages in shape‐selective reactions.^[^ ^162^, ^163^ ^]^ Li et al., for example, found that the temperatures at which 50–100% CH_4_ conversion was reached over Pd/ZSM‐5 were 70–80 °C lower than those at which Pd/Al_2_O_3_ conversion was achieved.^[^ ^164^ ^]^ Zeolites feature various intrapore spatial confinement and sieving properties that govern reaction intermediates and provide specific metallic species anchoring sites during CH_4_ activation, limiting aggregation and deactivation.^[^ ^165^ ^]^ There are currently 252 distinct zeolite framework topologies with varying channel systems, pore openings, and cavities;^[^ ^166^ ^]^ thus, selecting an appropriate zeolite topology for supporting metallic, active sites is critical to reducing the activation temperature of CH_4_. For instance, as shown in **Figure** **8**a, catalysts supported on the H‐ZSM‐5 possess lower activity than those supported on the H‐MOR and H‐BEA with similar SiO_2_:Al_2_O_3_ ratios.^[^ ^167^ ^]^

By analyzing the catalytic activity with numerous characterization techniques, Friberg et al. ascribe the level of CH_4_ activation to the Si/Al ratio (SAR), the type of zeolite framework, the reduction–oxidation behavior, and the form of Pd species.^[^ ^168^ ^]^ The results reveal that the type of zeolite framework and the SAR affect the kind of Pd species generated and the catalytic performance when using H‐beta (SAR = 511 and 40) and H‐SSZ‐13 (SAR = 43). It was indicated that the choice of zeolite carrier is critical in decreasing the generation of ion‐exchanged Pd^2+^ species and forming well‐dispersed Pd particles to generate a highly active CH_4_ oxidation catalyst. Additionally, the development of ion‐exchanged Pd^2+^ and large Pd particles can be avoided by using highly siliceous zeolites with minimal Brønsted acidic sites and small‐pore zeolites, which decrease Pd mobility. On the other hand, large pore size and low SAR make the catalyst more challenging to reduce and reoxidize, resulting in low CH_4_ conversion. In addition, Okumura et al. found similar results over Pd supported on zeolite H‐beta and H‐ZSM‐5 under dry and wet conditions. The generation of highly active PdO aggregates due to a lack of stabilizing acidic sites in the crystal lattice and the high hydrophobicity of zeolites with a high silica content, which results in less water adsorption, was linked to the high activity and resistance to water poisoning of Pd supported on siliceous zeolites.^[^ ^169^ ^]^ As a result, the inclusion of aluminum in the zeolite framework leads to a considerable water vapor affinity, provoking Pd/zeolite catalysts to deactivate during CH_4_ oxidation.^[^ ^170^ ^]^ Nonetheless, due to the increased hydrophobicity and stability of the zeolite support, high silicon‐to‐aluminum (Si/Al) ratios are suitable for activity and stability.^[^ ^171^ ^]^ In addition, Friberg et al. also confirmed that the SAR significantly affects catalytic activity in water vapor more than the zeolite framework type.^[^ ^162^ ^]^ More importantly, zeolites without aluminum, like silicalite‐1 and TS‐1, have better hydrophobic characteristics than zeolites with high silica content.^[^ ^172^, ^173^ ^]^ Pd particles confined in silicalite‐1 and synthesized core–shell catalysts displayed high activity at low temperatures, with a shielding effect on water and SO_2_ poisonous.^[^ ^174^, ^175^ ^]^ For instance, Wang et al. describe a one‐step process for producing Pd nanoparticles embedded in pure silicon silicalite‐1 (Pd@S‐1) with Pd loadings ranging from 0.3 to 1.6 wt%. The 0.6Pd@S‐1 showed the maximum catalytic performance with the complete CH_4_ conversion at 380 °C. On the other hand, the 0.6Pd/S‐1 and 0.6Pd/ZSM‐5 synthesized by the IWI approach had substantially higher T_100%_ of about 470 and 465 °C, respectively, under the same conditions. Furthermore, the E_a_ value over 0.6Pd@S‐1 was smaller than the others, suggesting that Pd sites inside S‐1 crystals had high intrinsic activity.^[^ ^176^ ^]^ Moreover, due to the hydrophobicity of the pure silica zeolite, the Pd@S‐1 was able to selectively prevent water vapor from diffusing into the Pd sites, resulting in outstanding water resistance.^[^ ^176^, ^177^ ^]^ In addition, Pd@S‐1 and Pd0.8Ni0.2@S‐1 showed a T_90_ of 385 and 412 °C, respectively, in a 5% water vapor condition.^[^ ^174^ ^]^

The enhanced role of adding TiO_2_ to the zeolite was explored, and the results demonstrated that adding TiO_2_ to Pd‐based catalysts for CH_4_ combustion improved catalyst performance.^[^ ^3^, ^178^ ^]^ Hosseiniamoli et al. synthesized a type of hydrophobic catalysts (Pd/TS‐1 and Pd/silicalite‐1) and found that Pd supported on TS‐1 (Pd/TS‐1) activity was higher than Pd/silicalite‐1, with a T_90_ temperature difference of 25 °C between the two catalysts. The Ti at the T sites of the TS‐1 framework is considered to anchor Pd particles and prevent sintering by causing migration and agglomeration of Pd particles.^[^ ^171^ ^]^ However, due to their high hydrophobicity and small ion‐exchange capacity, it is difficult to establish high Pd dispersion inside high silica zeolites due to Pd incorporation into the zeolite pores. Different efforts have been made to improve the dispersion of Pd through various routes, such as seed‐directed and assisted hydrothermal strategy and reduction–deposition, for simultaneously increasing the activity, stability, and water resistance of Pd/Beta at P/Beta low‐temperature.^[^ ^174^, ^179^ ^]^

On the other hand, low Si/Al ratios can achieve high dispersion even at high active phase loadings.^[^ ^180^ ^]^ A high number of acidic sites in zeolites with low SAR resulted in more excellent particle dispersion and monoatomic Pd^2+^ species, whereas highly siliceous zeolites produced only Pd particles of larger sizes. Furthermore, when compared to Pd/Al_2_O_3_, zeolite‐supported Pd was more susceptible to SO_2_.^[^ ^162^ ^]^ Khivantsev's team produced high loadings of atomically dispersed Pd in SSZ‐13 (Si/Al ≈ 6), SSZ‐39 (Si/Al ≈ 12) and applied them to CH_4_ combustion, and the results showed that it is active for complete CH_4_ oxidation at low temperature.^[^ ^38^ ^]^ In addition, by addressing the reasons for catalyst deactivation, Petrov et al. developed a highly active catalyst resistant to reaction‐induced steam sintering. The complete elimination of the zeolite's acid sites by sodium post‐exchange, as well as the simultaneous confinement of Pd nanoparticles within the high zeolite mobility of Pd nanoparticles and zeolite degradation challenges, were solved. In lean‐burn CH_4_ oxidation, the results showed that Pd/H‐MOR and a reference 1 wt% Pd/Al_2_O_3_ catalyst synthesized by wet impregnation have similar light‐off curves, providing T_50_ at 400 and 435 °C, respectively, in the dry and wet condition. Pd/Na‐MOR T_50_ values changed significantly to 340 and 375 °C, respectively, as shown in Figure 8c,d. The acidic Pd/H‐MOR‐BE was created by exchanging Pd/Na‐MOR with ammonium nitrate and calcining it. This allowed researchers to examine the potential effects of acidity removal on the system's ability to function and to rule out any other potential causes, such as changes in the pore structure and crystallinity of the support material. This catalyst's T_50_ values (400/440 °C) were comparable to those of Pd/H‐MOR; the similarities between Pd/H‐MOR and Pd/H‐MOR‐BE, as well as the superior performance of fully exchanged Pd/Na‐MOR, suggest that the zeolite acidity governs the system's catalytic behavior.^[^ ^163^ ^]^ Furthermore, Doyle et al. reported the Na^+^ forms of the catalysts to be more active than the H^+^ forms.^[^ ^181^ ^]^ The Na‐FAU and H‐FAU zeolites were loaded with Pd, and the Na‐FAU‐Pd zeolites had the maximum reactivity, with T_90_ values of 250 °C. Lower catalytic activity in the H^+^ form was associated with the inclusion of Pd and/or an additional calcination step to generate H^+^, which reduced the crystallinity and porosity of the catalyst.

By contrast, it was claimed that strong solid acids and Brønsted acidic sites might directly protolyze the C—H bond and activate short‐chain alkanes at low temperatures because of zeolite's limited redox capacity.^[^ ^182^ ^]^ Meanwhile, according to DFT and experimental analysis, the surface acid sites of H‐ZSM‐5 do not participate in the catalytic reaction, but they can effectively change the electronic and coordination structure of Pd species, giving the Pd/H‐ZSM‐5 catalysts excellent catalytic activity and long‐term stability for CH_4_ combustion at low temperatures.^[^ ^167^, ^183^ ^]^ Dai et al. obtained similar results when they employed ZSM‐5 zeolites (NaZSM‐5, HZSM‐5, and Silicalite‐1) of varying acidity as support for Pd catalysts for catalytic oxidation of CH_4_. The findings of the experiments revealed that the anchoring effect of Brønsted acid sites on Pd species was not directly noticed. However, the acid properties of the supports (Al^3+^ as Lewis acid sites, particularly coordinatively unsaturated Al^3+^) were critical for the preparation of Pd/ZSM‐5 catalysts with significant Pd dispersion.^[^ ^184^ ^]^

If adequately managed, oxidative treatment could result in isolated cation sites bounded by zeolites, intended to be effective catalysts in oxidation reactions. In addition, Zeolites can serve as functional centers, such as acid–base sites for cooperative catalysis and bounded cation sites for improving overall catalytic performance.^[^ ^185^ ^]^ Recently, Gao et al. encapsulated cationic Pd sites inside zeolite MFI using in situ hydrothermal routes accompanied by an oxidative procedure. Within the constrained environment of MFI zeolite, it is realized that the existence of Brønsted acid sites could boost CH_4_ activation over Pd sites. The parent H‐ZSM‐5 was entirely inactive for CH_4_ oxidation at <500 °C. Adding Pd species through in situ synthesis or wet impregnation effectively enhanced CH_4_ oxidation at <500 °C. The acidic H‐ZSM‐5 offered a superior carrier for active Pd species compared to the basic Na‐ZSM‐5. In addition, Pd@H‐ZSM‐5 and Pd/H‐ZSM‐5 showed considerably higher activity than their Pd@Na‐ZSM‐5 and Pd/Na‐ZSM‐5 counterparts. As shown in Figure 8b, the apparent activation energy value for Pd@HZSM5 was 70.7 kJ mol^−1^, significantly lower than that for Pd/HZSM5 (91.4 kJ mol^−1^) and Pd@NaZSM5 (112.4 kJ mol^−1^).^[^ ^186^ ^]^ It is evident that the existence of Brønsted acid sites assisted CH_4_ activation over Pd sites and lowered the energy barrier. The collaboration among Pd sites and adjacent Brønsted acid sites could be suggested for CH_4_ activation.^[^ ^34^, ^186^, ^187^ ^]^ However, excess water vapor induced the reversible deactivation of Pd@H‐ZSM‐5 due to the poisoning of Brønsted acid sites by water vapor.^[^ ^186^ ^]^ Meanwhile, previous research has demonstrated that the activity of Pd/Na‐zeolites is favorably related to the Na/Pd ratio. It was shown that in the case of mordenite with a Si/Al ratio of 51, the traditional ion exchange technique resulted in just 46% cation exchange.^[^ ^188^ ^]^ Another study revealed that dealuminated Pd/H‐MOR produces a highly active and stable catalyst that removes all residual acid sites when completely exchanged with sodium.^[^ ^163^ ^]^

It was also recognized that the alkali metal additions might function as an electronic or textural stimulant to alter the electron density and dispersion of noble metals, increasing the effectiveness of supported noble metal catalysts^[^ ^189^ ^]^ Furthermore, researchers compared the activity of Pd anchored on Na^+^ and H^+^ forms of zeolites to understand the role of cation in catalysis; some research indicated that Na^+^ in the zeolites could inhibit Pd catalyst activity,^[^ ^180^ ^]^ whereas others suggested that Na^+^ in the zeolites had a positive effect on activity and stability.^[^ ^163^, ^181^, ^190^ ^]^

Therefore, Luo et al. used various alkali metal hydroxide precipitants to explore their impact on the efficiency of catalysts for CH_4_ oxidation. The presence of alkali metals decreased the T_90_ considerably from 379 °C (Pd/H‐ZSM‐5) to 304 °C (Pd/H‐ZSM‐5‐Cs). Moreover, the apparent activation energies for CH_4_ oxidation over the modified catalyst (Pd/H‐ZSM‐5‐Me) were much lesser than the unmodified catalyst. The TEM images of Pd/H‐ZSM‐5 catalysts suggest that the alkali metal‐free Pd/H‐ZSM‐5 have an average Pd particle size of 10.3 nm, while the dispersion of Pd/H‐ZSM‐5‐Me significantly improved with the average Pd particle size ranging from 2.9 to 3.5 nm. It indicates that Pd particle size might be reduced, and alkali metal hydroxide precipitants could increase Pd dispersion on the H‐ZSM‐5 support. Additionally, the alkali metal cations can act as an electron‐donating component, endowing the Pd species with a distinct electronic property that can improve the activation of the C—H bond of CH_4_ molecules. The results clarified the function of alkali metals in the catalytic activity of Pd/H‐ZSM‐5‐Me, as well as a simple but effective procedure for increasing the efficiency of Pd‐based catalysts in lean CH_4_ combustion.^[^ ^191^ ^]^

Furthermore, several articles compared Na and proton forms of Pd/zeolite catalysts for total CH_4_ oxidation. Maeda et al. studied Pd/H‐MOR and Pd/Na‐MOR with different Si/Al ratios and found a high Si/Al ratio (260), and the existence of Na^+^ in the zeolite has an adverse effect on both activity and stability.^[^ ^188^ ^]^ Furthermore, a recent innovative synthesis technique that provides CH_4_ oxidation catalysts with exceptional activity and stability clarifies the contradiction in the literature about the involvement of Na^+^ and expands the understanding of the stability characteristics of Pd/Na‐zeolites.^[^ ^190^ ^]^ Pd in zeolites can be found as atomically dispersed ionic Pd or as PdO nanoclusters,^[^ ^192^ ^]^ with PdO nanoclusters being more effective than isolated ionic Pd in the CH_4_ oxidation process.^[^ ^193^ ^]^ It is challenging to reduce the amount of Pd in Pd/zeolite catalysts and keep the PdO active sites because the Pd loading determines the state of Pd inside the zeolite framework.^[^ ^194^, ^195^ ^]^ Therefore, it was proposed to increase the low‐temperature CH_4_ oxidation performance by introducing nonprecious metals into Pd/zeolite catalysts. Shi et al. added several metals to Pd/HZSM‐5. Within all the investigated catalysts, Pd‐Zr/HZSM‐5 and Pd‐Ce/HZSM‐5 could achieve 100% CH_4_ conversion at 55 and 40 °C less than Pd/HZSM‐5, respectively. Incorporating Zr or Ce led to better surface oxygen species reactivity by decreasing the PdO bond.^[^ ^196^ ^]^

**Figure 9:**
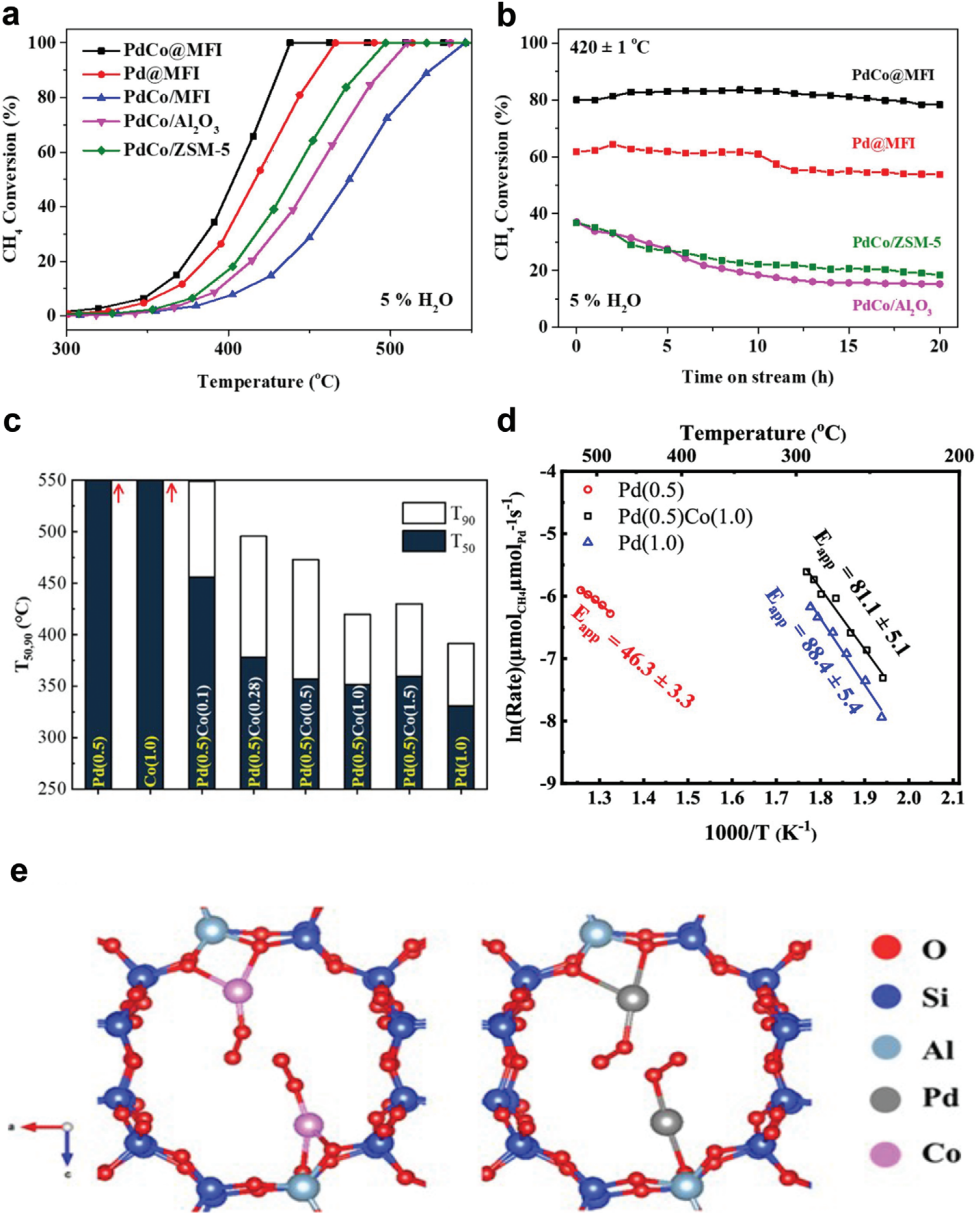
a) The activity and b) stability test of various catalysts in wet condition. Reproduced with permission.^[^ ^197^ ^]^ Copyright 2021, Elsevier. c) T_50_ and T_90_ of CH_4_ combustion over Pd(0.5)Co(*x*)/BEA, Co(1.0)/ BEA, and Pd(1.0)/BEA catalysts. d) Arrhenius plot and apparent activation energies for CH_4_ combustion over Pd(0.5)Co(*x*)/BEA and Pd(1.0)/BEA catalysts. e) Structures of Co/BEA and Pd/BEA. Reproduced with permission.^[^ ^198^ ^]^ Copyright 2021, American Chemistry.

Tang et al. introduced Co species into the channels of MFI zeolite to improve the activation of surface oxygen species and modify the electronic state of Pd species, as illustrated in **Figure** **9**a. This increased the catalytic activity with low temperature for CH_4_ complete conversion in the presence of steam. The PdCo particle size and catalytic performance of PdCo@MFI stayed stable after 20 h at 420 °C in the presence of steam (Figure 9b), substantially more stable than the PdCo/ZSM‐5 and PdCo/Al_2_O_3_ reference catalysts, which decreased 19% and 22%, respectively.^[^ ^197^ ^]^ Incorporating cobalt (Co) into 0.5 wt% Pd/BEA dramatically increased CH_4_ oxidation performance over its monometallic counterpart. The CH_4_ oxidation activity improved steadily as Co loading raised from 0 to 1 wt%, with T_50_ dropping above 500 °C for Pd (0.5)/BEA to 352 °C for Pd(0.5)Co(1.0)/BEA (Figure 9c). Furthermore, at 250 °C, the CH_4_ oxidation reaction rate of Pd(0.5)Co(1.0)/BEA was 77% higher than that of Pd(1.0)/BEA (Figure 9d). The production of highly active PdO rather than less active ionic Pd was attributed to the promotion effect of Co incorporation. Stability studies over Pd(0.5)Co(1.0)/BEA revealed comparable CH_4_ oxidation activity and slightly enhanced H_2_O resistance to Pd(1.0)/BEA. In addition, DFT calculations prove Co to be more stable than Pd at the ion‐exchange sites (Al sites) of BEA zeolites (Figure 9e). Pd has access to fewer ion‐exchange sites because Co is more stable there than Pd, which makes Pd less ionic but more likely to generate PdO nanoclusters over bimetallic PdCo/BEA catalysts.^[^ ^198^ ^]^ As shown in Table 2, Na‐FAU‐Pd activated CH_4_ at the lowest temperature among all Pd supported on zeolite, followed by Pd/NaLa‐ZSM‐5‐0.8, Pd‐ Y‐zeolite, Pd/H‐ZSM‐5‐Cs with T_90_ of 250, 296, 300, and 304 °C, respectively.^[^ ^181^, ^191^, ^196^, ^199^ ^]^ Notably, Pd introduced into zeolites through deposition methods provided promising results. However, there is still a need to homogeneously introduce Pd precursors into zeolite micropores. Hence, the vapor deposition method might be helpful to control the sizes and chemical compositions of active species within the zeolite microporous environment. In addition, a fundamental understanding of nucleation and crystallization in zeolites and mechanistic insight on nanoparticle growth might be helpful, as the dynamics happening at the cluster scale could be the missing piece of this puzzle to design the catalysts of our choice.

##### Pd Catalysts Supported on Other Supports: Pd Catalysts Supported on Hydroxyapatite (HAP)

**Figure 10:**
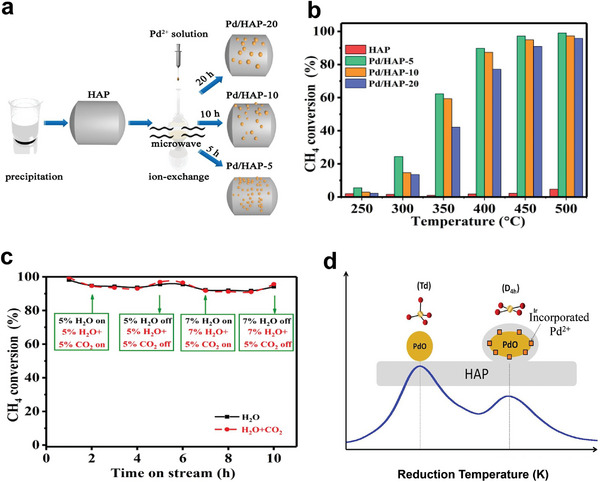
a) The preparation process for Pd/HAP catalysts using an ion‐exchange period of 5, 10, and 20 h. b) CH_4_ conversion over the different Pd/HAP catalysts, c) resistance of Pd/HAP‐5 toward 5 vol% CO_2_ and 5 vol% H_2_O or 7 vol% H_2_O in CH_4_ combustion at 450 °C. Reproduced with permission.^[^ ^202^ ^]^ Copyright 2020, Elsevier. d) Proposed scheme for Pd species distribution. Reproduced with permission.^[^ ^203^ ^]^ Copyright 2020, Elsevier.

It has been disclosed that incorporating phosphorus into Al_2_O_3_ could improve the H_2_O tolerance for Pd/Al_2_O_3_ due to P=O interaction with hydroxyl groups for P—OH generation.^[^ ^55^ ^]^ Highly stable HAP (Ca_10_(PO_4_)_6_OH_2_) is a form of apatite with a flexible framework and outstanding ion‐exchange capacity; therefore, it is frequently utilized as a carrier in heterogeneous catalysis.^[^ ^200^, ^201^ ^]^ The high concentration of PO_4_^3−^ groups may protect active metals from sintering and H_2_O poisoning.^[^ ^55^ ^]^ Given the high amount of PO_4_^3−^ in HAP, Zhang et al. prepared Pd/HAP catalysts by combining the ion‐exchange method and single‐mode microwave technology, as shown in **Figure** **10**a. It was discovered that ion‐exchange time influences the dispersion and size of Pd particles on HAP (Figure 10b). For a 5 h ion‐exchange catalyst (Pd/HAP‐5), T_90_ CH_4_ conversion was 400 °C. However, when the time reached 20 h, there was a decline in catalytic performance. The catalytic performance of Pd/HAP‐5 can be associated with high Pd dispersion, the presence of oxygen species, catalyst reducibility, and strong CH_4_ adsorption. Moreover, Pd/HAP shows improvement durability in the presence of 7 vol% H_2_O and CO_2_ (Figure 10c).^[^ ^202^ ^]^ Based on the DRIFTS and XPS analyses, CH_4_ oxidation over Pd/HAP‐5 followed the MvK and L–H paths. The adsorbed oxygen species accelerated the dissociation of CH_4_ to *CH_3_ and H*, then H* combined with O^−^ species to generate OH^−^ while *CH_3_ interacts with PdO to produce —OCH_3_. The —OCH_3_ intermediate goes through dehydrogenation to make HCOO—, which oxidizes to —OCOO—, resulting in the production of CO_2_. Meanwhile, as the adsorbed oxygen species also react with adsorbed CH_4_, oxidation might simultaneously proceed with the L–H mechanism to some extent.

It is widely known that Pd catalysts' thermal treatment plays a crucial role in establishing the active phase's oxidation state and how it interacts with the support. Thus, Tang et al. reported that the Pd/HAP system exhibits oxidative strong metal–support interactions (SMSIs) at high temperatures due to the loss of surface OH groups.^[^ ^17^ ^]^ Boukha et al. studied the impact of calcination in the 773–1073 K temperatures range on the properties of Pd/HAP samples; in their study, four HAP carried Pd samples, with a Pd loading near 0.5%, acquired via calcination at 773, 873, 973, or 1073 K were studied. It was revealed that when the calcination temperature rises, the support dehydrates, and the structural evolution of Pd^2+^‐containing species shifts from tetrahedral (Td) to square planar geometry (D4h). Furthermore, this significantly improves the metal–support interactions. For example, Pd particles were enclosed by a thin support layer at the highest temperature (1073 K). Consequently, SMSI has been identified in two different reducible species. Increased Pd–HAP interaction strength appears to enlarge the HAP lattice, shift the Pd^2+^ coordination geometry from Td to D4h (Figure 10d), enhance PdO reduction, and inhibit CO chemisorption. These properties compensate for the Pd active phase's poor textural properties and improve the performance and stability of the CH_4_ oxidation reaction.^[^ ^203^ ^]^

##### Pd Catalysts Supported on Halloysite Nanotubes (HNTs)

**Figure 11:**
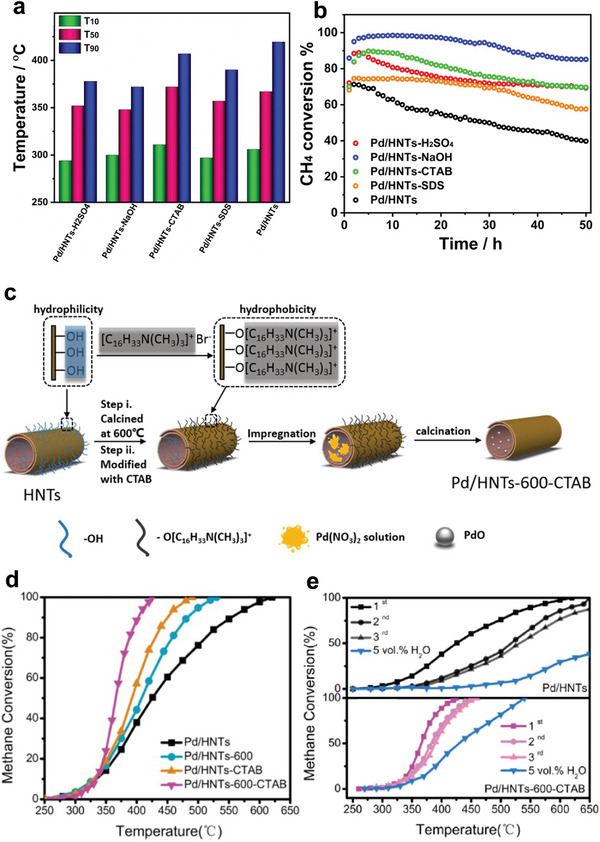
a) Performance indicators and b) stability measurement at 350 °C for Pd‐supported on HNTs catalysts. Reproduced with permission.^[^ ^206^ ^]^ Copyright 2021, Elsevier. c) Synthesis of Pd/HNTs‐600‐CTAB catalyst with Pd nanoparticles inside the tube, d) CH_4_ combustion over different catalysts, and e) stability tests with and without H_2_O. Reproduced with permission.^[^ ^207^ ^]^ Copyright 2020, American Chemistry Society.

HNTs (Al_2_(OH)_4_Si_2_O_5_⋅*n*H_2_O) are hollowed structural aluminosilicates that occur naturally. HNTs have a high surface area and a meso/macroporous structure, which allows for the effective dispersion of nanoparticles for various catalytic applications.^[^ ^204^ ^]^ In addition, they feature an inner layer consisting of aluminol groups (Al‐OH) and an exterior layer composed of siloxane groups (Si–O–Si), which provide negative and positive charges at the outer and inner surfaces in an aqueous solution throughout a wide pH range.^[^ ^205^ ^]^ Ahmed et al. synthesize Pd@HNTs using a simple mild reduction method of Pd‐precursor on HNTs modified by H_2_SO_4_ (HNTs‐H_2_SO_4_), NaOH (HNTs‐NaOH), cetyltrimethylammonium bromide (HNTs‐CTAB), and sodium dodecyl sulfate (HNTs‐SDS) treatment chemical treatments and investigate the effect of these treatments on catalysts properties and activity. As demonstrated in **Figure** **11**a,b, all catalysts based on chemically modified HNTs outperformed Pd‐supported on pristine HNTs in terms of catalytic performance for CH_4_ combustion. For instance, Pd/HNTs‐NaOH achieved 90% conversion at 373 °C whereas Pd/HNTs‐H_2_SO_4_, Pd/HNTs‐SDS, Pd/HNTs‐CTAB, and Pd/HNTs demonstrated similar conversion at 377, 390, 377, and 420 °C, respectively. Pd/HNTs‐NaOH activity has been attributed to the localization of Pd nanoparticles at the inner surface, which improves the catalyst–support interaction, multiplies the number of active surface sites, and serves as a barrier to sintering at high temperatures.^[^ ^206^ ^]^

Furthermore, the outer surface of the halloysite became hydrophobic after thermal treatment and modification with cetyltrimethylammonium bromide resulting in the selective Pd loading on the hydrophilic inner surface of the halloysite (Figure 11c). The tailored catalyst displayed a notably increased activity, with the T_99_ lowering from 620 to 425 °C, as shown in Figure 11d, thanks to regular dispersed Pd particles (≈2 nm), a reasonable ratio of Pd^2+^/Pd^4+^, good reducibility, and optimum surface acidity. More interestingly, the stabilized Pd species were resistant to sintering, and the hydroxyl groups on the surface were reduced, weakening the water interaction, and resulting in outstanding long‐term/cyclic stability and water resistance for CH_4_ combustion (Figure 11e).^[^ ^207^ ^]^ Feng et al. used a photo‐assisted thermal catalytic route to generate PdO/Mn_3_O_4_/CeO_2_ (PMC) nanocomposites by initiating aqueous autoredox reactions between reductive Ce(OH)_3_ and oxidative MnO_4_/Pd^2+^ ions. To form the final 1D HNTs‐supported PMCs, PMCs could spontaneously self‐assemble into compact encapsulation on the surface of HNTs (HPMC). HPMC could show good performance on photo‐assisted thermal catalytic CH_4_ oxidation with T_50_ lowered to 225 °C under visible light irradiation while it was 320 °C without visible light due to the strong synergistic effects between the components of PdO, Mn_3_O_4_, and CeO_2_.^[^ ^30^ ^]^

#### 2.1.2 Pt‐Based Catalysts

Noble metals like platinum exhibit good catalytic activity and stability due to their high resistance to carbon deposition and tolerance to sulfur poisons.^[^ ^208^, ^209^ ^]^ For instance, Torralba et al. presented a 1% Pt nanoparticle supported on ZrO_2_; the catalyst was highly resistant to sulfur deactivation, with complete conversion of CH_4_ at 485 °C. Its performance at low temperatures was associated with the higher electric dipole moment of SO_2_ molecules than that of CH_4_, which polarize more easily and get adsorbed on dipolar sites of the catalyst surface at a higher rate. Most of the polarized SO_2_ molecules are adsorbed on Pt^2+^–Zr^4+^ dipolar sites, while the CH_4_ molecules are adsorbed on Pt^2+^–Pt^4+^ dipolar sites. Thus, the SO_2_ oxidation and CH_4_ oxidation reactions at the catalyst surface occur independently, without affecting each other.^[^ ^210^ ^]^ In general, the oxidation process of CH_4_ is seen to follow the Mars–van Krevelen reduction–oxidation mechanism.^[^ ^211^ ^]^

Although the high activity of Pt‐supported catalysts has been linked to various parameters, such as Pt particle size in specific crystallographic planes, the findings point to a possible activation of the C—H bond over the Pt surface having Pt^0^–Pt^x+^ sites, which could polarize the CH_4_ molecule, lowering the C—H bond energy and facilitating its cleavage.^[^ ^212^ ^]^ Corro et al. recently investigated the possibility of preparing a dipolar catalytic site to increase CH_4_ dissociation. The effect of the support on the formation of stable and active Pt^0^–Pt^x+^ polar sites on the catalytic activity of Pt‐supported Cr_2_O_3_ in complete oxidation of CH_4_ was examined using the valence state of Pt‐supported on n‐type semiconductor Cr_2_O_3_. The results demonstrated that the conversion of CH_4_ was substantially high for the 1% Pt/Cr_2_O_3_ composite catalyst at low temperatures. Furthermore, the incorporation of 1% Pt in Cr_2_O_3_ supports the creation of highly stable Pt^0^–Pt^4+^ dipoles at the Pt–support interface, which are incredibly active catalytic sites for the dissociation and oxidation of CH_4_ at 440 °C. These findings show that the electronic state of Pt, as well as its electronic interactions with the Cr_2_O_3_ support surface, are critical components in the CH_4_ oxidation process.^[^ ^213^ ^]^ Additionally, the pre‐treatments of Pt/Al_2_O_3_ catalysts with Ar/CH_4_ mixture were also found to reduce the CH_4_ combustion reaction temperature from 518 to 450 °C.^[^ ^214^ ^]^ Using Pt/Y_2_O_3_ and a mixture of NO and O_2_ as the oxidant, Vargheese et al. activated CH_4_ at 375 °C. The results showed that NO was reduced to N_2_; however, surface nitrate species were formed, considered key intermediate species for selective oxidation.^[^ ^215^ ^]^ At the same time, Kye et al. used CO as a promotor to improve the catalytic activity of Pt/CeO_2_ over CH_4_ activation and its conversion into methanol with a selectivity of over 95% at 300 °C. The performance of Pt/CeO_2_ was attributed to active lattice oxygen produced at the Pt/CeO_2_ interface, which enables selective reaction routes for converting CH_4_ to methanol.^[^ ^216^ ^]^

#### 2.1.3 Pt–Pd Based Catalysts

**Figure 12:**
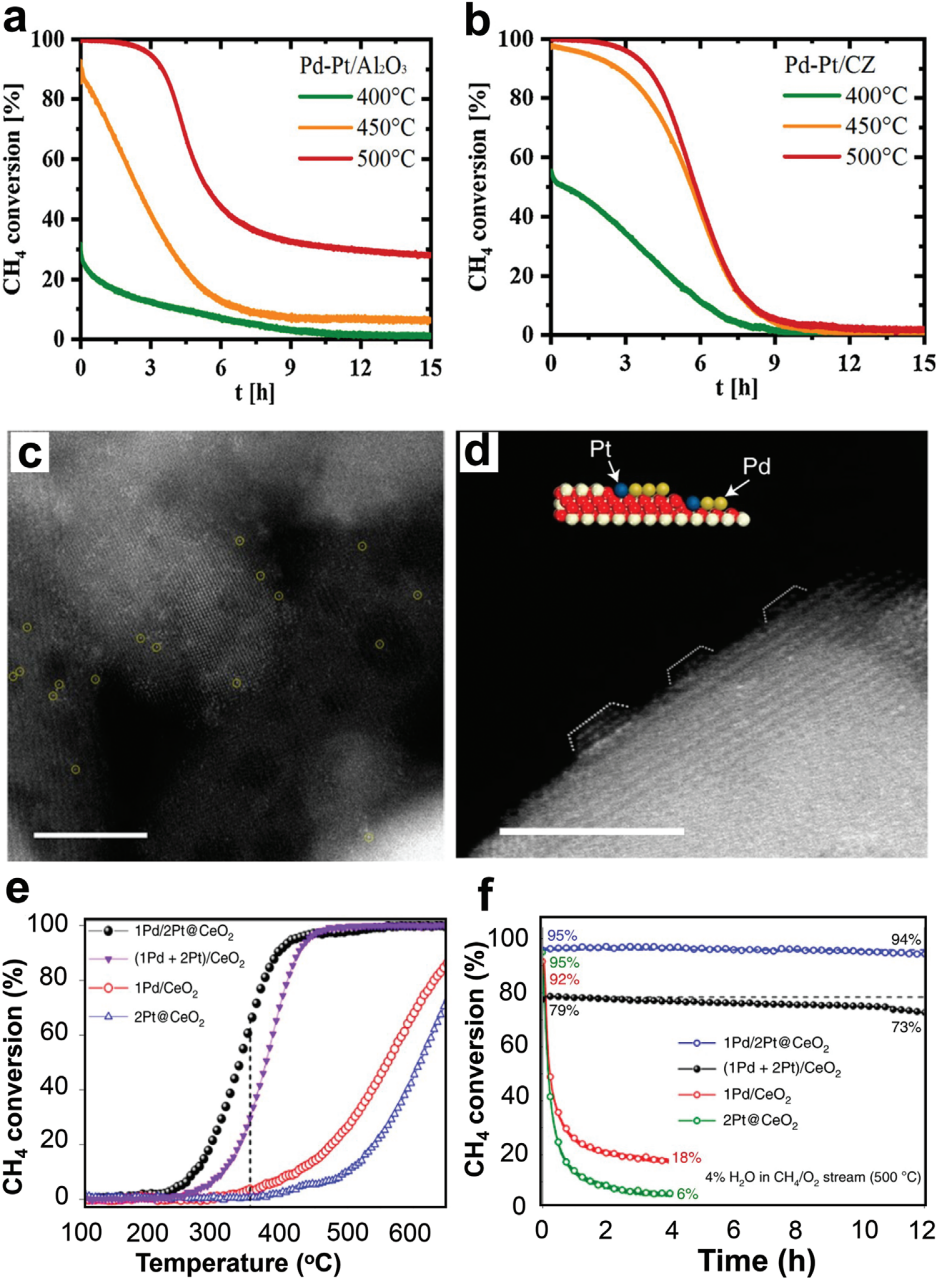
CH_4_ conversion as a function of time on stream over a) Pd–Pt/Al_2_O_3_ and b) Pt–Pt/CZ in the presence of 12% H_2_O and 5 ppm SO_2_. Reproduced with permission.^[^ ^225^ ^]^ Copyright 2020, Elsevier. c) AC‐STEM images of 2Pt@CeO_2_ synthesized through atom trapping showing single atoms of Pt. d) AC‐STEM image of Pd deposited on 2Pt@CeO_2_ ((1Pd/2Pt@CeO_2_), e) light‐off curves of CH_4_ oxidation without water vapor over different catalysts, f) comparison of different catalysts for CH_4_ combustion at 500 °C in 4% H_2_O demonstrating the outstanding ability of 1Pd/2Pt@CeO_2_ in wet conditions. Reproduced with permission.^[^ ^221^ ^]^ Copyright 2021, Springer Nature.

Reports portray that bimetallic catalysts exhibit better properties like improved activity, selectivity, and thermal stability than monometallic catalysts.^[^ ^220^, ^221^ ^]^ Thus, bimetallic catalysts such as Pt–Pd showed improved activity over monometallic catalysts at low temperatures.^[^ ^222^, ^223^ ^]^ Besides, the activity loss of Pd catalysts at low temperatures in the presence of water and SO_2_ is less severe after incorporating Pt.^[^ ^42^ ^]^ However, the effectiveness of the bimetallic sample for SO_2_ regeneration is reduced with increasing Pt content. For instance, Sulfur inhibition decreased when 10% Pt was substituted into a 0.9 Pd–0.1 Pt bimetallic sample, even though monometallic Pt is SO_2_ resistant.^[^ ^224^ ^]^ In addition, subsequent increases in Pt substitution resulted in decreased benefit, to the point that the reduction in activity with SO_2_ exposure was most significant at 70% Pt.^[^ ^42^, ^222^ ^]^ The poisoning severity also depends on the type of support, as recently reported by investigating Pd–Pt/Al_2_O_3_ and Pd–Pt/CZ CH_4_ oxidation catalysts under typical lean‐burn gas engine conditions for sulfur poisoning. Pd–Pt/Al_2_O_3_ maintained limited activity after the sulfur poisoning, and Pd–Pt/CZ was fully deactivated, as shown in **Figure** **12**a,b, highlighting the critical significance of the support.^[^ ^225^ ^]^ In addition, the 1Pd/2Pt@CeO_2_ catalyst's outstanding water resistance was also associated with the presence of Pd oxide in the form of 2D rafts on the support synthesized by atom trapping,^[^ ^221^ ^]^ as shown in Figure 12c–f. It is worth noting that all of the Pd*_x_* Pt alloy samples surpassed the monometallic Pd or Pt sample, which was not consistent with some reported results.^[^ ^226^ ^]^

The Pd promotion with Pt significantly reduces the CH_4_ activation temperature but increases the price of the catalyst.^[^ ^227^ ^]^ The literature has addressed several inexpensive and abundant promoters and supporters.^[^ ^228^ ^]^

Hence, Hu et al. investigated magnesia–alumina spinel (MgAl_2_O_4_) on the alumina support to stabilize the Pd–Pt catalyst. Because of MgAl_2_O_4_’s stabilization impact, the structural, chemical, and hydrothermal stability of the as‐prepared Pd–Pt bimetallic nanocatalyst was enhanced, and therefore the activation temperature for CH_4_ oxidation over the catalyst was reduced by at least 50 °C. The improvement might be attributed to higher dispersion, smaller particle size, and high quantity of the surface PdO on Mg‐modified alumina‐supported Pd–Pt catalysts (Pd–Pt/M–A).^[^ ^229^ ^]^ In addition, Pd–Pt/M–A showed a strong water resistance with a T_90_ of 425 °C. Hexaaluminates (LaMnAl_11_O_19_) were also proposed to decrease the activation temperature of CH_4_.^[^ ^230^ ^]^ Xu et al. synthesized Pd–Pt NPs supported on 3DOM LaMnAl_11_O_19_; their results revealed that the CH_4_ conversion (T_90_) over 0.97 Pd/3DOM LMAO was 343 °C, whereas that over 1.14PdPt_2.8_/3DOM LMAO was 456 °C. The increase in Pt content in metal alloy‐loaded samples depressed the catalytic activity. However, the T_90_ over 0.94Pt/3DOM LMAO was 515 °C, which was higher than those over 1.14PdPt_2.8_/3DOM LMAO.^[^ ^219^ ^]^ The results show that the noble metal alloy‐loaded sample with a higher Pd content can lower CH_4_ combustion's light‐off and combustion temperatures.^[^ ^231^ ^]^

#### 2.1.4 Other Noble Metal‐Based Catalysts

**Figure 13:**
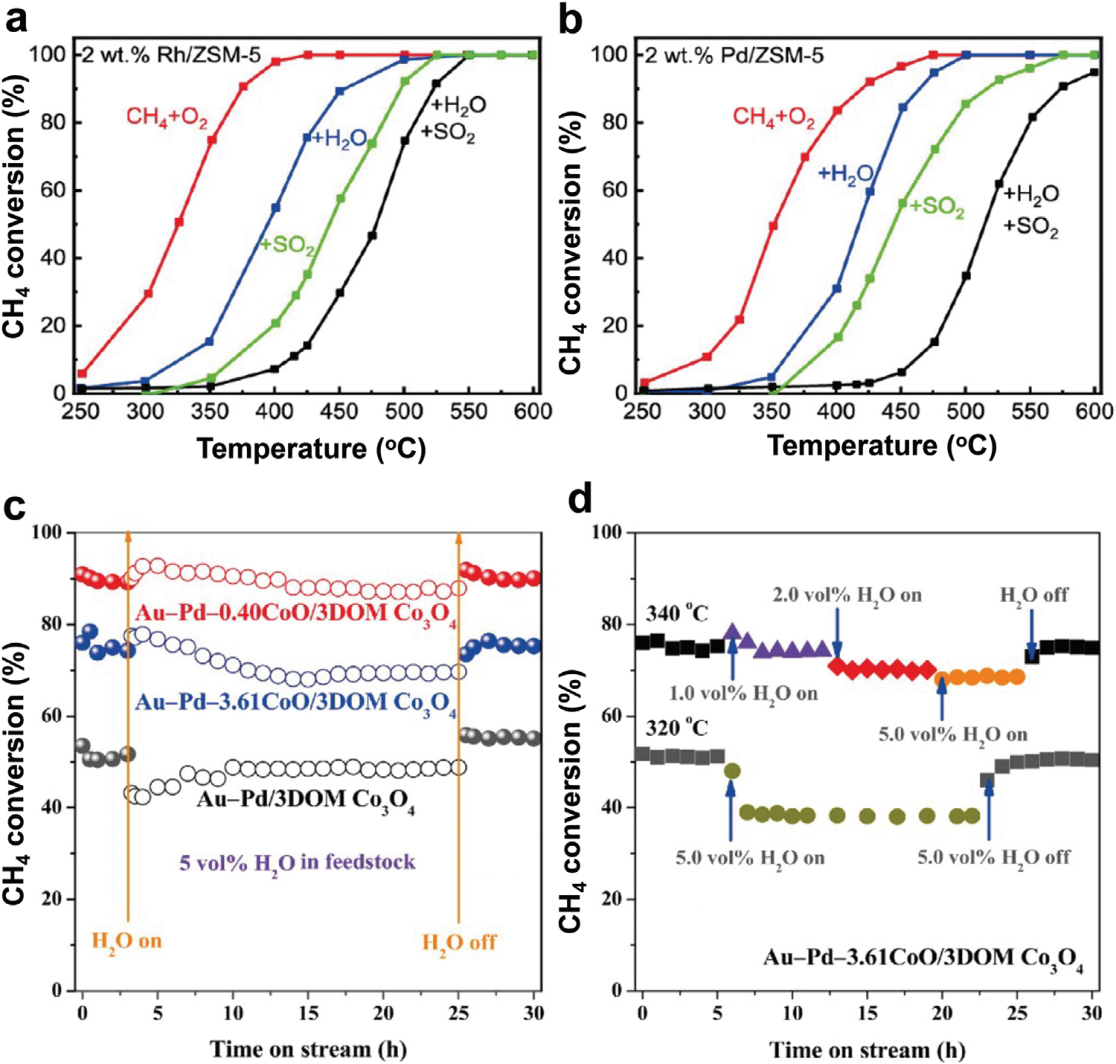
CH_4_ conversion on a) 2 wt% Rh/ZSM‐5 and b) 2 wt% Pd/ZSM‐5 under CH_4_ + O_2_ (red), CH_4_ + O_2_ + H_2_O (blue), CH_4_ + O_2_ + SO_2_ (green), and CH_4_ + O_2_ + H_2_O + SO_2_ (black) conditions. (2500 ppm CH_4_, 10 vol% O_2_, 5 vol% H_2_O, 20 ppm SO_2_, and N_2_. GHSV = 150 000 N mL (g_cat_ h)^−1^. Reproduced with permission.^[^ ^235^ ^]^ Copyright 2020, American Chemistry Society. c) Au–Pd–3.61CoO/3DOM as a reaction time in the absence or presence of 5.0 vol% water vapor at 340 °C and d) Au–Pd–3.61CoO/3DOM as a function of the reaction time in the presence of different water vapor. Reproduced with permission.^[^ ^251^ ^]^ Copyright 2017, American Chemistry Society.

The other noble metals were also investigated for CH_4_ activation at low temperatures.^[^ ^232^, ^233^, ^234^ ^]^ Rh has proven to be a crucial component of the three‐way catalyst, resulting in increased catalytic activity and stability; in an environment comprising H_2_O and SO_2_, it was discovered that Rh supported on ZSM‐5 outperformed Pd. Water's destabilizing effect on rhodium sulfate is considered to have a role in partially removing surface sulfate species from the rhodium oxide phase, minimizing sulfur poisoning, which is a significant component of Rh's superiority in the H_2_O and SO_2_ conditions.^[^ ^235^ ^]^ As depicted in **Figure** **13**a,b, Pd's relative activity loss caused by SO_2_ in a dry feed was less than Rh's,^[^ ^235^ ^]^ which confirms the good SO_2_ tolerance of Pd under dry conditions.^[^ ^162^ ^]^ The XRD data revealed that rhodium was available in the recently synthesized Rh/ZSM‐5 catalysts as relatively amorphous Rh_2_O_3_.^[^ ^236^ ^]^

Recently, Boukha et al. investigated the combustion of lean CH_4_ over Pd, Rh, Pt, and Ru catalysts supported on HAP by wetness impregnation. CH_4_ conversion begins at 225 °C in the Pd/HAP and Rh/HAP samples and gradually increases with temperature to reach 30% at 350 °C. Surprisingly, The Pd/HAP catalyst outperforms the Rh/HAP sample in terms of efficiency between 350 and 500 °C. Nonetheless, the Pt/HAP and Ru/HAP samples showed the lowest activity due to CH_4_ oxidation, which is only visible above 350 °C, and the performance at 500 °C does not exceed 35% and 15%, respectively. As a result, the catalyst efficiency follows the following pattern: Pd/HAP > Rh/HAP > Pt/HAP > Ru/HAP. More importantly, there was no relationship between performance and metallic dispersion.^[^ ^204^ ^]^ Hence, the increased oxygen storage capacity of Pd/HAP and Rh/HAP samples explains their superiority.^[^ ^237^ ^]^ Even though the high cost of Rh should be considered when assessing the business case for Rh catalysts, it provides high conversion at a low temperature, which is a significant development that holds tremendous promise for creating solutions to the CH_4_ activation problem.

Ir‐based catalysts are more active and durable; Ir has more catalytic activity than Rh.^[^ ^238^ ^]^ Recent theoretical calculations suggest that IrO_2_ and Ir have the unique ability to activate CH_4_ at low temperatures.^[^ ^239^ ^]^ According to Liang et al., the cleavage of the C—H bond of CH_4_ took place on the IrO_2_ (110) surface at −123 °C.^[^ ^240^ ^]^ The IrO_2_ (110) layer has been identified as the active phase, as was previously predicted by Wang et al. using DFT calculations.^[^ ^241^ ^]^ In addition, DFT was also used by Fung et al. to demonstrate that the C—H bond in CH_4_ might be activated by the Ir single atoms on TiO_2_ (110) below 25 °C.^[^ ^242^ ^]^ Despite having a wide range of prospective applications, the oxidation of CH_4_ using Ir‐based catalysts has barely been assessed, and their activity and stability are significantly inferior to those of Pd‐based catalysts.^[^ ^233^, ^243^ ^]^ According to certain research, IrO*_x_* particle size reduction and the coexistence of metallic Ir and IrO_2_ could enhance IrO*_x_*’s reducibility and catalytic performance.^[^ ^243^ ^]^ Indeed, the good dispersion and stabilization of IrO_2_ NP are still major obstacles under oxidizing environment.^[^ ^241^ ^]^ Chen et al. proposed a strategy for redispersion caused by an SMSI, with redispersion occurring during high‐temperature H_2_ reduction and in situ oxidation under mild oxidative environment.^[^ ^244^ ^]^ The results suggested that the as‐developed Ir/TiO_2_‐HO with ultrafine IrO_2_/Ir complex active sites exhibits a much‐increased apparent activity. The T_99_ over Ir/TiO_2_‐HO was as low as 320 °C, exposing superior CH_4_ catalytic activity (**Table** **3**) and excellent durability than the as‐prepared Pd/*γ*‐Al_2_O_3_.^[^ ^245^ ^]^ The ultrafine dispersion, proper chemical state, and metal–support solid interaction all contribute to enhancing performance.

**Table 3:** Summary of other noble metal‐based catalysts for CH_4_ activation

Catalyst	Metal loading [wt%]	Preparation method	Calc. temp. [°C]	Particle size [nm]	CH_4_ conc. [%]	Catalyst amount [mg]	GHSV [mL g^−1^ h^−1^]	T_10_ [°C]	T_50_ [°C]	T_90_ [°C]	Refs.
Pt/ZrO_2_	1	Impregnation	500		0.2	200		312	369	497	[210]
PdPt/3DOM LMAO	1.14	Gas‐bubble‐assistant adsorption	550	3–5	1.5	50	20 000	284	308	456	[219]
Pd–Pt/*γ*‐Al_2_O_3_	2.4	Incipient wetness Impregnation	700	5.3		300		350	402	430	[225]
Pd–Pt/M–A	1.5	Incipient wetness impregnation	550				330 000	315	368	425	[229]
Pd_2.41_Pt	56	Impregnation	400		2.5	20	100 000	272	303	322	[253]
Rh/ZSM‐5	2	Incipient wetness impregnation	600			120		260	325	375	[235]
Ir/TiO_2_–HO	1	Ultrasonic‐assisted incipient‐ wetness impregnation	500	1.09	1	200	30 000	239	267	289	[244]
Au–Pd–0.4CoO/3DOM Co_3_O_4_	2	Gas bubble‐assisted adsorption	400	3.6	2.5	50		260	312	341	[251]

By contrast, RuO_2_ is known to be a much better total oxidation catalyst than IrO_2_. Hence, Khalid et al. anticipated that mixed Ru*_x_* Ir_1‐_ *_x_* O_2_ supported on rutile‐TiO_2_ powder might offer synergy effects for the combustion of CH_4_ by improving its activation step by Ir. The findings demonstrated that the activity of Pd@Al_2_O_3_, Ir 100@TiO_2_, and Ru 75@TiO_2_ are comparable. A direct comparison of the Ir_100@TiO_2_ and Ru_75@TiO_2_ with Pd@TiO_2_ shows that T_90_ is 44 to 66 °C lower. Nevertheless, compared to Pd@TiO_2_, the amount of CH_4_ supplied was two times higher, and the active component was around two to three times lower. The Ir in the mixed Ru*_x_* Ir_1−_ *_x_* O_2_ oxide facilitates effective CH_4_ activation, while Ru enhances and promotes the subsequent oxidation processes of the methyl group to form CO_2_.^[^ ^246^ ^]^

When focusing on incorporating a noble metal into the active sites of Pd, gold with a specific size is an excellent candidate as it is an effective catalyst for oxidation reactions.^[^ ^247^ ^]^ Because the inclusion of Au can help keep Pd components stable, reduce the product's desorption temperature, and change the nature of the active sites.^[^ ^248^ ^]^ PdAu bimetallic catalysts are promising for CH_4_ activation. In order to maximize the activity of the Pd‐based catalyst and prevent deactivation in CH_4_ combustion, researchers increased the functionalization of Pd with Au and improved their relationship with oxide supports.^[^ ^249^ ^]^ Three‐dimensionally ordered macroporous (3DOM) materials were also suggested as Au catalyst carriers because of their easy transportation and diffusion characteristics.^[^ ^250^ ^]^ Xie et al. reported the Au–Pd–*x*CoO/3DOM Co_3_O_4_ (*x* is the Co/Pd molar ratio) catalysts by adding some CoO to the Au–Pd alloy NPs. The Au–Pd–*x*CoO/3DOM Co_3_O_4_ nanocatalysts showed excellent thermal stability and water resistance capacity, as depicted in Figure 13c,d.^[^ ^251^ ^]^ Han et al. claimed that 1.98AuPd_2.1_/18.20Co_3_O_4_/3DOM MnCo_2_O_4_ exhibited a higher catalytic activity with T_90_ of 408 °C. The partial deactivation due to water vapor or CO_2_ was reversible. The catalytic activity reduced in the sequence of 1.98AuPd_2.1_/18.20Co_3_O_4_/3DOM MnCo_2_O_4_ > 1.10AuPd2.0/18.20Co_3_O_4_/3DOM MnCo_2_O_4_ > 0.56AuPd_1.9_/18.20Co_3_O_4_/3DOM MnCo_2_O_4_ > 18.20Co_3_O_4_/3DOM MnCo_2_O_4_ > 3DOM MnCo_2_O_4_, indicating that the appropriate loadings of Co_3_O_4_ and AuPdz NPs on the surface of 3DOM MnCo_2_O_4_ might improve the catalytic activity. The good catalytic performance of 1.98AuPd_2.1_/18.20Co_3_O_4_/3DOM MnCo_2_O_4_ was attributed to its high adsorbed oxygen species concentration, good low‐temperature reducibility, and strong interaction between Co_3_O_4_ or AuPd_2.1_ NPs and 3DOM MnCo_2_O_4_.^[^ ^252^ ^]^

### 2.2 Methane Activation over Transition Metal‐Based Catalysts

Due to noble metals’ high cost and inferior antisintering, transition metal oxide catalysts have been explored as an alternative solution for CH_4_ activation at low temperatures.^[^ ^254^, ^255^, ^256^ ^]^ Catalytic activities in CH_4_ combustion of the various oxides prepared by precipitation method were reported to follow the order of Co_3_O_4_ > Mn_2_O_2_ > CuO > NiO.^[^ ^257^, ^258^ ^]^ However, Liu et al. reported NiO prepared through the thermal decomposition of the corresponding metal nitrates to be a more effective catalyst for CH_4_ oxidation than Co_3_O_4_.^[^ ^259^ ^]^ The above disagreement demonstrates the importance of the catalyst preparation method for CH_4_ activation. This section will discuss different factors that influence the activation of CH_4_ over transition metal‐based catalysts at low temperatures.

#### 2.2.1 Cobalt‐Based Oxide Catalysts

Cobalt‐based oxides catalysts are well known due to their structural and electronic features of spinel‐type oxides with varying valence states (Co^2+^/Co^3+^), and the reduced bonding energy of Co—O bonds demonstrates the necessary activity for CH_4_ oxidation at lower temperatures.^[^ ^260^, ^261^ ^]^ Studies revealed that their catalytic activities are strongly influenced by their morphologies and may be related to the electron transport capabilities of various Co_3_O_4_ nanoparticles. Hence, Co_3_O_4_ catalysts with various morphologies (sphere, nanorod, nanobelt, nanocube, nanowire, nanoplate, hollow, hierarchical, and so on) have been created.^[^ ^83^, ^262^, ^263^, ^264^ ^]^ For instance, the Co_3_O_4_ nanotubes prepared through the interfacial reaction of NaOH with prefabricated CoC_2_O_4_·2H_2_O nanorods showed high activity for CH_4_ catalytic combustion with complete CH_4_ conversion at 325 °C for Co_3_O_4_ nanotubes calcinated at 350 °C (Co_3_O_4_ NTs‐350) while conversion over Co_3_O_4_ nanoparticles calcinated at 400 (Co_3_O_4_ NPs‐400 °C) was only 55%.^[^ ^264^ ^]^ In addition, the Co_3_O_4_ NTs‐350 °C was still obviously more active than Co_3_O_4_ nanorods (Co_3_O_4_ NRs).^[^ ^265^ ^]^ The supremacy of Co_3_O_4_ nanotubes can be attributed to the presence of adsorbed and lattice oxygen of higher reactivity, as shown by H_2_‐TPR and O_2_‐TPD studies.^[^ ^264^ ^]^ It is worth mentioning that among all reported transition metal‐based catalysts for CH_4_ activation at low temperatures, Co_3_O_4_ NTs‐350 showed the lowest temperature with T_10_, T_50_, and T_90_ at 170, 235, and 295 °C, as shown in **Table** **4**. It has also been reported that Co_3_O_4_ nanosheets exhibit higher activity than the corresponding nanobelts or nanocubes.^[^ ^262^ ^]^ Different researchers also found similar results confirming that the activity in CH_4_ oxidation strongly correlates with the Co_3_O_4_ structure.^[^ ^87^, ^266^, ^267^ ^]^

**Table 4:** Summary of transition metal‐based catalysts for CH_4_ activation

Catalysts	Preparation method	Calc. temp. [°C]	Morphology	CH_4_ conc. [%]	Catalyst amount [mg]	GHSV [mL g^−1^ h^−1^]	T_10_ [°C]	T_50_ [°C]	T_90_ [°C]	Refs.
Co_3_O_4_‐200	Calcination	200		1	200	18 000	220	270	360	[327]
Co_3_O_4_ NTs‐350	Interfacial reaction	350	Nanotubes	2		6000	170	235	295	[264]
Co_3_O_4_			Nanoplates		300		175	263	320	[328]
			Nanorods				175	274	340	
			Nanoparticles				200	285	360	
			Mesoporous				200	340	452	
			Microporous				200	412	535	
H‐Co_3_O_4_‐3h	Acid treatment		Flake hexagonal	2	100	33 000	285	311	382	[270]
CoO*_x_* @SiO_2_‐400	Spontaneous deposition	400		2	1000	15 000	240	290	310	[277]
CoCr_2_O_4_ (8 h)	Solvothermal		Nanospheres			180 000	318	396	453	[329]
Co–In‐0.2	Coprecipitation	400		2	100	48 000	262	325	380	[289]
ZrO_2_(2)–Co_3_O_4_	Coprecipitation	500		0.5	200	12 000	210	278	335	[273]
N‐Co_3_O_4_‐110		300		2	100	78 100	302	342	412	[274]
MnO_2_‐C	Hydrothermal process		Nanocubic				260	293	350	[298]
MnO_2_‐R			Nanorods				280	342	393	
MnO_2_	Hydrothermal process		Wire	7	200	24 000	350	375	415	[299]
			Tube				350	380	445	
			Flower				390	435		
*α*‐MnO2	One‐step hydrothermal			0.1			259	315	373	[302]
MnO_2/_ZrO_2_	One‐step hydrothermal				200	90 000	259	329	488	
NiC_2_O_4_		300		0.8	100	12 000		360	412	[305]
NiO‐NSL	Solid–liquid precipitation	500	Rods	1	40	30 000		340	390	[308]
NiO NPs	Thermal decomposition			0.2	150	40 000	300	350	400	[309]
NiO‐SPP				0.2	150	40 000	350	400	445	
NiO/CeO_2_	Impregnation	450		1	200	60 000	330	415	467	[314]
Ni_0.89_Zr_0.11_O_2−*δ*_		450		1	300	30 000	260	340	380	[323]

**Figure 14:**
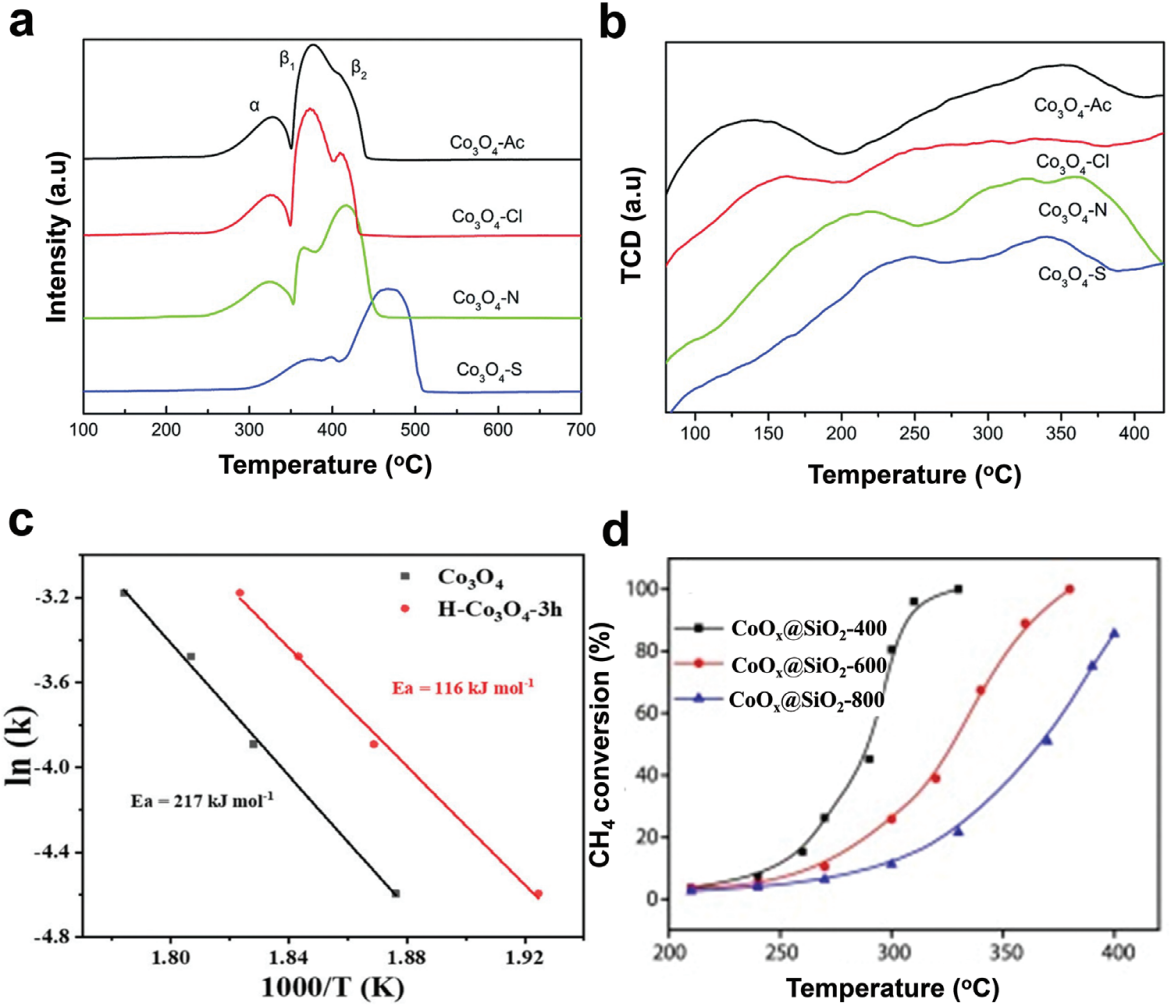
a) H_2_‐TPR and b) O_2_‐TPD patterns of Co_3_O_4_ catalysts synthesized with different cobalt precursors. Reproduced under the terms of the Creative Commons CC‐BY license.^[^ ^268^ ^]^ Copyright 2020, Royal Society of Chemistry. c) Arrhenius plots. Reproduced with permission.^[^ ^270^ ^]^ Copyright 2021, Royal Society of Chemistry. d) Catalytic activity in CH_4_ oxidation as a function of the reaction temperature. Reproduced with permission.^[^ ^277^ ^]^ Copyright 2019, Elsevier.

Moreover, the synthesis procedure significantly affects the structural and textural properties of the catalysts, like component dispersion, specific surface area, particle size, and interparticle interactions, which in turn influences the redox properties and catalytic performances of the final catalysts (**Figure** **14**a,b).^[^ ^268^ ^]^ Various methods have been applied in the preparation of Co_3_O_4_ oxides. Pu et al. synthesized a series of Co_3_O_4_ catalysts by a facile precipitation method by changing the aging time. It was noticed that as the aging time increased, the activity for the reaction enhanced and then decreased. The results indicated that the sample aged for 8 h (Co_3_O_4_‐8) had the highest ratio of *A*_Tetrahedral_/*A*_Octahedral_ at the catalyst's surface.^[^ ^269^ ^]^ Bao et al. found that acid etching could selectively erase part of Co_3_O_4_ with poor crystallinity, leading to an increased specific surface area, lowering the Co valence state, and forming more oxygen vacancies. The acid‐leached sample demonstrated outstanding showed performance for CH_4_ oxidation with a T_90_ of 382 °C, lower than 411 °C for the pristine Co_3_O_4_ (Figure 14c). The universality of the acid etching strategy was further verified toward a MnO_2_ catalyst.^[^ ^270^ ^]^

At present, the primary goal of research on Co_3_O_4_ catalyst is to improve the stability of the catalyst by the introduction of support,^[^ ^271^ ^]^ the synergy between catalyst and support,^[^ ^272^ ^]^ preparation of nanoscale catalysts with unique morphology to expose more highly active sites, and dope transition metal or rare earth as catalytic promoters.^[^ ^273^, ^274^ ^]^ For instance, the catalyst prepared by Li et al. through the coprecipitation process reached 90% of CH_4_ conversion at 320 °C, a lower temperature than pure Co_3_O_4_. Manganese‐enhanced crystal defections likely increased the quantity of octahedrally coordinated divalent cobalt cations involved in catalytic activity. Hydroxyl groups on manganese‐doped catalysts might also explain the promoting effects.^[^ ^275^ ^]^ Indeed Chang et al. highlighted that MnO_2_ stimulates the generation of reactive oxygen species by altering the lattice structure of Co_3_O_4_.^[^ ^276^ ^]^ Recently using SiO_2_ as the carrier and manganese ion as the active promoter, Mn_1_/Co/SiO_2_ catalyst showed higher Co^3+^/Co^2+^ and O_II_/O_I_, indicating higher mobility of reactive oxygen species, which is more conducive to CH_4_ catalysis.^[^ ^271^ ^]^ SiO_2_ was also used to prepare CoO*_x_* @SiO_2_‐T (T is calcination temperature) using a spontaneous deposition method. The catalytic performance for CH_4_ oxidation decreases following the order of CoO*_x_* @SiO_2_‐400, CoO*_x_* @SiO_2_‐600, and CoO*_x_* @SiO_2_‐800. The CoO*_x_* @SiO_2_‐400 catalyst showed the maximum activity with a 100% CH_4_ conversion at 330 °C due to its rich surface‐active oxygen and more active sites (Figure 14d). Furthermore, the comparable supported CoO*_x_*/SiO_2_‐400 catalyst was also synthesized; nevertheless, its activity was lower than that of the CoO*_x_* @SiO_2_‐400 catalyst, demonstrating a 60% CH4 conversion at 400 °C.^[^ ^277^ ^]^

The fast exothermic nature of the CH_4_ oxidation reaction has a significant negative impact on the life of catalysts and reduces productivity or selectivity.^[^ ^115^, ^278^ ^]^ Thus, a new method implementing heat management in heterogeneous chemical reactions using phase change materials was proposed to reduce the activation temperature of CH_4_.^[^ ^279^, ^280^ ^]^ Recently, Li et al. constructed the thermal storage functional catalysts (Co_3_O_4_/(SiAl@Al_2_O_3_)). The reported catalyst behaves both as the catalytic and thermal management functions and improves the stability and uniformity of the temperature in the catalyst bed. Finally, SiAl@Al_2_O_3_‐30 wt% achieved high efficiency on the lean CH_4_ combustion with the competitive activity of T_90_ at 370 °C, which is higher than the pure Co_3_O_4_ with T_90_ at 425 °C. Additionally, its CH_4_ conversion may reach 50% even after 37 min without the heat source, which is twice as effective as pure Co_3_O_4_.^[^ ^281^ ^]^ *γ*‐Al_2_O_3_ is frequently used as support to enhance the structural and textural properties of the Co_3_O_4_ active phase.^[^ ^271^, ^282^ ^]^

However, the common cobalt–alumina combination might cause the conversion of Co_3_O_4_ into cobalt aluminate (CoAl_2_O_4_), an inert species from which cobalt cannot be effectively retrieved.^[^ ^283^ ^]^ A similar process happens with the materials with a high specific surface, such as silica; they interact with cobalt to produce inactive phases, such as cobalt silicate solid solutions that have a deleterious impact on the performance of the resulting supported catalysts.^[^ ^284^ ^]^ As a result, a promoter might be used to adjust the support to tune its high affinity for deposited cobalt.^[^ ^285^ ^]^ However, a common side effect is the generation of stable mixed oxides between cobalt and the promoter, which have lower specific activity than Co_3_O_4_.^[^ ^286^ ^]^ Different promoters were proposed for spinel catalysts, including Cr, Cu, Ni, Zn, In, Zr, and Ce.^[^ ^273^, ^287^, ^288^, ^289^ ^]^

**Figure 15:**
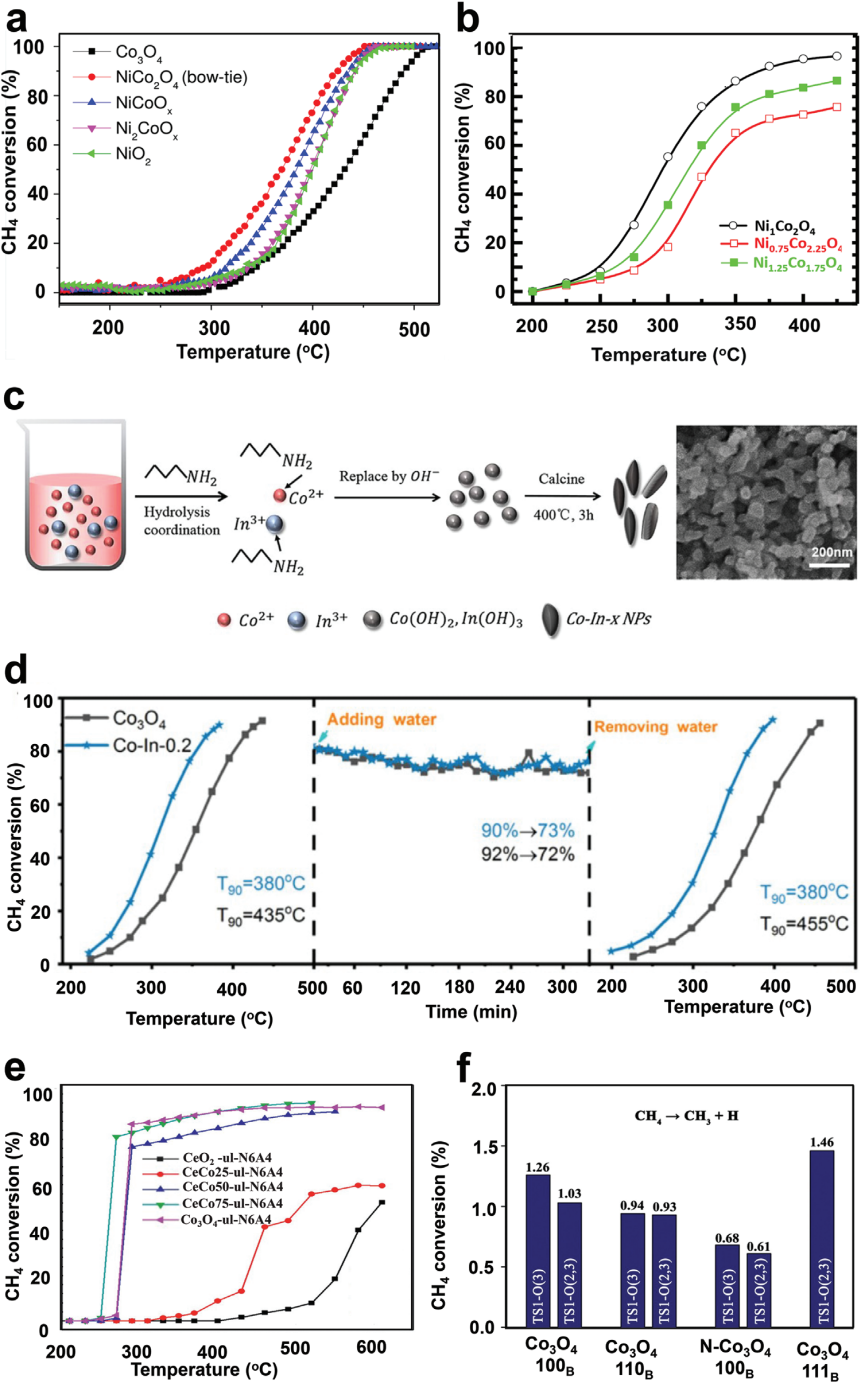
a) Ni/Co oxides prepared with varying Ni/Co ratios. Reproduced with permission.^[^ ^287^ ^]^ Copyright 2019, Wiley‐VCH. b) Ni/Co with different Ni atomic fractions. Reproduced with permission.^[^ ^290^ ^]^ Copyright 2015, Springer Nature. c) Synthesis diagram of the Co–In‐*x* nanoparticles via a modified precipitation method. d) Cyclic and long‐term stability tests over Co_3_O_4_ and Co–In‐0.2 catalyst under 5 vol% moisture conditions. Reproduced with permission.^[^ ^289^ ^]^ Copyright 2020, American Chemistry Society. e) Co–Ce–O composite oxide catalysts. Reproduced with permission.^[^ ^294^ ^]^ Copyright 2016, Elsevier. f) Energy barriers for the activation of the first C—H bond in CH_4_ on different surfaces. Reproduced with permission.^[^ ^274^ ^]^ Copyright 2020, Elsevier.

Due to their identical ionic radius, coordination, coordination, and oxidation states, recently, nickel promoter was directly added to the cobalt spinel phase. The simultaneous addition of nickel and cobalt mixture onto the alumina enhanced the redox properties by favoring the production of nickel cobaltite‐like species and partially reducing the interaction between Co_3_O_4_ and alumina. The improvement of the redox properties promoted the specific reaction rate and a shift of around 50 °C in the T_50_ value over the bimetallic catalysts.^[^ ^284^ ^]^ The Co–Ni catalysts were comparatively stable for extended periods, but they experienced a significant and irreversible deactivation in the presence of water vapor.^[^ ^284^ ^]^ In addition, due to the desirable intrinsic property, the appropriate mixing of Ni with Co achieved higher activity for CH_4_ combustion. For instance, Dai et al. reported the catalytic activity toward CH_4_ to follow the order of NiCo_2_O_4_ > NiCoO*_x_* > Ni_2_CoO*_x_* ≈ NiO_2_ > Co_3_O_4,_ as shown in **Figure** **15**a.^[^ ^287^ ^]^ Tao et al. explored the complete oxidation of CH_4_ on NiCo_2_O_4_ at a molecular level. As shown in Figure 15b, it was noticed that the higher catalytic activity of NiCo_2_O_4_ at low temperatures results from CH_4_ dissociating to methyl on nickel cations and then coupling with surface lattice oxygen atoms. Thus, Ni cations were proposed as active sites for CH_4_ oxidation instead of Co.^[^ ^290^ ^]^ It has also been found that the concentration of tetrahedral sites Co^2+^ cations play a crucial role in its catalytic activity for CH_4_ combustion.^[^ ^269^ ^]^ Hence, the appropriate incorporation of metal oxides may exhibit higher activity, reduce CH_4_ activation temperature and provide thermal stability.

A series of CoCr_2_O_4_ with various Co/Cr ratios prepared through coprecipitation and solvothermal methods revealed improved catalytic activity with 90% conversion at a temperature of less than 500 °C and excellent resistance to water vapor.^[^ ^254^ ^]^ At the same time, the CoCr_2_O_4_ prepared by a sol‐gel method showed a 90% conversion of CH_4_ at 750 °C.^[^ ^291^ ^]^ Lim et al. claim that mesoporous MCo_2_O_4_ spinel catalysts (m‐MCo_2_O_4_) synthesized via the nanoreplication method demonstrated higher catalytic activity for CH_4_ combustion than bulk counterparts (b‐MCo_2_O_4_). The T_90_ of m‐CuCo_2_O_4_ (440 °C), m‐ZnCo_2_O_4_ (450 °C), and m‐NiCo_2_O_4_ (458 °C) were 89, 101, and 29 °C lower than that of b‐CuCo_2_O_4_, b‐ZnCo_2_O_4_, and b‐NiCo_2_O_4_, respectively. Consequently, the T_90_ of the mesocatalysts is markedly lower than those of the bulk catalysts. According to XPS, the improved performance of the m‐CuCo_2_O_4_ spinel catalyst is due to the high normalized amount of Co^3+^ cations on the surface.^[^ ^288^ ^]^ Similarly, using an organic base (N‐butylamine) as a precipitator in a modified precipitation process shown in Figure 15c, Xiao et al. doped In^3+^ into octahedral sites of the spinel Co_3_O_4_ to make Co_3_O_4_–In_2_O_3_ catalyst with the particle size of 10–20 nm. Compared to Co_3_O_4_, the comparatively low doping concentration of In^3+^ (0.10.3) resulted in increased activity. By contrast, the high doping content (0.4) negatively influenced the catalytic activity. Furthermore, Co–In‐0.2 demonstrated a significantly stronger activity, with a T_99_ CH_4_ conversion of 395 °C, which was 65 °C lesser than pure Co_3_O_4_. Surprisingly, Co–In‐0.2 performed the best in the presence of water with T_10_, T_50,_ and T_90_ at 240, 310, and 380 °C, respectively, as depicted in Figure 15d. Therefore, the superior catalytic activity of Co_3_O_4_–In_2_O_3_ composite oxides might be due to the increased Co^2+^ ratio and abundant reactive oxygen species on the surface; it also displays outstanding stability and water resistance.^[^ ^289^ ^]^ Pu et al. also synthesized a series of ZrO_2_Co_3_O_4_ catalysts with spinel structure by coprecipitation method; the results showed that a pure Co_3_O_4_ exhibits considerable activity with T_90_ of 377 °C. The incorporation of ZrO_2_ species remarkably improved the activity of catalysts, and the conversion curves shifted to lower temperatures with T_90_ of about 335 and 340 °C in dry and wet conditions, respectively. However, the activity decreases significantly with the increase in ZrO2 content up to 5% or higher. These findings imply that increased activity in lean CH_4_ oxidation might be attributed to high surface areas, *A*_Tetrahedral_/*A*_Octahedral_, and surface‐active oxygen species.^[^ ^273^ ^]^

Recent work of Choya et al. compared different strategies to improve the activity of Co_3_O_4_/Al_2_O_3_ catalyst by the alumina surface protection with MgO or CeO_2_ (0.20 g g^−1^) to prevent CoAl_2_O_4_ formation. The Co/Al_2_O_3_–CeO_2_ catalyst exhibited enhanced activity than the other studied samples.^[^ ^285^ ^]^ Furthermore, adding Co_3_O_4_ to CeO_2_ boosted catalytic activity for the oxidation reaction, which might be ascribed to the better redox properties and high oxygen storage capacity caused by the synergistic effect between CeO_2_ and Co_3_O_4_.^[^ ^292^, ^293^ ^]^ Innovative Co–Ce mixed oxide catalysts were recently synthesized using various synthesis methods. For instance, Co_3_O_4_/CeO_2_ composites prepared by a new sol–gel method, which combined ultrasonic impregnation treatment and calcination in an N_2_ atmosphere, showed that the modified catalyst had a complete CH_4_ combustion conversion (100%) at a low temperature of 364 °C, as illustrated in Figure 15e, which might be due to the mesoporous structure, comparatively higher amount of surface oxygen and larger oxygen vacancies.^[^ ^294^ ^]^ The results demonstrated that ultrasonic impregnation treatment combined with the N_2_ thermal treatment prior to calcination in the air had a promising application for preparing Co_3_O_4_/CeO_2_ composite catalysts.

Furthermore, heteroatom doping is an effective technique for modulating the surface and electronic structure of the nanomaterial.^[^ ^295^ ^]^ It was envisioned that N‐doping into Co_3_O_4_ might offer the possibility of inducing anionic defects and providing oxygen vacancies, making the oxidation reaction more appealing.^[^ ^296^ ^]^ Considering that the CH_4_ oxidation reaction over Co_3_O_4_ is surface‐sensitive, Yu et al. doped nitrogen to a more active exposed facet (Co_3_O_4_‐110). Consequently, the catalyst showed significantly improved CH_4_ oxidation activity with a T_50_ of 342 °C, about 42 °C, less than the pristine sample, and showed superior hydrothermal stability. Indeed, the energy barriers for C—H cleavages dropped after N‐doping, suggesting that C—H bonds are ready to be activated, as shown in Figure 15f.^[^ ^274^ ^]^ The higher performance was related to the defective structure, improved redox property, and highly exposed active sites. Meantime, N‐doping plays a vital role in activating Co_3_O_4_ by increasing the electrophilicity of surface oxygen and decreasing the activation energy of C—H bonds.

#### 2.2.2 Manganese‐Based Catalysts

**Figure 16:**
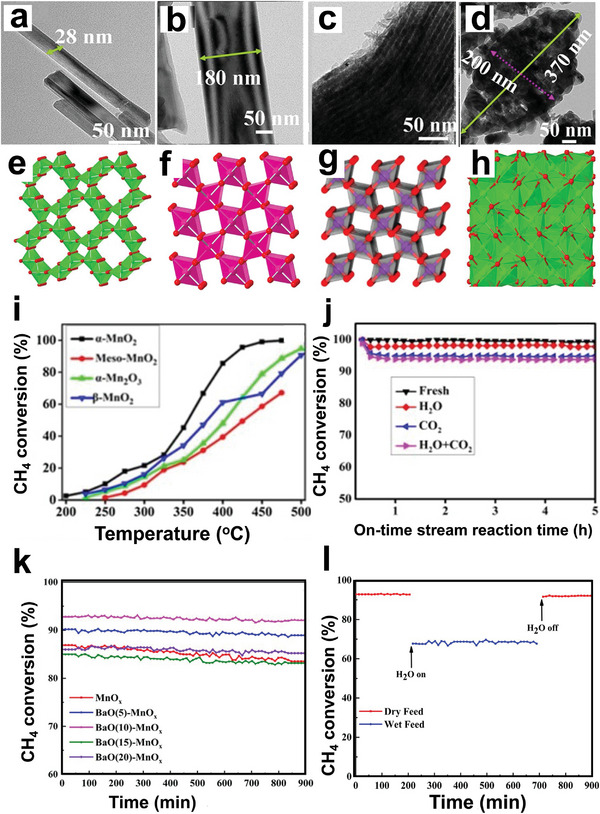
TEM images of a) *α*‐MnO_2_, b) *β*‐MnO_2_, c) Meso‐MnO_2_, and d) *α*‐Mn_2_O_3_. Structural depictions of e) *α*‐MnO_2_, f) *β*‐MnO_2_, g) Meso‐MnO_2_, and h) *α*‐Mn_2_O_3_. i) CH_4_ oxidation as a function of temperature and j) effect of 9.5 vol% H_2_O and 10%CO_2_ on CH_4_ oxidation at 475 °C in *α*‐MnO_2_. Reproduced with permission.^[^ ^297^ ^]^ Copyright 2018, Elsevier. k) Catalytic stability over the BaO–MnO*_x_* catalysts with various Ba contents at 450 °C and l) the effect of H_2_O over the BaO(10)–MnO*_x_* catalyst calcined at 500 °C for CH_4_ stability. Reproduced with permission.^[^ ^303^ ^]^ Copyright 2021, Elsevier.

The catalytic properties of pure manganese oxides are commonly related to the wide range of oxidation states recognized for the manganese ions, in addition to high oxygen mobility in the crystalline lattice.^[^ ^297^ ^]^ The diverse polymorphic structures of Mn oxides, such as *α*‐ and *β*‐MnO*_x_*, reveal different ways of linking together the fundamental octahedral [MnO_6_] units, resulting in dramatically differing catalytic activity.^[^ ^94^ ^]^ The influence of the crystalline structure of MnO_2_ on CH_4_ oxidation was pointed out.^[^ ^297^ ^]^ The *α*‐MnO_2_ exhibits nanowire morphology with a 28 nm diameter (**Figure** **16**a) as well as interplanar distances of 0.473 and 0.278 nm. On the other hand, *β*‐MnO_2_ shows a nanorod morphology (Figure 16b) with an interplanar distance of 0.313 nm attributed to the MnO_2_ (110) facet. At the same time, meso‐MnO_2_ and *α*‐Mn_2_O_3_ revealed an ordered mesoporous framework (Figure 16c) with the highest surface area and flower‐like with a cubic phase (Figure 16d), respectively. It is worth mentioning that *α*‐MnO_2_, *β*‐MnO_2_, meso‐MnO_2_, and *α*‐Mn_2_O_3_ present different structures, as shown in Figure 16e–h. The results from the CH_4_ performance test demonstrated *α*‐MnO_2_ to be the most active phase due to high surface Mn^4+^ concentration, more active oxygen species, and excellent reducibility (Figure 16i). In addition, as indicated in Figure 16j, no significant change was reported for *α*‐MnO_2_ in the presence of 9.5 vol% H_2_O.^[^ ^297^ ^]^ Zhang et al. synthesized Mn oxides with nanocubic (MnO_2_‐C) and nanorods (MnO_2_‐R) morphology by changing the precursors. The results suggest that catalytic efficiency in MnO_2_‐C was higher than that of MnO_2_‐R. Specifically, the T_90_ value of MnO_2_‐C was 350 °C, which is much lower than that of MnO_2_‐R at 393 °C. The MnO_2_‐C superior catalytic performance was associated with higher surface area, smaller crystalline size, more surface oxygen vacancies, and better low‐temperature reducibility.^[^ ^298^ ^]^ Meanwhile, Zhang et al. reported the wire‐like MnO_2_ to perform better than other various morphologies of the MnO*_x_* nanocrystalline catalysts, including rod, tube, and flower, with a T_90_ of 400 °C.^[^ ^299^ ^]^

It has been reported that the addition of Mn to Cu–(Mn)–Zn–Mg–Al mixed oxides catalysts widely enhances the catalytic activity by increasing the reduction properties of copper and raising various active phases.^[^ ^300^ ^]^ Another study looked at the catalytic performance of MnO*_x_*–NiO catalysts with varying manganese oxide loadings.^[^ ^301^ ^]^ The reported catalysts exhibited substantially greater catalytic activity at a lower temperature due to Mn–Ni–O solid solution grains, which prevented nickel oxide from growing. The maximum performance for pure MnO*_x_* was 40% even temperature up to 450 °C, whereas the 90% CH_4_ conversion temperature for the MnO*_x_* (0.13)–NiO catalyst was ≈390 °C. However, by including additional substances as promoters, the catalytic efficiency of the manganese oxide‐based catalysts can be significantly enhanced. Jia et al. showed that the activity of the *α*‐MnO_2_/ZrO_2_ prepared by a one‐pot hydrothermal method with various Mn/Zr ratios could exhibit high activity for CH_4_ catalytic oxidation and even better than that of 1% Pt/Al_2_O_3_. T_50_ of MnO_2_/ZrO_2_ catalysts were located in the range of 315–335 °C, whereas that of Pt/Al_2_O_3_ was 380 °C. In addition, after sulfur aging, the MnO_2_/ZrO_2_ catalysts with Mn/Zr ratios of 2:1 and 1:1 showed tolerance to sulfur poison compared to the pure MnO_2_, while the presence of H_2_O caused a slight and reversible deactivation.^[^ ^302^ ^]^

A series of MnO*_x_* nanocatalysts doped with BaO were produced via a mechanochemical technic. The results indicated that the 10 wt% BaO–MnO*_x_* catalyst had the best catalytic performance, corresponding to 90% of CH_4_ conversion temperatures at 427 °C, which was attributed to the higher ability to supply oxygen through the components during combustion. In addition, all catalysts enhanced by BaO presented high stability without any obvious reduction in reactant conversion over time on stream, while pure MnO*_x_* catalyst lost about 5% during 900 min due to the sintering of the particles (Figure 16k,l). Furthermore, catalytic activity was affected by surface area, synergistic interaction between components, reducibility, and dispersion. Hence, increasing BaO content from 10 to 20 wt% reduced the catalytic activity because of the crystal size growth and the surface area reduction.^[^ ^303^ ^]^

#### 2.2.3 Nickel‐Based Catalysts

NiO is a promising transition metal oxide nanoparticle that could replace noble metal catalysts because of its excellent performance in activating C—H bonds.^[^ ^304^, ^305^ ^]^ Hence, interest in synthesizing various NiO nanomaterials for CH_4_ activation has grown. It was recently revealed that various polymorphous NiO nanomaterials produced via a one‐pot thermal decomposition technique were found to have varying lean CH_4_ combustion capabilities, with NiO nanoparticle‐based sheets exhibiting the highest catalytic activity for CH_4_ activation at 450 °C. The enhanced catalytic activity was ascribed to low crystallinity, porous structure, and a high specific surface area formed at low sintering temperatures, with basic nickel carbonate and nickel oxalate dihydrate as precursors.^[^ ^305^ ^]^ Furthermore, the soft templates method was used to prepare mesoporous NiO catalysts. NiO nanoparticles produced with ethylene glycol as a template (NiO‐PEG) may catalyze the complete oxidation of CH_4_ at 440 °C. By contrast, while the overall activity of NiO‐PVP (NiO catalysts prepared with polyvinyl pyrrolidone) is slightly lower than that of NiO‐PEG, NiO prepared with direct calcination demonstrates the lowest catalytic activity among all the catalysts, as shown by its T_100%_ of 550 °C.^[^ ^306^ ^]^ NiO catalysts synthesized by the hydrothermal template approach are more active due to large intraparticle and interparticle mesopores with different sizes, giving rise to catalysts with much higher surface areas and more mobile active oxygen species.^[^ ^307^ ^]^

**Figure 17:**
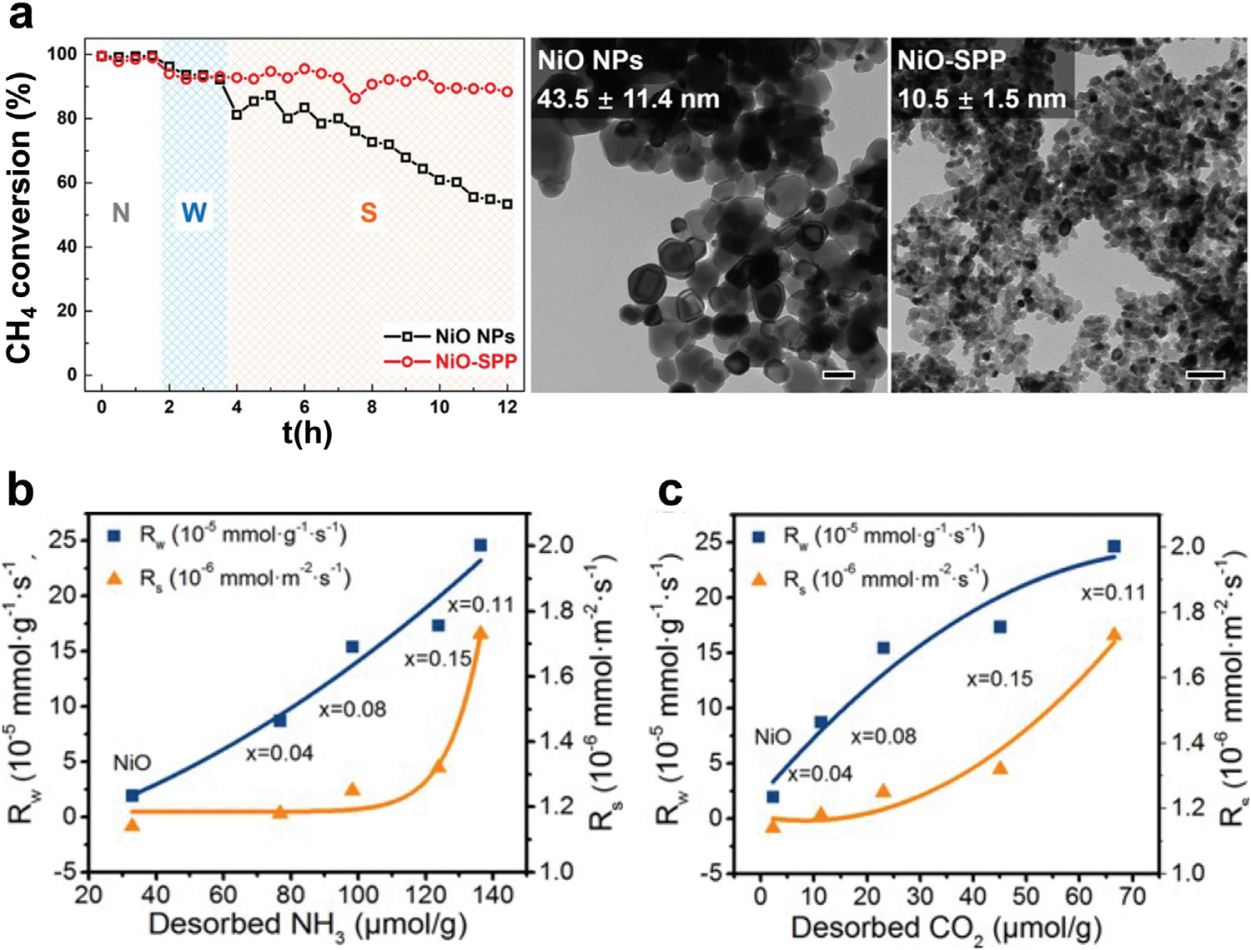
a) Stability tests of NiO NPs and NiO‐SPP with TEM images after the tests. Scale bar: 50 nm. (reaction temperature 500 °C; 0.2% CH_4_, 15% H_2_O, and 1 ppm of SO_2_. Reproduced with permission.^[^ ^309^ ^]^ Copyright 2021, American Chemistry Society. The relation between the CH_4_ conversion rates (reaction at 280 °C) over Ni1– *x*Zr*_x_* O_2_–*δ* nanocatalysts with desorbed the amount of b) NH_3_ and c) CO_2_. Reproduced with permission.^[^ ^323^ ^]^ Copyright 2021, American Chemistry Society.

In addition, Chen et al. recently reported a solid–liquid precipitation process to synthesize NiO nanomaterials using NH_3_·H_2_O as a precipitant.^[^ ^308^ ^]^ Compared to the liquid‐phase precipitation process, the solid–liquid precipitation process was simple and used less distilled water in the synthesis process, making it more economical and environmentally friendly. Moreover, the nanomaterials prepared by solid–liquid precipitation (NiO‐NSL) with a rod‐like nanostructure demonstrated good CH_4_ combustion activity and achieved complete CH_4_ combustion at 390 °C, whereas NiO‐NLL prepared by liquid‐phase precipitation completely oxidized CH_4_ at 440 °C in the same reaction conditions. Based on XPS and H_2_‐TPR results, the Ni^2+^ content on the NiO‐NSL was higher than that of NiO‐NLL, and the increased activity could be due to the presence of more Ni^2+^.^[^ ^308^ ^]^ Even if NiO has exhibited remarkable performance at low‐temperature, its sulfur resistance capacity is low.^[^ ^258^ ^]^ In addition, catalytic activity is limited due to its lower surface areas and porosities, limiting its practical application.^[^ ^306^ ^]^ Thus, sulfur‐resistant NiO nanocatalysts (NiO‐SPP) were synthesized by modifying NiO nanoparticles with surface polymeric phosphate (SPP); the NiO‐SPP catalyst exhibited better activity toward lean CH_4_ oxidation with T_90_ at 445 °C. In addition, it can significantly reduce the potential of active sites interacting with water and sulfur species, as shown in **Figure** **17**a. The exterior reason for the increased sulfur tolerance capacity is the protection of the surface structure of NiO‐SPP, while the internal cause is the slowing of the initial sulfation rate.^[^ ^309^ ^]^ Introducing an additional metal into NiO particles may also improve catalytic activity.^[^ ^310^ ^]^ The amount of Ni^3+^ or O^−^ species might increase if low‐valence metal cations were introduced into the NiO lattice.^[^ ^311^ ^]^ Meanwhile, metals with high valence might diminish Ni^3+^ or O^−^ species because of the reduced positive holes (p^+^), consistent with the principle of controlled valence.^[^ ^312^ ^]^ Given that Ni^2+^ species are essential for C–H activation, it makes sense to incorporate high‐valence dopants into NiO to prevent Ni^2+^ to Ni^3+^ transformation.^[^ ^304^ ^]^

It has been uncovered that incorporating Mg, CeO_2_, or Zr support also promoted CH_4_ oxidation over NiO‐based catalysts.^[^ ^304^, ^313^, ^314^, ^315^ ^]^ The density functional calculations found that some binary systems, such as Ni^2+^/CeO_2_(111), can even activate CH_4_ at room temperature due to metal–support interactions.^[^ ^316^, ^317^, ^318^, ^319^, ^320^ ^]^ Huang et al. prepared a series of NiO/CeO_2_ catalysts using a facile impregnation methodology.^[^ ^314^ ^]^ The results showed that increasing NiO content increased the catalytic activity of NiO/CeO_2_, with a T_90_ value of 467 °C for 10 wt‐% NiO/CeO_2_. However, when the NiO loading was augmented to 15% and 20%, no improvement was observed because the NiO nanoparticles aggregated, and the number of reactive sites did not increase. The presence of CeO_2_ reduced NiO aggregation, improved NiO reduction, and provided more oxygen species for CH_4_ combustion.^[^ ^314^, ^319^ ^]^ It is worth mentioning that 10 wt% NiO/CeO_2_ catalyst declined slightly with the addition of 3.1% water vapor with T_90_ of 480 °C. Meanwhile, CH_4_ was oxidized to CO_2_ and H_2_O. Thus, 10 wt% NiO/CeO_2_ catalyst showed a degree of water tolerance and stability.^[^ ^314^ ^]^ The DFT calculations attributed the stability of NiO/CeO_2_ catalyst to the facile elimination of cokes, which are consumed mainly by interfacial oxygen.^[^ ^321^ ^]^

The ZrO_2_‐supported NiO catalysts (NiO/ZrO_2_) and ZrO_2_‐based binary oxides (NiO‐ZrO_2_) have been synthesized for CH_4_ oxidation, and it was found that NiO species acted as active sites, while the ZrO_2_ support affected the active area.^[^ ^322^ ^]^ Wang et al. suggested the synthesis of highly effective NiZr nanocatalysts by doping NiO with zirconium using a simple sodium carbonate‐assisted coprecipitation method. The optimized inclusion of Zr into the NiO lattice supplied the Ni_0.89_Zr_0.11_O_2−*δ*_ nanocatalyst with a small crystallite size, large specific surface area, and at the same time, increased surface acidic–basic sites. Furthermore, due to the enhanced conversion of Ni^3+^ to Ni^2+^, numerous active Ni^2+^, and surface oxygen species were produced, which played critical roles in the adsorption/activation of CH_4_. In addition, the catalytic activity of nanocatalysts correlated well with the amount of surface acidic−basic sites, as shown in Figure 17b,c. As a result, Ni_0.89_Zr_0.11_O_2−*δ*_ demonstrated outstanding low‐temperature activity with a T_90_ at 405 °C in water vapor, high CO_2_ selectivity, and enhanced catalytic stability with or without water.^[^ ^323^ ^]^ Given that the increased Ni^2+^ species were the cause of the enhanced catalytic activity, Ni^2+^–O^2−^ pair was identified as the active site. CH_4_ adsorbed on Ni^2+^–O^2−^ active underwent dissociation to make —CH_3_ and —OH, then —CH_3_ was oxidized to formate and carbonate intermediates. After that, the carbonate species form CO_2_ to complete the reaction.

Additionally, mixed oxide systems of copper, which naturally contain a lot of extranuclear d‐shell electrons, have been created and employed in catalytic processes.^[^ ^324^ ^]^ Given the similarity in atomic size and electronegativity between copper and nickel, Fan et al. recently introduced copper to the NiO lattice to form Cu–Ni solid solution. This resulted in more unsaturated nickel atoms and smaller NiO nanoparticle sizes, leading to more lattice defects. Also, the oxygen‐binding energy was dramatically reduced because of formation of Ni—O—Cu bond and the interaction between CuO and NiO. As a result, NiCu binary oxides outperformed single metal oxides in CH_4_ oxidation. For instance, 9Ni1Cu displayed the lowest temperature of T_90_ = 410 °C CH_4_ conversion, which was reduced by 60 °C compared to NiO. At the same time, 9Ni1Cu demonstrated the most negligible activity loss in activity in the presence of water among the investigated samples while still producing the lowest T_90_ and *E*_a_ values.^[^ ^310^ ^]^

#### 2.2.4 Other Transition Metal‐Based Catalysts

Recently, iron oxides have shown promise as active catalysts for CH_4_ conversion reactions.^[^ ^325^ ^]^ However, their potential in CH_4_ conversion via heterogeneous catalysis has been relatively limited to partial oxidation reactions such as methanol or formaldehyde.^[^ ^326^ ^]^ The complete oxidation route of CH_4_ is studied to a much lesser extent. Recently, He et al. reported hematite (*α*‐Fe_2_O_3_) as a highly efficient catalyst for the complete oxidation of CH_4_ with excellent stable performance below 500 °C with a 100% selectivity to CO_2_. With low apparent activation energy of 17.60 kcal mol^−1^, the reported activity was remarkable and comparable to that of precious metal‐based catalysts. According to a theoretical analysis, the high performance is caused by a tetra‐iron center with an antiferromagnetically connected iron dimer on the hematite surface (110), similar to the methanotroph enzyme CH_4_ monooxygenase that activates CH_4_ under ambient conditions in nature.^[^ ^31^ ^]^ In general, the performance of transition metal oxide‐based catalysts for CH_4_ activation was associated with higher surface area, metal loading, smaller crystalline size, more surface oxygen vacancies, better low‐temperature reducibility, structural defects, and highly exposed active sites. Notably, the performance of transition metal‐based catalysts for CH_4_ activation depends on different factors, including catalyst preparation methods that affect catalyst morphology, texture properties, surface chemistry, and reaction conditions. Much has been done to reduce the activation temperature of CH_4_ with transition metal‐based catalysts, as summarized in Table 4. However, to meet the needs of the industry, researchers must reduce the gap between laboratory‐scale experiments and the application of the prepared catalyst in full‐scale systems.

## 3 Catalytic Activation of Methane at Relatively Low Temperature

### 3.1 Thermocatalysis for Partial Oxidation of Methane in Liquid‐Phase

The gas‐phase partial oxidation of CH_4_ has been reported using N_2_O, O_2_, and H_2_O as oxidants.^[^ ^330^, ^331^, ^332^, ^333^, ^334^, ^335^, ^336^, ^337^ ^]^ However, most reactions can only proceed at relatively high temperatures. For instance, recently, Yamasaki et al. reported the selective conversion of CH_4_ by applying higher concentrations of reactants. The results showed that CH_4_ was activated using NO as an oxidant in conjunction with a Pt/Al_2_O_3_ catalyst to produce HCN even at 300 °C. However, CH_4_ and NO conversion values were very low, and small amounts of HCN and NH_3_ were generated along with the formation of CO_2_, N_2_O, and N_2_.^[^ ^338^ ^]^ In addition, a broadened temperature window and enhanced CO_2_ selectivity were achieved by combining CH_4_ and propane as the coreductant. The mixtures with propane/methane of 1/2 showed the most superior T_90_ at 500 °C temperatures for NO*_x_* reduction over the In/H‐BEA catalyst. It was claimed that CH_4_ could be activated on In_2_O_3_ sites to generate oxygenates, reacting with nitrate to produce N_2_.^[^ ^339^ ^]^ Thus, the mild temperature conditions for the direct route of partial oxidation of CH_4_ to oxygenate products, especially methanol, are desirable for efficient natural gas reserves.

**Figure 18:**
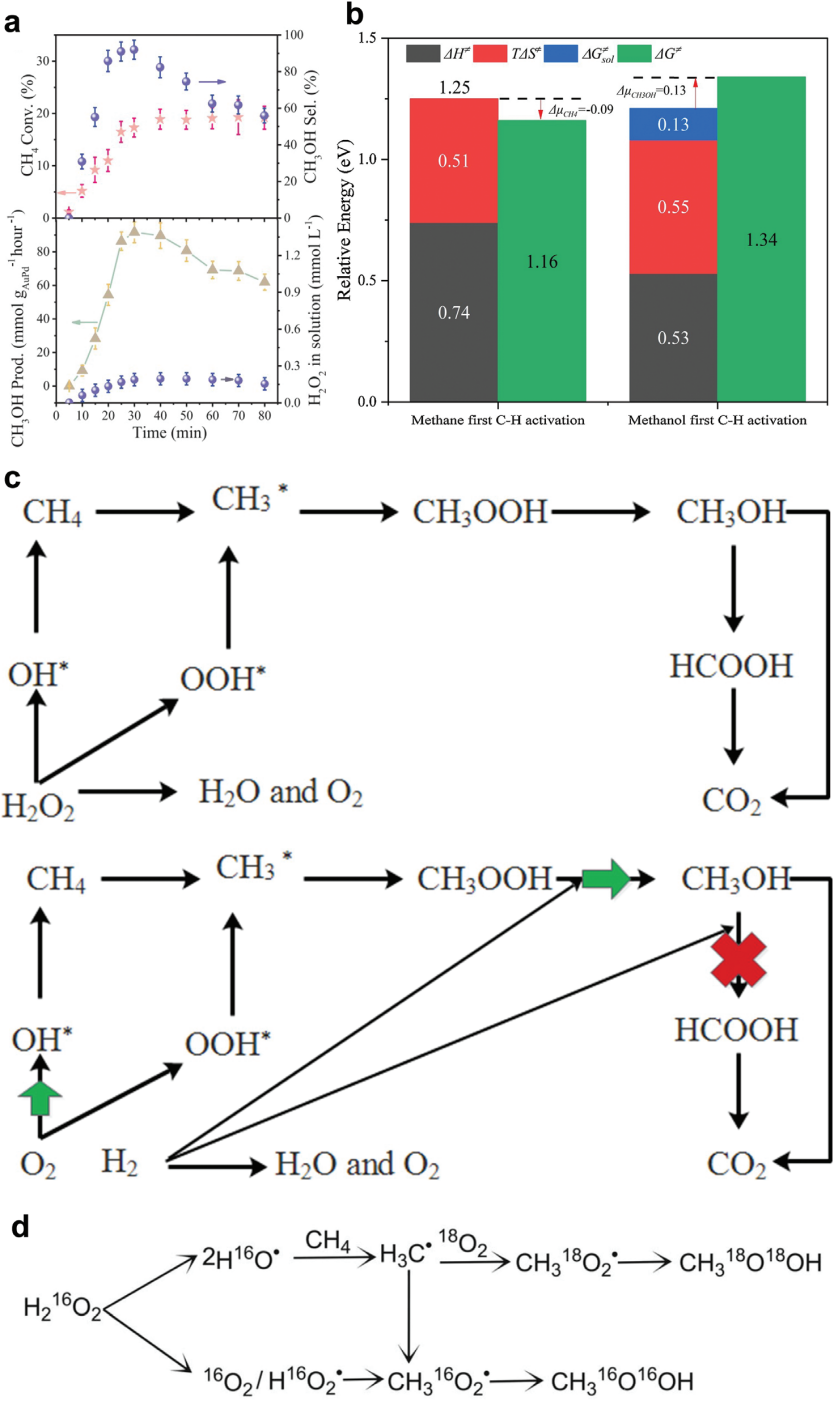
a) The effects of reaction time on CH_4_ conversion (Conv.), CH_3_OH selectivity (Sel.), CH_3_OH productivity (Prod.), and H_2_O_2_ concentration in water solution over AuPd@ZSM‐5 catalysts. Reproduced with permission.^[^ ^340^ ^]^ Copyright 2020, AAAS. b) Activation free energies Δ*G*′ = of C—H bond breaking of CH_4_ and CH_3_OH. Reproduced with permission.^[^ ^367^ ^]^ Copyright 2021, Elsevier. c) Reaction pathway of direct CH_4_ to CH_3_OH using H_2_O_2_ (a) or O_2_/H_2_ mixture gases oxidants. Reproduced with permission.^[^ ^371^ ^]^ Copyright 2020, Elsevier. d) Proposed mechanism for CH_4_ oxidation in the presence of H_2_O_2_ and molecular O_2_. Reproduced with permission.^[^ ^369^ ^]^ Copyright 2017, AAAS.

Many attempts have been made to improve the conversion and selectivity of CH_4_ conversion reaction systems. H_2_O_2_ is a clean and efficient oxidant for CH_4_ oxidation in the liquid‐phase system (**Figure** **18**a) under mild conditions,^[^ ^340^, ^341^ ^]^ unlike H_2_SO_4_ and trifluoroacetic acid.^[^ ^342^, ^343^ ^]^ Thus, H_2_O_2_ has been used as the benign oxidant in homogeneous and heterogeneous catalytic systems. Since Shilov et al. discovered that CH_4_ could be directly converted to CH_3_OH at 120 °C by employing Pt^II^ as the C–H activation, the partial oxidation of CH_4_ in a homogeneous system has garnered much attention.^[^ ^344^, ^345^ ^]^ Generally, ionic liquids (ILs) were reported to have much higher CH_4_ solubility than aqueous and organic solvents, especially for ILs containing C–F anions.^[^ ^346^ ^]^ In the H_2_O_2_/[Eim][NTf_2_] system at 90 °C, Huang et al. discovered that AuCl_3_ (phenanthroline) was treated with a suitable stoichiometric amount of silver trifluoromethanesulfonate (AgOTf) was capable of catalyzing CH_4_ oxidation directly to CH_3_OH as the principal product.^[^ ^347^ ^]^ Significant progress has also been made for CH_4_ partial oxidation in heterogeneous catalytic systems, and the currently reported catalysts for CH_4_ oxidation by H_2_O_2_ are mainly based on scarce noble metals, such as Au, Pd, Ru, Rh, and Ir.^[^ ^238^, ^348^, ^349^, ^350^ ^]^ For example, hydrophobically modified AuPd@zeolite activated CH_4_ C—H at 70 °C using in situ generated H_2_O_2_ from H_2_ and O_2_,^[^ ^340^ ^]^ while Pd_1_O_4_ single sites attached on the internal surface of microporous silicate transformed CH_4_ to CH_3_OH at 50 °C with H_2_O_2_.^[^ ^351^ ^]^ In addition, Au–Pd alloy catalysts have been proven to transform CH_4_ to methanol with a high CH_4_ conversion ratio and methanol selectivity at a mild temperature.^[^ ^352^, ^353^ ^]^ It was suggested that H_2_O_2_ as an oxidant is the crucial factor in activating CH_4_ and that the •OH radical derived from H_2_O_2_ attacked one C—H bond in CH_4_ to produce the •CH_3_ radical.^[^ ^353^, ^354^ ^]^ Furthermore, it was claimed that the catalyst's structure had an impact on the radical generation and H_2_O_2_ degradation, two processes that are directly related to CH_4_ conversion and methanol selectivity. The interface between TiO2 support and AuPd nanoparticles promotes H_2_O_2_ degradation, lowering CH_4_ conversion and methanol selectivity.^[^ ^354^ ^]^ In addition, optimal CH_4_ adsorption capacity and the electronic synergy effect for Au–Pd alloy were critical parameters for enhancing CH_4_ conversion and methanol selectivity.^[^ ^355^ ^]^ Moreover, the ability of the porous framework to adsorb CH_4_ and the active isolation center was suggested to promote the partial oxidize CH_4_ to methyl trifluoroacetate at 80 °C with H_2_O_2_ as oxidant.^[^ ^39^ ^]^

Early transition metal oxide clusters comprised of a few metal and oxygen atoms have been shown to activate CH_4_ at room temperature.^[^ ^356^, ^357^, ^358^ ^]^ Several metals–oxo clusters have been synthesized with zeolites for the C—H activation in CH_4_.^[^ ^359^ ^]^ Cu–oxo clusters have received much attention for CH_4_ activation because they mimic copper‐containing monooxygenase's catalytic activity.^[^ ^360^ ^]^ For example, the active site for oxidation of CH_4_ to methanol on O_2_‐activated Cu‐exchanged ZSM‐5 at 125 °C has been identified as the dinuclear [Cu(O)Cu]^2+^ cluster.^[^ ^361^, ^362^ ^]^ Under mild reaction conditions, Maeno et al. supported In–oxo clusters on zeolite for CH_4_ activation. The results showed that the activation of In‐exchanged CHA zeolites via reductive solid‐state ion exchange resulted in multinuclear In–oxo clusters (InO*_x_*‐CHA). CH_4_ was also more likely to adsorb and react with dinuclear In–oxo ions than monomeric In–oxo ions.^[^ ^46^, ^363^ ^]^ Furthermore, single iron atoms have been shown to be more effective due to Fe–O active centers, which may easily break C—H bonds and catalyze subsequent CH_4_ oxidation to formic acid through accessible radical pathways under mild conditions.^[^ ^364^, ^365^ ^]^ For example, Cui et al. demonstrated that at normal temperature (25 °C), H_2_O_2_ could directly oxidize CH_4_ to C1 oxygenates (CH_3_OH, CH_3_OOH, HOCH_2_OOH, and HCOOH) over graphene‐confined single‐atom Fe (FeN_4_) catalysts. In addition, according to DFT calculations and electron paramagnetic resonance studies, the unique O–FeN_4_–O structure was the active site for CH_4_ oxidation along a radical pathway to yield CH_3_OH and CH_3_OOH with a low barrier of 0.79 eV. The produced CH_3_OH is then oxidized at room temperature into HOCH_2_OOH and HCOOH.^[^ ^364^ ^]^ In addition, using DFT simulations, Tan et al. demonstrated that C vacancies and P dopants close to FeN_4_ centers could considerably enhance the catalytic performance of FeN_4_ catalysts, which can boost the catalytic performance of FeN_4_ catalysts for room temperature CH_4_ conversion.^[^ ^366^ ^]^

Furthermore, Guo et al. identified Cu_1_–O_4_ entity on ZSM‐5 as an active site, and Cu_1_/ZSM‐5 single‐atom catalyst activated CH_4_ with 99% selective toward oxygenates products at 50 °C with H_2_O_2_ in an aqueous medium. Their findings affirm that each isolated Cu atom stabilized by four O moieties on the ZSM‐5 support possesses a uniform Cu_1_–O_4_ entity as an active site and predominantly activates CH_4_ rather than CH_3_OH, as shown in Figure 18b, which is desirable for the preparation of highly selective C1 oxygenates, particularly methanol.^[^ ^367^ ^]^ Although zeolites as support have been used in the partial oxidation of CH_4_ to CH_3_OH, the main limitation of zeolite is the strong adsorption of methanol due to the hydrophilicity of the zeolite surface. Thus, Imyen et al. report an alternative concept for CH_4_ utilization to produce methanol by using the Fe‐zeolite@metal–organic framework (MOF) as a dual functional material in which MOF and Fe‐zeolite act as a gas adsorbent and catalyst, respectively.^[^ ^368^ ^]^ CH_4_ was initially adsorbed on ZIF‐8 at 50 °C and converted to methanol on Fe‐zeolite at moderate temperatures. Moreover, the enhanced surface hydrophobicity of zeolite by MOF deposition weakened the interaction between the produced methanol and catalyst surfaces, eventually facilitating methanol desorption.

Even if H_2_O_2_ has been seen as a good oxidant for CH_4_ activation at a very mild temperature,^[^ ^7^, ^369^, ^370^ ^]^ its high cost and limited oxygen usage efficiency prevent it from being widely commercialized and industrialized. Researchers are proposing novel oxidants with outstanding performance but low cost under very mild reaction conditions. Recently, it was shown that utilizing oxygen and hydrogen mixture gas as the natural and cost‐effective oxidant; carbon materials supported Pd–Au nanoparticles catalyst allowed high methanol selectivity, as shown in Figure 18c.^[^ ^371^ ^]^ Compared to H_2_O_2_, oxygen and hydrogen mixed gas improves CH_4_ conversion and raises the selectivity of methanol in all oxidation products. Carbon nanotubes (CNTs) supported Pd–Au nanoparticles, providing good catalytic activity in turning CH_4_ to CH_3_OH at 50 °C in an aqueous phase.^[^ ^372^ ^]^ In addition, the loading amount of Pd–Au nanoparticles affects the CH_4_ activation ability, as recently revealed by He et al., who investigated the crucial roles of the physical and chemical properties of Pd–Au nanoparticles. Compared to lower metal content catalysts, the 2.5% Pd‐2.5% Au nanoparticles supported by CNTs catalyst produced more active and stable Pd–Au nanoparticles. Moreover, the Pd bivalent state was the most active Pd metallic state in the Pd–Au nanoparticle catalyst.^[^ ^371^ ^]^ It is worth mentioning that the oxidation of CH_4_ with oxygen (O_2_) isotope in the presence of hydrogen peroxide (H_2_O_2_) under colloidal gold–palladium nanoparticles resulted in the production of methanol with selectivity (92%) in an aqueous solution at mild temperatures following the reaction mechanism shown in Figure 18d.^[^ ^369^ ^]^

### 3.2 Photocatalytic Activation of Methane

Photocatalysis uses photons instead of thermal energy to drive many harsh reactions process under mostly ambient conditions. Thus, CH_4_ photocatalytic activation is considered more environmentally friendly, sustainable, and renewable technology.^[^ ^373^, ^374^, ^375^, ^376^ ^]^ In addition, photocatalysis can overcome catalyst deactivation caused by extreme reaction conditions like high temperatures. However, most published works presented low activity and selectivity that limited the photocatalytic route's wide‐scale applications.^[^ ^377^ ^]^ In addition, most previous review works recommended more studies about material design and the activation mechanism of the C—H bond, the generation and migration of reactive free radical species, and reactions between •CH_3_ and electrophilic reactants, as there are essential to reach a feasible CH_4_ conversion.^[^ ^24^, ^25^, ^341^, ^378^, ^379^, ^380^, ^381^ ^]^

**Figure 19:**
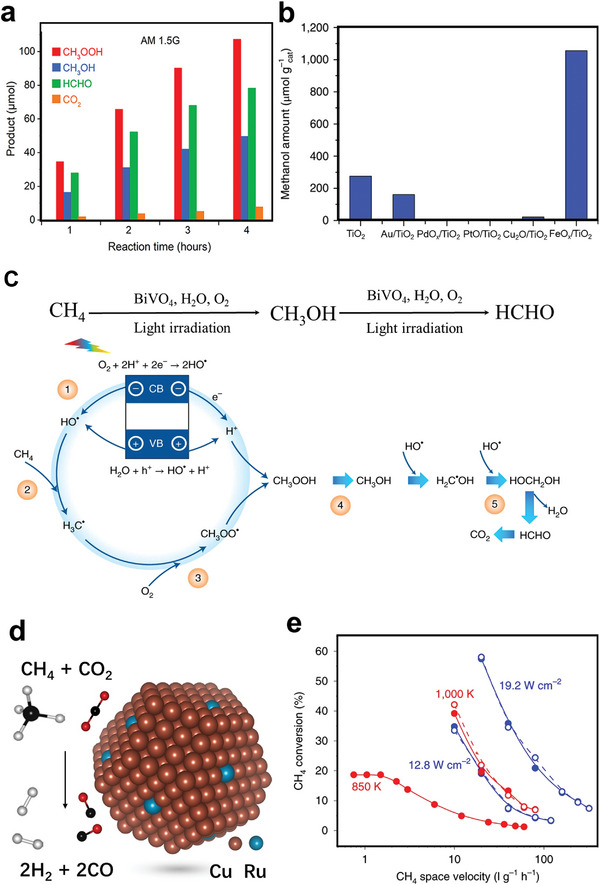
a) Time course of product yields under simulated sunlight irradiation with 0.1 wt% Au/ZnO at room temperature, light source: solar simulator (AM 1.5G), and light intensity 100 mW cm^−2^. Reproduced with permission.^[^ ^382^ ^]^ Copyright 2019, American Chemistry Society. Methanol yields b) for a series of metal‐modified TiO_2_ samples after 3 h of full arc irradiation. Reproduced with permission.^[^ ^389^ ^]^ Copyright 2018, Springer Nature. c) Reaction mechanism for photooxidation of CH_4_ using quantum‐sized bismuth vanadate. Reproduced with permission.^[^ ^396^ ^]^ Copyright 2021, Springer Nature. d) Schematic of a Cu–single‐atom Ru surface alloy catalyst with the dry reforming reactants and products shown on the left. e) CH_4_ conversion by photocatalysis (blue) and thermocatalysis (red). Filled and unfilled circles are two different batches of measurements, while dashed and solid lines are visual guides. Reproduced with permission.^[^ ^400^ ^]^ Copyright 2020, Springer Nature.

Typical, stable oxide semiconductors are generally used to produce highly energetic oxygen species, such as O_2_^−^ and •OH radicals, resulting in the oxidation of CH_4_ into HCHO, CH_2_OH, CO_2_, or CO as the main products^[^ ^381^, ^382^, ^383^, ^384^, ^385^ ^]^ as shown in **Figure** **19**a. When a semiconductor is activated with a light source, electron–hole pairs are created that have kinetic energy equal to the bandgap value of the semiconductor. While the holes migrate to the valence band and carry out oxidation, the electrons move to the conduction band and carry out reduction.^[^ ^386^ ^]^ Thus, the photoenergy overcomes the activation barriers by breaking the thermodynamic equilibrium in an endothermic reaction and offers the possibility to activate CH_4_ at room temperature.^[^ ^383^, ^387^ ^]^ It is worth noting that mercury lamps and xenon lamps are the most sources of light used to provide ultraviolet or visible light, respectively.^[^ ^388^ ^]^ In addition, few studies used lasers.^[^ ^389^, ^390^ ^]^ However, results suggested that adding hydroxyl radicals decreases methanol production in the system due to laser irradiation that emits a high flux density monochromatic light.^[^ ^391^ ^]^ Recently, aqueous‐phase photocatalysis was explored to generate liquid oxygenate products such as methanol, formic acid, and formaldehyde using H_2_O_2_ at room temperature.^[^ ^392^ ^]^ However, the generated methanol tends to be overoxidized into CO or CO_2_.^[^ ^373^ ^]^ Therefore, achieving highly selective CH_4_ oxidation with O_2_ into methanol is essential but challenging under ambient conditions.^[^ ^393^ ^]^ The iron oxide species were dispersed onto TiO_2_ (FeO*_x_*/TiO_2_) using a highly repeatable impregnation technique, which was then used to oxidize CH_4_ to CH_3_OH under moderate light irradiation and ambient conditions with oxidant H_2_O_2_. The optimized sample contains both FeOOH and Fe_2_O_3_ active sites and produces nearly four times as much methanol as bare TiO_2_, with a 15% conversion rate for CH_4_ and 97% selectivity, as shown in Figure 19b. Based on the reaction mechanism study, the superior catalytic activity of FeO*_x_*/TiO_2_ is ascribed to the efficient electron transfer from TiO_2_ to iron species.^[^ ^389^ ^]^

As the important point to selectively generate CH_3_OH is hydrogen detachment from CH_4_, •OH radical is impressively accountable for the dehydrogenation of CH_4_ to make •CH_3_ radical,^[^ ^394^, ^395^, ^396^ ^]^ as shown in step 2 of the reaction mechanism in Figure 19c. Thereby, cocatalyst‐H_2_O_2_ oxidant system plays an essential role in determining CH_3_OH generation.^[^ ^397^ ^]^ Importantly, due to its high electronegativity and the dehydrogenation of CH_4_ to create the methyl (•CH_3_) radicals that Au prefers, Au is extraordinarily active in the selective oxidation of hydrocarbons.^[^ ^398^, ^399^ ^]^ Additionally, atomic‐scale metals contribute to distinct catalytic behavior from their bulk or nanoparticle counterparts due to their maximal active sites.^[^ ^400^, ^401^ ^]^ Therefore, the preparation of single atom is a potentially effective way to activate CH_4_ during the photocatalytic process (Figure 19d). For instance, a plasmonic photocatalyst of a Cu nanoparticle and Ru single atom gave high light energy efficiency with higher conversion than thermocatalysis at 727 °C, as shown in Figure 19e. Through a simple photochemical reduction method at room temperature, Zeng et al. dispersed atomic gold on tungsten trioxide (Au_1_/WO_3_). The Au_1_/WO_3_ material shows a specific electronic structure and tip‐enhanced local electric field advantageous for activating CH_4_ under visible light at room temperature. The theoretical simulations show that the resultant methanol has lower adsorption energy on Au_1_ than Au particles, reducing the overoxidation of methanol and increasing its selectivity.^[^ ^402^ ^]^ Thus, it is suggested that the combination of single atoms and clusters might also be attractive for CH_4_ oxidation.^[^ ^403^ ^]^

In addition, previous studies have used black phosphorus (BP) nanosheets to support photocatalysis that allows broadband solar absorption.^[^ ^404^, ^405^ ^]^ In the recent work of Luo et al., Au single atoms were supported on BP.^[^ ^373^ ^]^ The produced catalyst (Au_1_/BP) was used to achieve mild oxidation of CH_4_ into methanol with the assistance of water and light irradiation with methanol selectivity above 99%. The mechanistic analysis under light irradiation revealed that water helped activate O_2_ to form reactive hydroxyl groups and •OH radicals. The reactive hydroxyl groups allowed for the mild CH_4_ oxidation into CH_3_* species, followed by oxidation of CH_3_* via •OH radicals into methanol.^[^ ^373^ ^]^ Furthermore, graphitic carbon nitride (g‐CN) has shown intriguing performance for a series of photocatalytic reactions.^[^ ^388^ ^]^ However, the band structure of g‐CN does not possess the sufficient oxidizing potential for the generation of radical hydroxyl activation of CH_4_.^[^ ^406^ ^]^ Thus, Shi et al. synthesized mesoporous metal‐free g‐CN material through the polymerization of urea and applied it for partial oxidation of CH_4_ to methanol at 35 °C in the presence of hydrogen peroxide (H_2_O_2_). The addition of g‐CN photocatalyst enhanced CH_4_ activation under visible light by ten times the production of methanol threefold than when WO_3_ was used. Interestingly, the amount of CH_4_ in water was an essential limiting factor for CH_4_ conversion, indicating that once CH_4_ increased, more radicals could be consumed for CH_3_OH generation. More essentially, mechanism studies revealed that besides •OH, both •O_2_^−^ and h^+^ were important for the final production of methanol. More importantly, the isotope tracer confirmed that the methanol product's carbon atoms are from CH_4_ rather than any possible contaminants on the catalyst surface.^[^ ^392^ ^]^

Although CH_4_ conversion efficiency is one important index to evaluate the catalytic activity, most studies focus on selectivity and product yields, and few have reported photocatalytic efficiency. In addition, most photocatalysts have shown much lower conversion efficiency, some even less than 10%.^[^ ^384^, ^407^ ^]^ A high CH_4_ selectivity is less attractive if a high conversion cannot be achieved; therefore, a significant improvement is needed to balance the product selectivity and CH_4_ conversion efficiency. Moreover, the coexistence of multiple reaction products increases the cost and energy of product separation. Moreover, a novel technology known as photothermal catalysis, due to its advantage of overcoming thermocatalysis and photocatalysis drawbacks, can be explored to improve the low activity of the CH_4_ photocatalytic process.^[^ ^380^ ^]^ In addition to inorganic materials, black phosphorus, and graphitic carbon nitride, covalent triazine and covalent organic frameworks can also demonstrate the usability for CH_4_ photocatalysis due to their light absorption properties, band structure, and surface characteristics, which can be manipulated using molecular engineering.^[^ ^408^ ^]^ Recently, An et al., inspired by CH_4_ monooxygenase in nature, immobilized mono‐iron hydroxyl sites on a metal–organic framework to synthesize PMOF‐RuFe(OH) photocatalyst. PMOF‐RuFe(OH) exhibited 100% CH_3_OH selectivity with a time yield of 8.81 mmol g_cat_^−1^ h^−1^ versus 5.05 mmol g_cat_^−1^ h^−1^ for CH_4_ monooxygenase under ambient conditions in the presence of H_2_O and O_2_.^[^ ^375^ ^]^ Furthermore, cokes formation or durability could be an issue in photocatalysis with long‐term runs.^[^ ^409^ ^]^ Hence, encapsulated photocatalysts might help in developing durable photocatalysts. For instance, 0.1 wt% Au/ZnO photocatalyst reported by Song et al. showed 3 ppm of Zn^2+^ in the solution under dark condition; however, after 2 h under light Zn^2+^ concentration increased to 10 ppm, which suggest that ZnO was photocorroded.^[^ ^382^ ^]^ The poor understanding of the reaction mechanism is still a challenge due to the short lifetime of free radicals generated during the reaction.^[^ ^377^ ^]^ Therefore, it was recommended to use complementary time‐resolved spectroscopies in cooperation (situ spectroscopies, operando characterization, and vacuum ultraviolet soft photoionization molecular‐ beam mass spectrometry) rather than one technique alone.^[^ ^23^ ^]^ Overall, photocatalysis can offer a promising approach for CH_4_ conversion with the advantages of low energy consumption, simple reaction pathways, and the direct conversion of CH_4_ into desired products.

### 3.3 Electrocatalytic Activation of Methane

With the availability of renewable energy, electrocatalysts have appeared as an encouraging method for the partial oxidation of CH_4_ to produce oxygenate products. The reaction system requires a power source, cathode, and anode separated by ion‐exchange electrolytes and membranes.^[^ ^27^ ^]^ Different reviews have been recently reported, focusing on the direct and indirect electrochemical CH_4_ activation,^[^ ^410^, ^411^ ^]^ electrocatalyst structures,^[^ ^28^ ^]^ electrolytes,^[^ ^411^ ^]^ mechanistic understanding, reaction engineering,^[^ ^412^ ^]^ and theoretical modeling.^[^ ^413^ ^]^ One of the primary benefits of electrocatalysis is the variety of ways to activate CH_4_ at room temperature by avoiding the use of strong oxidants such as H_2_O_2_, which has the potential to reduce operating costs compared to thermal catalytic systems. In addition, the electrocatalyst's selectivity and activity can be adjusted by varying the voltage, reaction temperature, catalysts, applied current, electrode material, oxygen source control, and gas‐feeding. Moreover, several other variables, such as electrolyte and pressure, can influence the electrochemical oxidation of CH_4_ and the final reaction product.^[^ ^412^ ^]^ Nevertheless, the reaction is highly challenging as CH_4_ oxidation to methanol is less favorable than the complete oxidation to CO_2_ due to its lowest standard potential.^[^ ^410^ ^]^ Therefore, the conversion rate suffers from low‐performance catalysts, which requires a basic understanding of the active sites.^[^ ^411^, ^413^, ^414^ ^]^ A critical step is forming adsorbed oxygen species (*O) that is vital for oxygen evolution side reaction.^[^ ^415^ ^]^ The oxygen species attack the adsorbed CH_4_ to break C—H, followed by electron and proton transfer.^[^ ^416^ ^]^ It was reported that high selectivity is achievable at a low current density; however, it decreases significantly as current density increases due to overoxidation of the products and the competitive reaction with oxygen evolution reaction. Therefore, it is suggested to focus on electrocatalysts with low Gibbs free energy for CH_4_ activation and high Gibbs free energy for water oxidation, such as V_2_O_5_, TiO_2_, PtO_2_, and RhO_2_.^[^ ^27^, ^417^, ^418^ ^]^ Moreover, substantial issues exist in separating products from electrolytes.^[^ ^28^ ^]^ Furthermore, there is still a problem associated with poor mass transfer and low solubility of CH_4_ for membrane electrode assemblies.^[^ ^419^ ^]^

**Figure 20:**
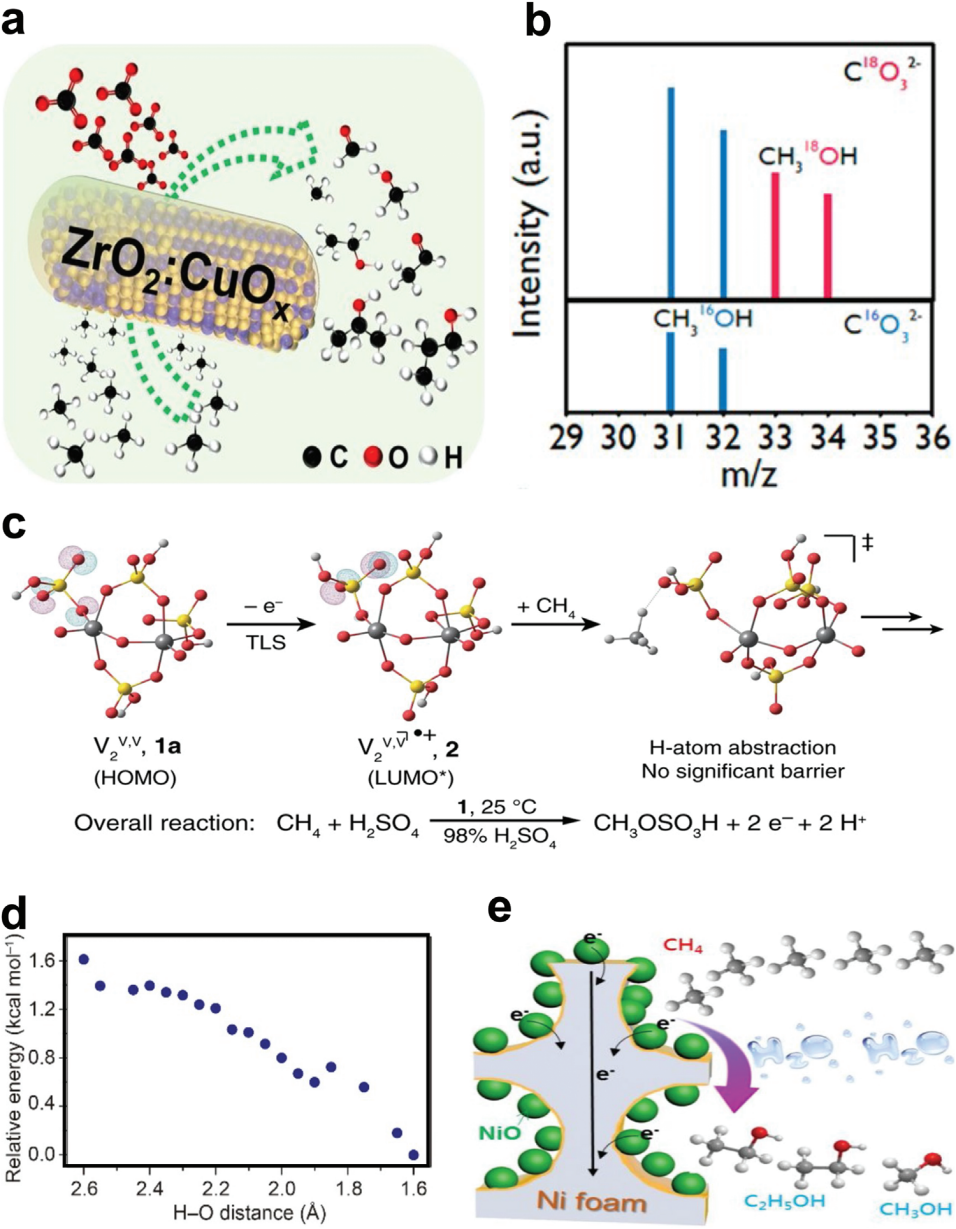
a) CH_4_ electrochemical oxidation by ZrO_2_:CuO*_x_* hybrid catalyst. Reproduced with permission.^[^ ^422^ ^]^ Copyright 2020, Elsevier. b) MS for CH_3_OH from an electrolyte containing ^18^CO_3_^2−^ (top panel). The CH_3_OH contains ^18^O and ^16^O calculated as 38% and 62%, respectively. Typical MS for CH_3_OH (bottom panel). Reproduced with permission.^[^ ^426^ ^]^ Copyright 2021, American Chemistry Society. c) The structural information of the catalyst and a proposed mechanism d) a potential energy surface.^[^ ^418^ ^]^ e) Illustration of electrooxidation of CH_4_ to ethanol and methanol on the NiO/Ni interface at ambient temperature. Reproduced with permission.^[^ ^415^ ^]^ Copyright 2018, American Chemistry Society.

By combining electrochemical experiments with quantum chemical calculations, Prajapati et al. studied the relation between the activity of CH_4_ oxidation reaction and the binding energy of CH_4_ to identify the noncompetitive active sites of transition metal oxides (TMOs) electrocatalysts and determine reaction pathways to direct partial oxidation of CH_4_ to CH_3_OH. Their results revealed that the electrochemical oxidation of CH_4_ on TMOs proceeds with the physical adsorption of CH_4_ followed by the activation of C—H bonds forming either CH*_x_* intermediates or oxygenated intermediates to yield products such as CO_2_, CO, CH_3_OH, or HCHO. Out of the 12 TMOs studied, the experimental measurements showed only TiO_2_, IrO_2_, PbO_2_, and PtO_2_ as the active electrocatalysts for CH_4_ oxidation. The inactivity of ZrO_2_ and SnO_2_ was due to the poor electrical conductivity and lower population of active sites, respectively. In addition, bimetallic with 10% Cu_2_O_3_ on TiO_2_ increased the desorption of ^*^CH_3_OH by providing *OH and preventing overoxidation of CH_3_OH.^[^ ^420^ ^]^ In addition, various electrocatalysts of metal oxide such as NiO, Co_3_O_4_, V_2_O_5_,^[^ ^415^, ^418^, ^421^, ^422^, ^423^ ^]^ metal alloys,^[^ ^424^ ^]^ and noble metal^[^ ^425^ ^]^ have been studied in electrochemical CH_4_ oxidation, which concentrated on producing active oxygen species, or the adsorption of CH_4_. For instance, Xu et al. reported the capsule‐like ZrO_2_:CuO*_x_* bimetallic electrocatalyst by the hydrothermal method (**Figure** **20**a) used in CH_4_ electrochemical oxidation at room temperature. The experimental results illustrated the significant synergistic interaction of CuO*_x_* and ZrO_2_, whose distinct catalytic structure and electron transfer route were responsible for enhancing electrochemical activity. In addition, DFT studies revealed a pronounced charge redistribution after the ZrO_2_ supported the Cu_2_O(111), which enhanced the CH_4_ dissociation and formed a quick electron transfer network.^[^ ^422^ ^]^ Oh et al. also used carbonate as the oxidant provider by supporting it to ZrO_2_ NT through the hydrothermal method (ZrO_2_ NT/Co_3_O_4_). The Co_3_O_4_ nanoparticles generated on the ZrO_2_ nanotubes’ outer surface provided an accessible diffusion channel for CH_4_ gas, causing a low onset potential for electrochemical CH_4_ activation. This nanostructure engineering technique helps to improve catalytic activity for electrochemical CH_4_ oxidation by producing higher alcohols, as well as promoting the mass transfer of CH_4_, which could give researchers a new catalyst synthesis strategy.^[^ ^423^ ^]^ Indeed, the isotope data confirmed that CH_4_ could be activated by active oxygen on the catalyst surface provided from CO_3_^2−^ to form CH_3_OH, as shown in Figure 20b.^[^ ^426^ ^]^ In addition, ZrO_2_:NiCo_2_O_4_ quasi‐solid solution catalyst is an efficient electrocatalyst for CH_4_ oxidation with a carbonate solution as the electrolyte under an ambient environment.^[^ ^427^ ^]^ Furthermore, Deng et al. functionalized CH_4_ via electrochemical oxidation of a vanadium (V)‐oxo dimer to methyl bisulfate (CH_3_OSO_3_H) at room temperature, and the results showed low activation energy (10.8 kcal mol^‒1^). Indeed, DFT calculations revealed a reaction trajectory without significant energy barrier, as shown in Figure 20c,d.^[^ ^418^ ^]^

By using an aqueous‐phase precipitation strategy, similar to CH_4_ electrooxidation in 0.1 m Na_2_CO_3_ aqueous solution, Spinner et al. developed a NiO/ZrO_2_ nanocomposite, where ZrO_2_ served as the site for the adsorption of CH_4_ and NiO served as the site for carbonate ion adsorption to donate active oxygen species for activating C—H bonds in CH_4_.^[^ ^428^ ^]^ Importantly, NiO/Ni interface constructed by calcination was able to electro‐oxidate CH_4_ to alcohols, especially ethanol, as shown in Figure 20e.^[^ ^415^ ^]^ DFT studies have indicated that Rh nanoclusters with unsaturated coordinate atoms can keep •CH_3_ species in CH_4_ from further oxidation to CO_2_.^[^ ^429^ ^]^ In addition, it was proposed that ZnO could react as an active and selective electrocatalyst for electrochemical water oxidation to active O*.^[^ ^417^, ^430^ ^]^ Furthermore, the study of Li et al. indicates that CH_4_ may be electrochemically oxidized and coupled in superacid electrolytes at oxidized Pt electrodes. Moreover, Rocha et al. showed that the strong interaction between V_2_O_5_ and SnO_2_ conducted a partial reduction of V_2_O_5_ to V_4_^+^ species, which served as active sites of water oxidation to radical oxygen anion for CH_4_ activation;^[^ ^431^ ^]^ suggesting that singlet active oxygen species (O*) produced from water oxidation can activate and break the C—H bond of CH_4_ via radical routes.^[^ ^420^ ^]^ The CH_4_ activation at room temperature was also reported by Guo et al. via a porous nickel‐based hollow fiber electrode comprising a NiO active layer on the surface (NiO@NiHF).^[^ ^432^ ^]^

In contrast with aqueous electrolytes favoring CH_4_ oxygenation to C1 species via carbonates, the lack of chemical oxidants and the strong Brønsted nature of the superacid promoted CH_4_ activation at strongly Lewis‐acidic Pt^2+^ sites at room temperature. Even if Li et al. results are encouraging, work is still needed to better understand activation and coupling pathways. Likewise, it is vital to migrate from liquid superacid electrolytes from a safety and stability perspective.^[^ ^433^ ^]^ Meanwhile, Bunting et al. revealed that an aqueous medium has a significant impact on the kinetics and thermodynamics of selective CH_4_ oxidation, which can increase the selectivity by directing the reaction mechanism toward a C—OH coupling route rather than a C—O coupling route, minimizing the loss of selectivity to other oxygenates besides methanol.^[^ ^434^ ^]^ In addition, it was noticed that the use of water as the oxidant significantly reduces the thermodynamic driving force for the complete oxidation of CH_4_ relative to its partial oxidation to methanol.^[^ ^419^ ^]^ Wang et al. used intermediate chlorine species (*Cl), which were electrochemically generated and stabilized on mixed cobalt–nickel spinels with different Co/Ni ratios. The reported catalyst showed outstanding CH_4_ to CH_3_Cl activity (364 mmol g^−1^ h^−1^ at 2.3 V) and a high CH_3_Cl/CO_2_ selectivity in saturated NaCl electrolyte, which was attributed to the catalyst's ability to electrochemically produce *Cl, and Ni^3+^ at octahedral sites allowed to stabilize surface‐bound *Cl species better.^[^ ^435^ ^]^ Overall, electrocatalytic activation of CH_4_ under mild conditions is still early days; an ideal electrocatalyst system should consider the increase of CH_4_ conversion and selectivity toward the desired products.

### 3.4 Nonthermal Plasma Activation of Methane

**Figure 21:**
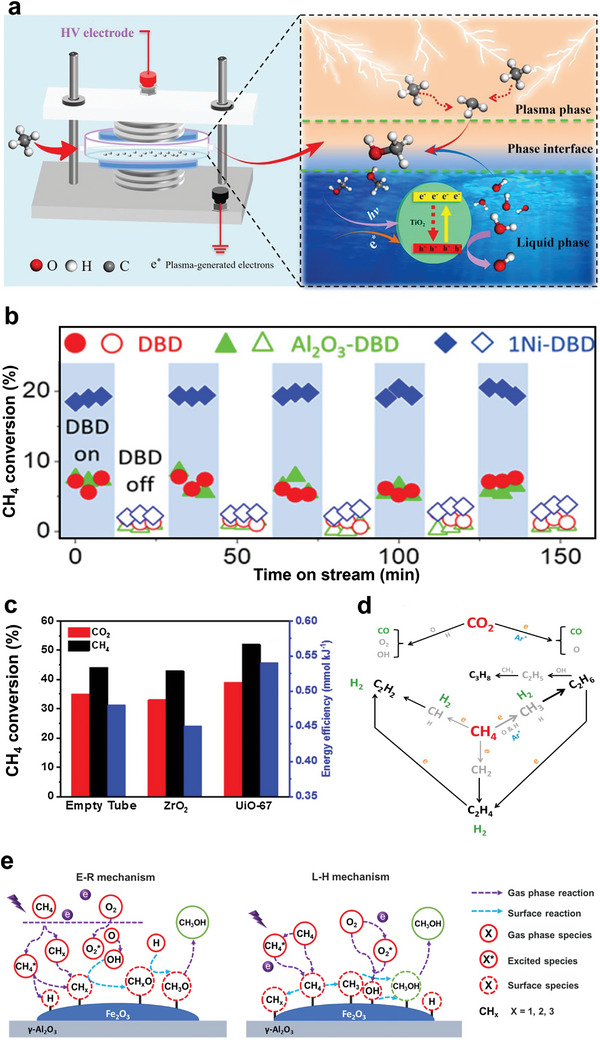
DBD system showing reaction between CH_4_ and H_2_O for methanol production. Reproduced under the terms of the Creative Commons Attribution 4.0 International License.^[^ ^440^ ^]^ Copyright 2022, The Authors, published by Springer Nature. Effect of DBD packing on b) CH_4_ conversions. Reproduced with permission.^[^ ^444^ ^]^ Copyright 2016, American Chemistry Society. c) On energy efficiency and CH_4_. d) Principal reaction routes in plasma‐aided catalytic DRM. Products are depicted in black (or green), whereas intermediates are indicated in gray. The color of metastable Ar is blue. Reproduced with permission.^[^ ^456^ ^]^ Copyright 2020, Elsevier. e) Proposed mechanistic pathways for the production of methanol using plasma on the Fe/*γ*‐Al_2_O_3_ surface. Reproduced with permission.^[^ ^458^ ^]^ Copyright 2021, Elsevier.

A plasma is an ionized gas that contains neutral species, ions, photons, and electrons. Unlike thermal plasma, nonthermal plasma (NTP) use electrical energy to generate highly energetic electrons and reactive species while keeping the gas kinetic temperature low.^[^ ^436^ ^]^ In addition, NTP can be upscaled easily and has long‐term stability.^[^ ^437^ ^]^ Thus, NTP may offer a way to defeat the kinetic and thermodynamic constraints on chemical transformations of reactants into final products without consuming energy in heating the system. Moreover, NTP can operate in conditions where the catalysts are generally inactive.^[^ ^438^ ^]^ Furthermore, NTP‐based processes can be powered by renewable electricity from wind, hydro, or solar power and stored in a chemical form.^[^ ^26^ ^]^ On the other hand, the high reactivity of plasma can inhibit the selective formation of the desired products.^[^ ^439^ ^]^ In addition, CH_4_ conversion still needs to be improved.^[^ ^440^ ^]^ As vibrational and electronic excitations activate the molecules in NTPs, various chemical reactions can occur, leading to their dissociation. NTP sources include dielectric barrier discharge (DBD), corona discharge, and spark discharge.^[^ ^441^ ^]^ **Figure** **21**a shows the DBD, one of the most widely utilized NTP sources for chemical reactions, because it is easily scaled up and combined with catalysts.^[^ ^26^, ^436^, ^440^, ^442^ ^]^ Indeed, a physically‐based model of the DBD reactor revealed that OH and O radicals produced by the dissociative electron‐impact reactions could boost CH_4_ conversion, making water a key abatement promoter in the plasma process.^[^ ^440^, ^443^ ^]^

For instance, the Ni–DBD combination led to CH_4_ conversions of 18–20%, as shown in (Figure 21b), emphasizing again the interaction between the DBD and Ni catalyst to boost the reaction.^[^ ^444^ ^]^ Jo et al. explored the activation of CH_4_ using NTP in the existence of various noble gas additives. The major products were alkane species formed independently of the noble gas in all cases. Nevertheless, the conversion of CH_4_ was significantly affected by the identity of the noble gas. To lower coke formation, which is a challenging issue, oxygen was added to the CH_4_ conversion process. Thus, the formation of carbon was inhibited, but the production of higher hydrocarbons was also significantly decreased. It was concluded that varying the discharge characteristics could enhance CH_4_ conversion, but the problem of carbon balance must be solved without adding oxygen.^[^ ^445^ ^]^

In fact, in the presence of plasma, the gaseous stream's ionization allows spontaneous CH_4_ dissociation, which leads to their conversion. However, due to several byproducts, such as coke, ethane, acetylene, and other hydrocarbons, the selectivity of the process is relatively low. This drawback limits the application of NTP to the reaction chemical processes. However, the combination of NTP technology with catalysts offers a viable option for activating CH_4_ and the possibility of easier breakage of the reactant molecules and improved selectivity toward hydrogen and carbon monoxide generation.^[^ ^446^, ^447^, ^448^ ^]^ In addition, it can help promote SO_2_ and H_2_O tolerance, as reported for In/H‐BEA.^[^ ^449^ ^]^ Because of the rapid development of the plasma–‐catalyst systems, previous attempts have been undertaken to review NTP‐catalysis for specific applications, including simulation methodologies for plasma–catalyst interactions,^[^ ^450^ ^]^ plasma reactor types, and operational conditions,^[^ ^451^ ^]^ and selective catalytic reduction (SCR) of NO*_x_*.^[^ ^452^ ^]^ As a result, this contribution will solely concentrate on the most recent developments in the NTP‐catalytic activation of CH_4_.

Various catalysts have been investigated to increase the CH_4_ conversion and the selectivity of main products. Ni as active metal and Al_2_O_3_ as a support are most studied.^[^ ^444^, ^453^ ^]^ Tu et al. examined the effect of various supports, including Al_2_O_3_, MgO, SiO_2_, and TiO_2_; their results revealed that the highest CH_4_ conversion and the lowest surface carbon deposition of 3.8% obtained with Ni/Al_2_O_3_ catalyst, which was due to higher Ni dispersion and stronger basic sites of Al_2_O_3_.^[^ ^454^ ^]^ Chawdhury et al. integrated an NTP using a DBD reactor with an M/SBA‐15 (M = Pd, Pt, Ag, and Au) catalyst, where the reduction of metal was carried out by H_2_ plasma treatment.^[^ ^455^ ^]^ In addition, Vakili et al. used NTP assisted with DBD packed with different catalysts, including ZrO_2_, UiO‐67 MOF, and PtNP@UiO‐67. The results showed that ZrO_2_ inhibited plasma generation while UiO‐67 enhanced it because of its porous nature, favoring filamentary micro‐discharges and surface discharges. Compared to the plasma‐alone mode, the enhanced plasma discharge improved the efficiencies of CH_4_ and CO_2_ by ≈18% and 10%, respectively, as shown in Figure 21c. Furthermore, in the UiO‐67 enhanced plasma‐assisted system, the hydrocarbon product distribution shifted from dominant C_2_H_6_ in the plasma‐alone mode to C_2_H_2_ and C_2_H_4_ (Figure 21d). Furthermore, no significant changes in the characteristics of UiO‐67 MOF were seen when varied treatment periods, discharge powers, and gases were used. Moreover, the PtNP@ UiO‐67 was reusable, and H_2_ production was enhanced by a high CH_4_/CO_2_ molar ratio and low feed flow rate.^[^ ^456^ ^]^ Gibson et al. investigated NTP‐assisted CH_4_ oxidation over Pd/Al_2_O_3_ by directly monitoring the catalyst's X‐ray absorption fine structure, which they combined with end‐of‐pipe mass spectrometry. Under NTP conditions, the catalyst did not exhibit any significant changes. Simultaneously, the NTP increased the Pd nanoparticles, despite the increasing temperature inadequate to ignite the thermal CH_4_ oxidation reaction.^[^ ^457^ ^]^ Interestingly, a combination of NTP and catalyst was also reported to enhance the selectivity of oxygenates. Using a coaxial DBD plasma reactor, Chawdhury et al. examined the effect of *γ*‐Al_2_O_3_ supported metal catalysts on plasma‐catalytic for partial oxidation of CH_4_ to liquid oxygenates. In the absence of a catalyst, the selectivity of oxygenates in the plasma reaction was 58.3%, but when DBD and catalysts were combined, oxygenate selectivity increased to 71.5%. The Fe/*γ*‐Al_2_O_3_ catalyst had the highest methanol selectivity (36.0%), whereas the Cu/Al_2_O_3_ catalyst enhanced the selectivity of C2 oxygenates to 9.4%, which is due to the presence of more acid sites on the Cu catalyst's surfaces. The surface CH_x_ species are essential for methanol synthesis because they can be produced via the direct adsorption of CH*_x_* radicals produced in the plasma gas‐phase reactions or by dissociating adsorbed CH_4_ (Figure 21e).^[^ ^458^ ^]^

Hu's DFT calculations and plasma kinetic modeling revealed that the energized electrons and ions generated by plasma could efficiently suppress the subsequent dehydrogenation of the adsorbed CH_3_, accelerating the desorption of the CH_3_ species from the catalyst surface and reducing the amount of carbon deposition on the catalyst surface.^[^ ^459^ ^]^ Thus, it is so apparent that the highly effective catalyst assisted by NTP is effective at increasing the CH_4_ conversion and reducing the carbon deposition, and it merits being widely used in catalysis. However, optical diagnostic and kinetics modeling investigated an innovative energy pooling mechanism for catalyst‐free CH_4_ activation at low temperatures enabled by the nanosecond pulsed NTP in argon and CH_4_ gas mixture. Except for direct electron impact dissociation during the pulse‐on phase, it was established that charge transfer between argon‐ion and CH_4_ and quenching of argon metastable species by CH_4_ contribute to an increase in hydrogen atom density during the pulse‐off period. The novel technique presented may help develop catalyst‐free and cost‐effective strategies for utilizing CH_4_ at low temperatures.^[^ ^460^ ^]^ The different reactive species produced in NTP can adsorb onto the catalyst, generating the desired products.^[^ ^436^, ^461^ ^]^ Few mechanistic investigations on the surface chemistry of NTP‐catalyzed CH_4_ oxidation are available. Zhang et al. studied the decomposition and oxidation of CH_4_ exposed to a Ni catalyst supported on an Al_2_O_3_/SiO_2_ support by time‐resolved DRIFTS. They noticed that treatment with Ar plasma produced surface‐bound CO, which was oxidized to CO_2_ when O_2_ was added to the plasma.^[^ ^462^ ^]^ A similar analysis (in situ DRIFTS) done by Stere et al. with a Pd/Al_2_O_3_ catalyst showed the significant formation of formate species (HCOO) on the catalyst related to CO_2_ formation. It has been proposed that CO and CO_2_ formation occur at different paths because of their varied formation profiles as a function of time.^[^ ^463^ ^]^ Using X‐ray absorption fine structure with Pd/Al_2_O_3_ catalyst in a plasma, Gibson et al. discovered that the catalyst did not exhibit any remarkable structural changes during operation.

Furthermore, the temperature of the Pd nanoparticles was lesser than that required to thermally activate the catalyst, suggesting a different route for CH_4_ oxidation.^[^ ^457^ ^]^ Loenders et al. have proposed a microkinetic model for CH_4_ activation via plasma catalysis that integrates the impacts of plasma species on catalyst surface chemistry, such as vibrationally excited molecules, radicals, and stable intermediates. The findings indicate that vibrational excitation improves the TOF of catalytic CH_4_ dissociation and has a high potential for enhancing selectivity toward CH_3_OH, HCOOH, and C2 hydrocarbons. However, when plasma‐generated radicals were also considered, it was discovered that these species mostly dominate surface chemistry. Furthermore, plasma‐generated radicals and stable intermediates improve the TOFs of CO*_x_* and oxygenate, augment the selectivity toward oxygenates, and boost the production of HCOOH on Pt (111).^[^ ^439^ ^]^ In summary, the combination of NTP with the catalyst has excellent potential in efficient CH_4_ conversion to liquid products and renewable oxygenates with limited carbon deposition. However, a low selectivity to the desired products and CH_4_ conversion remains the main challenge that needs much attention.

## 4 Challenges and Future Perspectives

CH_4_ activation at low temperatures is addressed as the “holy grail” of catalysis research due to the inert nature of CH_4_. Meanwhile, for low‐temperature CH_4_ oxidation, the thermal stability of the catalyst, and coke formation are out of concern. However, the catalytic performance of the CH_4_ activation depends on active sites located in a protective environment, like zeolite, microporous silica, and different high surface area supports. Even though considerable progress has been achieved in this field, there are still many challenges, such as catalyst design, reaction conditions, and a complete mechanistic understanding of catalytic processes, which also indicate opportunities for future research.

The catalyst preparation approach is one of the major factors influencing catalytic activity. Hence, developing a detailed synthesis technique including a new preparation procedure and integrating other synthesis methods, might be a viable option for preparing an excellent catalyst for CH_4_ activation at low‐temperature. The vapor deposition method can homogeneously introduce metal precursors into the micropores or on the support, providing homogeneous catalyst sites for the precise preparation of the catalysts. For instance, the combination of noble metals and transition metals in a precise way can result in higher CH_4_ conversion and lower reaction temperature. In addition, multimetal catalysts containing mixed oxide support and promoters in the mesoporous range are worth evaluating. Moreover, a fundamental understanding of nucleation and crystallization in zeolites and mechanistic insight on nanoparticle growth are helpful since the dynamics happening at the cluster scale is the missing piece of the puzzle for designing the catalysts for CH_4_ conversion at low temperatures. Recently, Bai et al. obtained 18% CH_4_ conversion at 550 °C under microwave irradiation, while 800 °C was required to achieve the same CH_4_ conversion level without microwave irradiation.^[^ ^464^ ^]^ Therefore, the combination of heterogeneous catalysis and microwave irradiation is attractive and is considered for further studies.

Noble‐metal catalysts are still primarily limited by H_2_O and SO_2_ poisons at low‐temperature. A possible solution would be in situ addition of materials that can capture poisons in the system. Furthermore, support materials that can make the catalyst resistant to water by bringing hydrophobicity into the system are strongly recommended. Modifying the support material might also be investigated to address issues related to hydrothermal stability or metal–metal strong interactions that make the catalyst inactive; this would improve the catalytic performance of the system under consideration. Meanwhile, the liquid heterogeneous system using environmentally friendly oxidants (H_2_O_2_) is regarded as the most promising area of research for activating the C—H bond at low temperatures. Thus, more effort should be concentrated on in situ generations of H_2_O_2_ or oxygen and hydrogen mixture gas as the green and inexpensive oxidant to enhance the CH_4_ conversion and increase the methanol selectivity. Concerning overoxidation of CH_3_OH, adding a small amount of scarifying agent to consume excess oxidant could be a solution. From an environmental point of view, the catalysts should be prepared with less hazardous substances, making their application easy on an industrial scale. In addition, to meet the needs of the industry, researchers must reduce the gap between laboratory‐scale experiments and the application of the prepared catalyst in full‐scale systems.

Given that the efficiencies of photocatalytic CH_4_ conversion remain low, and most photocatalysts reported for this conversion are restricted in UV‐responsive semiconductors, exploring catalysts with a more comprehensive spectral response is primary to realize the practical application. For instance, taking advantage of the synergistic effects between plasmonic metals and semiconductors would result in a catalyst that exhibits promoted photocatalytic properties for CH_4_ conversion than single semiconductors. In addition, constructing a 3D catalytic system with highly dispersed reactive sites encapsulated will be vital to improve the activity of solar‐driven CH_4_ conversion. In this context, MOFs and 3D graphene‐based hydrogels/aerogels featuring high porosity, high specific surface area, and abundant active sites are potential candidates for fabricating the stereoscopic catalytic units.

Furthermore, insights into supported metals’ coordination and electronic structures at the atomic level and dynamic changes in response to reaction conditions are needed to reveal exact structure–function relationships. In addition, the reaction mechanisms of photocatalysis and electrocatalysis systems are not well studied due to the difficulty in detecting the reaction intermediates involved in a complicated solid–liquid or solid–liquid–gas interface reaction system. Designing an in situ detection system that can also oxidize CH_4_ dispersed in the liquid under the high‐pressure gas phase might help shed light on the fundamental knowledge of the active sites. It is also worthwhile to investigate the disparities in efficiency between the conversion of H_2_O_2_ to •OH and the cleavage of the methane C—H bond for the various geometric and electronic structures of the support.

It is worth mentioning that the catalytic performance is affected by different factors, which are not standardized across various studies; hence, making in‐depth comparisons of the reported catalysts is still challenging. Therefore, it is suggested to build simple standard reaction conditions, which might be done based on real exhaust from natural gas engines. For instance, the reference provided by ACEC team^[^ ^465^ ^]^ clearly shows how to age, test, and expose catalyst to the poisons; however, it is difficult for researchers to possess all the required equipment to perform the suggested tests. Thus, a simple standard reaction condition might help to speed up the development of novel catalysts through deep studies correlating factors through machine learning and artificial intelligence. In addition, catalyst reusability, cost evaluation, and life‐cycle assessment studies at the industrial level are highly recommended, as they were never mentioned in all reviewed articles. Recently, a research team led by Dauenhauer reported a new theory (catalytic resonance theory) that uses waves to create an oscillating catalyst.^[^ ^466^, ^467^, ^468^, ^469^ ^]^ When the wave introduced to the catalyst surface corresponded to the natural frequency of a chemical reaction, the rate increased dramatically via a mechanism known as resonance. Hence, examining the applicability of dynamic heterogeneous catalysis for CH_4_ at low temperatures is highly recommended. Enhanced reaction rates for this new kind of catalyst could significantly reduce the amount of catalyst, the size of the equipment, and the overall costs, as well as reduce the amount of waste generated through catalyst application.

## 5 Conclusions

CH_4_, the most abundant hydrocarbon, is considered as an important greenhouse gas and an excellent carbon‐based fuel due to the lower carbon dioxide emissions per unit of energy, high thermal efficiency, and adaptability to standard engines. This review discussed the current progress for CH_4_ activation technologies focusing on the catalysts that can activate CH_4_ at a temperature below 500 °C. In the last half‐decade, significant advances have been achieved by developing supported metal catalysts in conjunction with several promoters such as metals, nonmetals, oxides, and zeolites. Other catalysts have been designed to have a core–shell structure that augments or protects the uniform dispersion of active metals.

The properties of various supports and promoters, like reduction/oxidation potential, acidity/basicity, reducibility, and oxygen storage capacity, have been used beneficially to lower the activation temperature of CH_4_. Metal oxide supports with high oxygen storage, and high reducibility is highly desirable for CH_4_ activation at low temperature to displace mobile oxygen and make vacancies. In addition, the nature of the exposed faces and crystal defects have a decisive effect on the catalyst performance. Along with this, theoretical models using DFT have been explored by various researchers to develop a basic knowledge of the profound chemistry at the molecular level for the characterizations of the active sites.

Noble metal catalysts showed good performance for CH_4_ activation at low temperatures compared to transition metal‐based catalysts. However, hydroxyl accumulation can reduce activity by disrupting oxygen transfer, and this impact is observed to be significant on support materials, which are heavily affected by water vapor, like *γ*‐Al_2_O_3_. Intensive research has been conducted using zeolite supports to impart hydrophobicity of the catalyst. These studies showed that zeolite‐supported catalysts improved the catalyst's efficiencies in wet conditions; however, these supports are frequently vulnerable to structure collapse when exposed to harsh aging treatments. The stability of zeolite support is improved by adjusting the Si/Al ratio and acidity, resulting in weaker water binding to active sites and improved catalytic life.

Via single‐atom catalysts, the use of H_2_O_2_ as oxidant can decrease the reaction temperature, while in situ synthesis of this oxidant from H_2_ and O_2_ is proved to be more efficient and economical in catalysis. In addition, the photocatalytic oxidation of CH_4_ demonstrates tremendous potential for promoting the direct conversion of CH_4_ into high‐value‐added chemicals. Furthermore, compared to thermal‐catalysis, the catalyst assisted by NTP demonstrated a clear promotion effect for CH_4_ conversion. Nevertheless, CH_4_ activation at low temperatures is still worth exploring; as discussed in the previous section, the current technology and methods can be improved in many aspects. Some new catalysts are attractive, but they still struggle to make a step forward from the laboratory to the industry. To achieve an optimum process for CH_4_ utilization, a great effort should be made by both the academic and industrial communities.

## Conflict of Interest

The authors declare no conflict of interest.
